# Discovery of a novel and highly selective JAK3 inhibitor as a potent hair growth promoter

**DOI:** 10.1186/s12967-024-05144-4

**Published:** 2024-04-18

**Authors:** Md Mehedi Hossain, Arfan Khalid, Zaheen Akhter, Sabra Parveen, Mir Owais Ayaz, Aadil Qadir Bhat, Neetu Badesra, Farheen Showket, Mohmmad Saleem Dar, Farhan Ahmed, Sumit Dhiman, Mukesh Kumar, Umed Singh, Razak Hussain, Pankaj Keshari, Ghulam Mustafa, Amit Nargorta, Neha Taneja, Somesh Gupta, Riyaz A. Mir, Aravind Singh Kshatri, Utpal Nandi, Nooruddin Khan, P. Ramajayan, Govind Yadav, Zabeer Ahmed, Parvinder Pal Singh, Mohd Jamal Dar

**Affiliations:** 1 Laboratory of Cell and Molecular Biology, Pharmacology Division, CSIR-Indian Institute of Integrative Medicine, Jammu, Jammu and Kashmir 180001 India; 2 Academy of Scientific & Innovative Research, Ghaziabad, Uttar Pradesh 201002 India; 3 Pharmacology Division, CSIR-Indian Institute of Integrative Medicine, Jammu, Jammu and Kashmir 180001 India; 4 Natural Products and Medicinal Chemistry Division, CSIR-Indian Institute of Integrative Medicine, Jammu, Jammu and Kashmir 180001 India; 5 Medicinal Product Chemistry, Sussex Drug Discovery Centre, School of Life Sciences, University of Sussex, Falmer, Brighton, BN1 9QG UK; 6 Department of Entomology, University of Illinois Urbana-Champaign, Urbana, IL 61801 USA; 7 Department of Biochemistry, All India Institute of Medical Sciences, New Delhi, India; 8 Department of Dermatology and Venereology, All India Institute of Medical Sciences, New Delhi, India; 9 Division of Neuroscience and Ageing Biology, CSIR-Central Drug Research Institute (CDRI), Lucknow, 226031 India; 10 Department of Animal Biology, School of Life Sciences, University of Hyderabad, Gachibowli, Hyderabad, 500046 India

**Keywords:** Alopecia areata, Hair follicles, JAK-STAT signalling, Tofacitinib, Baricitinib, Ritlecitinib, 3-pyrimidinylazaindole

## Abstract

JAK-STAT signalling pathway inhibitors have emerged as promising therapeutic agents for the treatment of hair loss. Among different JAK isoforms, JAK3 has become an ideal target for drug discovery because it only regulates a narrow spectrum of γc cytokines. Here, we report the discovery of MJ04, a novel and highly selective 3-pyrimidinylazaindole based JAK3 inhibitor, as a potential hair growth promoter with an IC_50_ of 2.03 nM. During in vivo efficacy assays, topical application of MJ04 on DHT-challenged AGA and athymic nude mice resulted in early onset of hair regrowth. Furthermore, MJ04 significantly promoted the growth of human hair follicles under ex-vivo conditions. MJ04 exhibited a reasonably good pharmacokinetic profile and demonstrated a favourable safety profile under in vivo and in vitro conditions. Taken together, we report MJ04 as a highly potent and selective JAK3 inhibitor that exhibits overall properties suitable for topical drug development and advancement to human clinical trials.

**Supplementary Information:**

The online version contains supplementary material available at 10.1186/s12967-024-05144-4.

## Introduction

Alopecia areata is an autoimmune disease that occurs when the immune system attacks hair follicles and causes them to lose the ability to enter the growth phase of the hair growth cycle, ultimately resulting in hair loss [1]. Hair follicles are mini-organs comprising of many different cell types, including hair follicle stem cells (HFSCs) [2]. Various studies have shown that hair loss is associated with the production of inflammatory cytokines by certain cells in the hair follicle, which then causes progressive hair thinning and miniaturization of these hair follicles [3]. Hair follicles were previously thought to be missing from the areas of complete baldness, however, recent studies have shown that even the bald areas of the human scalp contain hair follicles and hair follicle stem cells, thus leading to exploring new ways of restoring hair growth [4]. In humans, the hair growth cycle has four different stages or phases [5]. The anagen phase is the period of active hair growth. This phase lasts 3–5 years and determines the hair length on the scalp [6]. This is followed by a short transitional catagen phase during which hair follicles shrink and detach. In the case of scalp hair, this phase lasts 1–2 weeks. Telogen and exogen phases last for around 3 months [7]. During this period, old hair ceases growing, and new hair begins to enter into the growth phase. During the anagen phase, also called the growing phase, hair cells have a high mitotic rate and are thus considered among the most rapidly replicating cells in humans [8]. Approximately 88% of the hair on the scalp is in the anagen phase, 1% in the catagen, and the remainder in the telogen phase under normal circumstances [9]. Limited effectiveness and side effects of currently available pharmacological agents and surgical procedures employed to restore hair regrowth have raised the demand for the discovery and development of new therapeutic agents. Recently, baricitinib, tofacitinib, and ritlecitinib as pharmacological inhibitors of the Janus Kinase Signal Transducer and Activator of Transcription (JAK-STAT) pathway have been reported to promote hair growth both in mice and humans [10, 11]. The JAK-STAT signalling pathway is primarily involved in regulating cell proliferation, differentiation, stem cell maintenance, cytokine signalling, and regulation of the immune system [12]. Out of nine JAK inhibitors that have been approved by the FDA for the treatment of many diseases, baricitinib was approved for the treatment of hair loss in 2022 and ritlecitinib in 2023 [13, 14]. Here, we present the discovery of MJ04 as a potent JAK-STAT signaling inhibitor involved in promoting hair regrowth in mice and humans. MJ04 has an advantage over non-selective JAK inhibitors like baricitinib, ruxolitinib, and tofacitinib that it only inhibits γc chain receptor-mediated signalling. MJ04 with an IC_50_ of 2.04 nM is highly potent in comparison to ritlecitinib (IC_50_ of 33.1 nM for JAK3) and baricitinib (IC_50_ of 5.9 nM and 5.7 for JAK1 and JAK2) [15, 16]. More importantly, unlike baricitinib and ritlecitinib that are approved for oral use, topical administration of MJ04 showed reasonably good efficacy and safety profile without any adverse effects.

## Material and methods

### Cell lines, reagents, and antibodies

Human normal HEK-293 (human embryonic kidney cell line) and A549 (human lung cancer cell line), HCT-116 (human colon carcinoma cell line), PC-3 (human prostate carcinoma cell line), MCF-7 (human breast cancer cell lines), and Mia PaCa-2 (human pancreatic cancer lines) cells were purchased from the National Centre for Cell Science (NCCS), Pune, India. A-549 and HCT-116 cells were grown in RPMI-1640 (Sigma, Cat No.- R6504) medium supplemented with 10% fetal bovine serum (FBS), penicillin (100 units/ml), streptomycin (100 mg/ml), L-glutamine (0.3 mg/ml), sodium pyruvate (550 mg/ml), and NaHCO_3_ (2 mg/ml). All other cell lines were grown in DMEM (Sigma, Cat No.-D7777) and supplemented with 10% fetal bovine serum (FBS Qualified: Standard origin Brazil 10,270,106). Murine macrophages (J774a.1) were purchased from ATCC. The GAPDH (SC-166574) antibodies were purchased from Santa Cruz Biotechnology Inc. The Phospho-JAK Family antibody sampler kit (CST-97999 T), phospho-STAT antibody sampler kit (CST-9914 T), and STAT antibody sampler kit (CST-9939 T) were purchased from Cell Signaling Technology (Danvers, MA). tofacitinib citrate (Sigma, Cat No-PZ0017), baricitinib (Tocris Bioscience, Cat No- 7222), PEG-300 (SD FINE chemical Ltd), Testosterone (Hi-Media, Cat No.—RM1848). EGFR kinase enzyme system (Promega Corporation, Cat No.-V3831), GSK-3-β kinase enzyme system (Promega Corporation, Cat No.-V1991), IGF1R kinase enzyme system (Promega Corporation, Cat No.-V3581), JAK1 kinase enzyme system (Promega Corporation, Cat No.-VA7207), JAK2 Kinase enzyme (BPS Bioscience, Cat No.- 79,520), JAK3 kinase enzyme system (Promega Corporation, Cat No.-V3701), PI3K-Glo™ Class 1 profiling kit (Promega Corporation, Cat No.-V1690) were purchased. The mouse enzyme-linked immunosorbent assay (ELISA) kits of TNF-α (Cat No.-560,478), IL-6 (Cat No.-550,950), IL1-β (Cat. No.-559,603), and BD OptEIA™ TMB Substrate Reagent Set (Cat No.-555,214) were purchased from BD Biosciences. All other reagents and chemicals were purchased from Sigma-Aldrich.

### Cell-free kinase assays

The cell-free kinase assays of small molecules were determined by following the manufacturer-recommended protocols. Different concentrations in triplicate were used to determine the IC_50_ value of the hit molecules (active molecules). For data analysis, GraphPad Prism V9.5 Software and a sigmoidal dose–response (variable slope) equation were used. Each value represents an average of three replicates per concentration.

### Cell viability assay

Cell viability was evaluated using an MTT colorimetric assay [17]. Cells were seeded (10^5^ cells/mL) in 96-well plates and allowed to grow overnight at 37 ℃ with 5% CO_2_ in a cell-culture incubator. On the following day, the cells were treated with small molecules at different concentrations (0.2–10 micro-molar) or vehicle (DMSO). Cells were then further incubated for 48 h. The cells in each well were then incubated with 20 μL of MTT dye (2.5 mg/mL for 4 h at 37 ℃. After 4 h, the media was discarded completely, and crystals were dissolved in 150 μL of DMSO, and reading was taken in a spectrophotometer at 570 nm using a multi-well plate reader (Tecan Infinite M Nano-Infinite 200Pro). Inhibitory concentration (IC_50_) values were obtained using GraphPad Prism V9.5 (GraphPad Software). All data are shown as the mean value ± SD of three independent experiments. The cell viability percentage and growth inhibition rate were computed by using the following formula.

$$\mathrm{\% Cell\, viability}\hspace{0.17em}=\hspace{0.17em}\frac{\mathrm{Absorbance\, of\, treated\, cells}-\mathrm{ Absorbance\, of\, Blank}}{\mathrm{Absorbance\, of\, untreated\, cells}-\mathrm{ Absorbance \,of\, Blank}}$$

$$\% {\text{ Growth Inhibition}}\, = \,{1}00 - \% {\text{ cell viability}}$$

### Docking and MD simulation

The protein PDBs for the kinase domain of JAK1 (3EYG), JAK2 (6TPD), and JAK3 (5WFJ) were downloaded from RCSB PDB database (https://www.rcsb.org), and the 3D structure of ligand (inhibitor) MJ04 was obtained from PubChem database for small molecules (https://pubchem.ncbi.nlm.nih.gov). PyMOL removed the native ligand (inhibitor) molecule present in the PDBs, the water molecules were also removed, and each PDB was energy minimized by GROMACS [18]. The molecular docking of inhibitor molecule MJ04 and respective PDBs of JAK1 (3EYG), JAK2 (6TPD), and JAK3 (5WFJ) was performed by AutoDock Vina [19]. After molecular docking, each complex was subjected to molecular dynamics (MD) simulation of 10 ns by GROMACS [18] to analyze the potential interaction of inhibitor MJ04.

### Western blotting

For western blotting, A549 cells were seeded at a density of 0.2 × 10^6^ cells/well in 6-well plates. At 24 h, cells were treated with various concentrations of small molecules and grown with 10% FBS for 48 h. Whole-cell lysates were prepared in 1× RIPA buffer (Sigma) with added sodium orthovanadate (1 mM), protease cocktail (Roche), NaF (1 mM), PMSF (1 mM) and EDTA (0.5 M). Protein estimation was done using Bradford reagent (Bio-Rad). The samples were boiled with sample buffer containing 1% β-mercaptoethanol, 6% glycerol, 2% SDS, 22 mM Tris–HCl pH 6.8, and bromophenol blue. Whole-cell lysates corresponding to 70-100 µg of protein were loaded. The samples were analysed by SDS-PAGE with a 10% separating gel. After running the gel, the proteins were transferred to PVDF membrane (Millipore) and then blocked for 1 h in a solution of 5% BSA (Sigma), 0.1% Tween 20 (Hi-Media), 150 mM NaCl and 20 mM Tris–HCl pH-7.4. The membrane was probed with specific antibodies for pJAK-1 (1:1000), p-STAT-3 (CST) (1:1000), total JAK-1 (1:1000), pJAK-3 Z(1:1000), T-JAK-3 (1:1000), total STAT-3(1:1000), GAPDH (1:500), and visualized using ECL (Millipore). GAPDH was used as a loading control. The results shown are representative of western blot data analysis obtained from at least three independent experiments. To induce STAT3 pathway stimulation with IL-6, A549 cells were initially seeded in 6 well-culture plates at a concentration of 1.25 × 10^5^ cells/mL and incubated for 24 h. Subsequently, the cells were subjected to serum-free medium for a minimum of 12 h to induce starvation. Following this starvation period, the A549 cells were pre-treated with varying concentrations of MJ04 for a duration of 4 h. Following the pre-treatment, the cells were stimulated with 10 ng/mL of IL-6 for 30 min [20–22].

### Animals and ethics

The experiments were performed on C57BL/6 J and Athymic nude mice (NU/J *Foxn*1^nu^) of 6–7 weeks of age obtained from Animal House Facility, CSIR-Indian Institute of Integrative Medicine (IIIM), Jammu, India. The mice were housed under controlled environmental conditions (Temp-25 ± 2 ℃, RH-65 ± 5%, 12 h/12 h light/dark cycle) with 3 animals per cage. The animals were provided with ad libitum feed (standard pellet diet) and autoclaved RO water throughout the study. The body weight of mice was measured after seven days before treatment. All experimental procedures were approved by the Institutional Animal Ethics Committee (IAEC) of CSIR-IIIM (IAEC approval No: 290/80/2/2022) and performed in accordance with the guidelines of the Committee for Control and Supervision of Experiments of Animals (CCSEA https://ccsea.gov.in).

### Experimental design for C57BL/6 mice

C57BL/6 J mice (male) were obtained from CSIR-IIIM Animal House at 7 weeks of age. We used a DHT-induced AGA mice model for this study [23]. Mice were shaved dorsally using electric shaver and hair removal on postnatal day 43 (telogen) and topically treated with vehicle (equal volume of PEG-300 and ethanol), 0.5% testosterone (dissolved in 50% ethanol), two different concentrations of tofacitinib citrate (dissolve in vehicle), three different concentrations of MJ04 (dissolved in vehicle), and three different concentrations of baricitinib (dissolved in vehicle) for 28 days. Animals were divided into eleven groups ( n = 8 per group), namely Group 1—No treatment, Group 2—treated with 0.5% testosterone, Group 3—treated with 0.5% testosterone followed by vehicle treatment after 1 h, Group 4—treated with 0.5% testosterone followed by tofacitinib citrate (0.8 mg/Kg) treatment after 1 h, Group 5—treated with 0.5% testosterone followed by tofacitinib citrate (0.08 mg/Kg) treatment after 1 h, Group 6—treated with 0.5% testosterone followed by MJ04 (0.08 mg/Kg) treatment after 1 h, Group 7—treated with 0.5% testosterone followed by MJ04 (0.04 mg/Kg) treatment after 1 h, Group 8—treated with 0.5% testosterone followed by MJ04 (0.016 mg/Kg) treatment after 1 h, Group 9—treated with 0.5% testosterone followed by baricitinib (0.1 mg/Kg) after 1 h, Group 10—treated with 0.5% testosterone followed by baricitinib (0.04 mg/Kg) after 1 h, and Group 11—treated with 0.5% testosterone followed by baricitinib (0.02 mg/Kg) after 1 h. At the end of the experiment, skin samples were collected and preserved in 10% neutral buffered formalin for tissue sectioning and staining using haematoxylin and eosin (H&E). The histopathological examinations were performed under the 10× and 40× objectives of light microscope (Magnus, Mx21i).

### Experimental design for athymic nude mice

Mice (male, n = 5 per group) were topically treated with vehicle control (equal volume of PEG-300 and ethanol), two concentrations of tofacitinib citrate, three concentrations of MJ04, and three concentrations of baricitinib for 28 days. The groups are as follows: Group 1-Control (untreated), Group 2-Vehicle control, Group 3-tofacitinib citrate (0.8 mg/Kg), Group 4-tofacitinib citrate (0.08 mg/Kg), Group 5-MJ04 (0.08 mg/Kg), Group 6-MJ04 (0.04 mg/Kg), Group 7-MJ04 (0.016 mg/Kg), Group 8-baricitinib (0.1 mg/Kg), Group 9-baricitinib (0.04 mg/Kg), and Group 10-baricitinib (0.02 mg/Kg). At the end of the experiment, skin samples were collected and preserved in 10% neutral buffered formalin for tissue sectioning and staining using haematoxylin and eosin (H&E). The histopathological examinations were performed under the 10× and 40× objectives of light microscope (Magnus, Mx21i), and hair follicles were graded on a scale of 0 to 4 (++++).

### Experimental design for cultivating human hair follicles in ex-vivo environments

Hair follicles were collected from three single donors of androgenetic alopecia undergoing hair transplant surgery at the Department of Dermatology, All India Institute of Medical Sciences, New Delhi, India. All experimental procedures were approved by the Institutional Ethics Committee (IAEC) of AIIMS, New Delhi (IEC approval No: 550/01.09.2023). Donors were above 18 years of age. Isolated hair follicles were cultured as per the previously described method of Philpott et al*.* [24, 24]. Immediately after isolation, follicles were placed in 24 well plates under sterile conditions into ice-cold Williams’ medium E (Sigma) with the different concentrations of MJ04 for 20 days. Half media was replaced with fresh media every 3rd day for 20 days. Basal length, i.e., length on the day of starting culture and post treatment was measured. Photographs were taken with a Nikon digital camera.

### Acute dermal toxicity and acute dermal irritation study of MJ04

The acute dermal toxicity was conducted in Wistar rats to determine the dermal LD_50_ of MJ04 adhering to Organization for Economic Cooperation and Development (OECD) test guideline 402. A single dose of MJ04 covering 10% of body surface area was administered using a fixed dose procedure from 200 mg/kg to 2000 mg/kg with two animals per dose. The animals were monitored for mortality, morbidity, and clinical signs of toxicity at first 30 min, 6 h, 24 h, and thereafter for 14 days. Parameters like body weight, serum biochemistry (liver function test, kidney function test, and lipid Profile), and histopathology were analysed. The acute dermal irritation test was conducted in C57BL/6J mice at a single dose of 0.04 mg/kg and 0.08 mg/kg of MJ04. The animals were observed for skin reactions, and digital photographs were taken at various time points (0 h, 5 min, 1 h, 4 h, 24 h, and 14 days) from representative areas using a Nikon digital camera (n = 5 mice).

#### Histopathological analysis of C57BL/6J MICE

Dorsal skin samples from C57BL/6J mice were shaved and collected for histological and molecular analyses on Days 7, 14, 21, and 28. The full-thickness skin tissue was fixed in a 10% neutral buffered formalin solution (Sigma) overnight and subsequently dehydrated for embedding in paraffin. Haematoxylin and eosin (H&E) staining was performed on the skin tissue, and digital photomicrographs were taken from the representative areas using light microscope (Magnus, Mx21i).

#### Histopathological analysis oF athymic nude mice (NU/J Foxn1^nu^)

The skin samples collected from athymic nude mice (NU/J Foxn1^nu^) were preserved in 10% neutral buffered formalin and processed for tissue sectioning. The skin sections (10 µm) of nude mice were stained using haematoxylin and eosin (H&E) and viewed under 10× and 40× objectives of the light microscope (Magnus, Mx21i). The histopathological examination of the samples was performed, and the hair follicles in the skin sections were graded on a scale of 0 to 4 (++++).

#### Histopathological analysis of Wistar rat

In this experimental procedure, full-thickness skin, kidney, liver, heart, and brain tissue samples obtained from Wistar rats were initially fixed and preserved in a 10% neutral buffered formalin solution. Subsequently, these were subjected to dehydration processes to enable paraffin embedding. The tissue sections, each measuring 10 μM in thickness, were further subjected to staining utilizing haematoxylin and eosin (H&E) protocols. These stained sections were then observed under a light microscope (Magnus, Mx21i). The histopathological images presented in Fig. 6D, magnified at 10×, offer visual insights into the skin, kidney, liver, heart, and brain tissue samples derived from both the control (a and b) and MJ04 (2000 mg/Kg) (c and d) experimental groups (Fig. 6D and Additional file 1: Fig. S6A).

### Haematological analysis

Every two weeks during the experimental period, mice were anesthetized, and the blood samples were collected into tubes containing heparin sodium to detect the total and differential white blood cells, haemoglobin, red blood cell, platelet count, mean corpuscular haemoglobin, mean corpuscular volume, red blood cell volume, mean platelet volume, and analyzed using an automated haematology analyzer (Model: XT1800i; Make: Sysmex).

### Biochemical analysis

Every two weeks during the experimental period, mice and rats were anesthetized, and blood samples were collected into non-heparinized tubes and then centrifuged at 1000 rpm at 4 °C for 10 min to obtain the serum. The biochemical parameters, including total protein, aspartate aminotransferase, alanine aminotransferase, total protein, albumin, total bilirubin, direct bilirubin, glucose, total cholesterol, triglyceride, low-density lipoprotein, high-density lipoprotein, creatinine, and uric acid, were measured using an automatic biochemical analyzer (Model: EM360; Make: Erba Mannheim).

### Hair cycle analysis

The quantification of anagen initiation was assessed by observing changes in the dorsal skin colour of C57BL/6 mice, a phenomenon closely linked to the onset of the anagen phase. Anagen quantification was done by employing threshold analysis on dorsal skin colouration using ImageJ. The darkening of the skin, attributed to melanogenesis, strongly correlates with the progression of anagen. This analysis is visually depicted as a heatmap using GraphPad Prism 9.5 Software [25].

### Isolation of splenocytes and T-cell differentiation

Splenocytes were isolated from the spleen of C57BL/6 mice. The whole spleen was crushed in a complete RPMI medium and passed through the 40 µm cell filter to obtain a single-cell suspension. The cell suspension was centrifuged at 300×*g* for 5 min, and the pellet was resuspended into the 1× RBC lysis buffer (Life Technology, Thermofisher, USA) for 10 min at room temperature to lyse the red blood cells. The sample was further centrifuged at the same speed and duration, and the pellet was resuspended in complete RPMI medium. Now, splenocytes were seeded into the 96-well plates (U bottom) at 3 × 10^5^ cells/well density. Cells were stimulated with anti-CD3 (10 µg/ml) and anti-CD28 (10 µg/ml) in the presence or absence of MJ04 at different concentrations (0.01, 0.1, 1, and 10 µM). For Th1 and Tc1 differentiation, IL-12 (5 ng/ml) was added and allowed to grow for four days.

### Immunophenotyping by flow cytometry

Prior to harvesting, cells were further stimulated with PMA (50 ng/ml), ionomycin (1000 ng/ml), and glogi Stop (as per manufacturer recommendation) for 4 h. The plate was spun down at 2000 rpm for 10 min and washed once with FACS buffer (3% FBS, 1× PBS, and 200 mM EDTA). Cells were stained with anti-CD4-PerCP and anti-CD8-FITC antibodies (BD, Biosciences) in the dilution 1:400 by adding 100 µl/well and incubated at RT for 60 min. The plate was washed again, and cells were fixed with 4% PFA (100 µl/well) for 20 min at RT and further washed and permeabilized with 100 µl/well perm buffer (Biolegend) for another 20 min at RT. Now intracellular staining of IFN-γ was performed by adding (100 µ/well) anti-IFN-γ-APC in 1:200 dilution (BD Biosciences) for 60 min at RT. Cells were finally washed and resuspended into FACS buffer, and data was recorded into the BD accuri C6 flow cytometer, and data were analyzed by Flowjo.

### Measuring the level of cytokines from the LPS primes macrophages

Murine macrophages (J774a.1 from ATCC) were plated in the 96 well-plate (1 × 10^6^/ml); after 12 h, cells were treated with LPS (500 ng/ml) for another 12 h in the presence and absence of MJ04 (0.01, 0.1, 1 and 10 µM) and tofacitinib. The supernatant was collected and stored at −20 ℃ for further cytokines analysis. The level of cytokines (TNF-α, IL1-β, and IL-6) was analyzed by sandwich ELISA as per the manufacturer protocol (BD Biosciences). In brief, capture antibodies were coated in the 96-well ELISA plate (Nunc) in respective coating buffer at 4 ℃ overnight. The plate was washed 4 times with washing buffer and blocked with the recommended blocking buffer for 1 h at room temperature. After incubation, plates were washed 4 times, added with sample and standard, and further incubated for another 2 h. Plates were then washed 4 times, added with detection antibody in combination of HRP for 1 h, and finally again washed 5 times. HRP substrate (TMB) was added and allowed to develop observable colour, and the reaction was stopped by adding 2 N H_2_SO_4,_ and absorbance was recorded at 450 nm with 570 nm correction.

### Acute dermal toxicity assessment of MJ04

Signal dose acute dermal toxicity tests were conducted in C57BL/6 mice. The tests included limited dosages of a single dose at 0.04 mg/kg and 0.08 mg/kg of MJ04. Notably, no mortality or clinical signs of toxicity were observed at 0.04 mg/kg and 0.08 mg/kg doses. Digital photographs were taken at various time points (0 min, 5 min, 1 h, 4 h, 24 h, and 14 days) from representative areas using a Nikon digital camera (n = 5 mice).

### Median lethal dose (LD_50_) determination

Acute dermal toxicity tests were also conducted in Wistar rats, following OECD 402 guidelines. A single dose of MJ04, covering 10% of the body surface area, was administered using a fixed-dose procedure. Limited testing included a single dose at 2 g/kg body weight (Fig. 6C).

The acute dermal toxicity of MJ04 showed no mortality and clinical signs of toxicity at 2000 mg/kg body weight. All other parameters (body weight, serum biochemistry, gross histopathological findings) were comparable with the control group (Fig. 6D–F). Based on the results, the LD_50_ value of MJ04 was found to be greater than 2000 mg/kg body weight (> 2000 mg/kg b.w.) under tested conditions and classified as Category-5 as per the Globally Harmonized Classification System (GHS) for Chemical Substances and Mixtures.

### Pharmacokinetic study

The male C57BL/6 mice were used to study the pharmacokinetics of MJ04. MJ04 was dissolved in vehicle suspensions for dermal application (30 mg/kg). Blood samples were collected at different time points: 0 min, 30 min, 1 h, 2 h, 4 h, 8 h, 12 h, and 24 h after dermal application. Plasma samples were separated by centrifugation of whole blood and stored at less than − 70 °C until bioanalysis. The concentration of MJ04 in plasma was detected using LC–MS/MS (Agilent Technologies, 1260 Infinity system). The pharmacokinetic parameters were processed by DAS (version 3.2.8) software.

### Metabolic stability

Metabolic stability assessment was conducted using human liver microsomes (HLM) through a substrate-depletion technique, following established procedures [26]. To elaborate, a phosphate buffer (100 mM, pH 7.4) containing magnesium chloride (3.3 mM) and human liver microsomal protein (HLM at a concentration of 0.5 mg/mL) was pre-incubated for various durations: 0 min, 5 min, 15 min, and 30 min, within a preheated shaking water bath set at 37 °C. Subsequently, the pre-incubated mixture was augmented with MJ04 (at a concentration of 5 μM) and NADPH (at 1.2 mM), followed by additional incubation in the preheated shaking water bath at 37 °C for 30 min, with constant shaking at 120 rpm. These incubations were conducted in triplicate, while samples without NADPH were negative control. The samples were then processed by introducing chilled acetonitrile (100 μL), followed by vigorous vortex mixing for 2 min. Subsequently, the samples were centrifuged at 3000 rpm for 15 min, and the resulting supernatant was transferred to vials for analysis using liquid chromatography-mass spectrometry (LC–MS/MS).

### Stability analysis of the formulation used for in vivo studies

The stability study of the lead compound (MJ04) was performed using HPLC analysis wherein the samples were studied after different time intervals. The 0.6 mg of MJ04 was taken and dissolved in 1 mL of ethanol to achieve 0.6 mg/mL concentration. Out of this, 500 µL was further diluted to 0.3 mg/mL by adding 500 µL ethanol to it (Ethanol sample). To remaining 500 µL, we added 500 µL PEG-300 to achieve final concentration of 0.3 mg /mL (Ethanol:PEG sample). HPLC of these samples was performed at various time points (0 h, 12 h, 24 h and 48 h) upon incubating at room temperature. The HPLC of samples was performed by RP-18 end capped; 5 µM, 4.6 × 250 mm. The mobile phase containing water (30%) and acetonitrile (70%) was used at the flow rate of 1 mL/min at a column temperature of 27 °C at 210 nm wavelength. A volume of 5 µL of sample was injected and the total run time of the assay was 30 min using the isocratic technique in the HPLC system.

### hERG channel assay

HEK293 cells (BPS Bioscience, San Diego, United States) stably expressing hERG channels (human ether-à-go-go-related gene, K_v_11.1) were used for electrophysiological recordings. These cells were cultured in minimal Essential Medium (MEM) supplemented with 10% fetal bovine serum, 1% non-essential amino acids, 1% Na pyruvate, 1% penicillin/streptomycin, and 400 μg/ml geneticin. hERG currents were recorded using the whole cell patch clamp technique ([27] at room temperature (22–23 °C). Whole-cell currents were amplified with EPC-10patch-clamp amplifier controlled via PatchMaster software (HEKA Elektronik). Data were acquired at 100 kHz and low-pass filtered at 5 kHz with 4-pole Bessel filter. The voltage protocol consisted of a depolarization of the cell membrane to + 20 mV for 2 s (for activation of channels) from a holding potential of − 60 mV andupon subsequent repolarization to − 40 mV for 3 s to evoke the tail current. This protocol was run at least 10 times at intervals of 10 s for each drug concentration. The capacitive currents were cancelled and series resistance was compensated at 70% with the amplifier. Pipettes were fabricated from thick-wall borosilicate glass (1.5 mm O.D. × 0.86 mm I.D.) using Sutter P-1000 puller and fire polished. The obtained pipettes had a resistance of 2–5 mΩ when filled with recording solution, which contained (in mM): 130 KCl, 5 EGTA, 10 HEPES, 5 Mg ATP, 1 MgCl_2_ ∙ 6 H_2_O, pH 7.2 with KOH. The bath solution contained (in mM): 137 NaCl, 4 KCl, 10 HEPES, 10 Sucrose, 1.8 CaCl_2_ ∙ 2 H_2_O, 1 MgCl_2_ ∙ 6 H_2_O, pH 7.4 with NaOH. Following the initial period of stable current recordings in bath solution, cells were superfused with the compounds at the concentrations as indicated in the results. 25 mM stock of test compounds were prepared in 100% DMSO and subsequently, the compounds were resuspended in extracellular solution to achieve the desired concentration. A complete cumulative dose response analysis was accomplished per cell, starting with the lowest concentration (0.3 μM) to highest concentration (30 μM). After the highest concentration of the test compound was applied, 0.1 μM E-4031 was perfused to demonstrate sensitivity of the procedure. Only data from cells treated with the test item, vehicle (0.1% DMSO, Control) or E-4031 were documented. Each test condition was evaluated in three separate experiments. All data were expressed as the mean ± SEM.

### ADMET prediction

ADMET, which encompasses absorption, distribution, metabolism, excretion, and drug toxicity, represents the five pivotal facets of pharmacokinetics in drug research and development [28]. To reduce attrition in drug discovery and development, we conducted ADMET screening. This screening aimed to identify inhibitors that align with pharmacodynamic requirements. The assessment of pharmacokinetic properties and potential drug toxicity in human subjects was accomplished using web-based tools- Swissadme (http://www.swissadme.ch/index.php) [29] and ADMETlab 2.0 (https://admetmesh.scbdd.com/service/screening/index) [30]. The assessment covered several critical parameters, including human intestinal absorption (HIA), plasma protein binding (PPB), blood–brain barrier (BBB) permeability, interaction with cytochrome P450 CYP2D6, and potential hERG Blocker effects. These evaluations are integral to ensuring the safety and efficacy of drug candidates within the drug development pipeline.

### Statistical analysis

Data analysis was done using Adobe Photoshop 7.0, MS Excel, and GraphPad Prism 9.5 Software. All data analysis was done using GraphPad Prism 9.5 Software and Image-J. Statistical significance of data was determined by one-way analysis of variance (ANOVA) and was accepted at P < 0.05.

## Results

### Identification of MJ04 as a selective JAK3 inhibitor

**Fig. 1:**
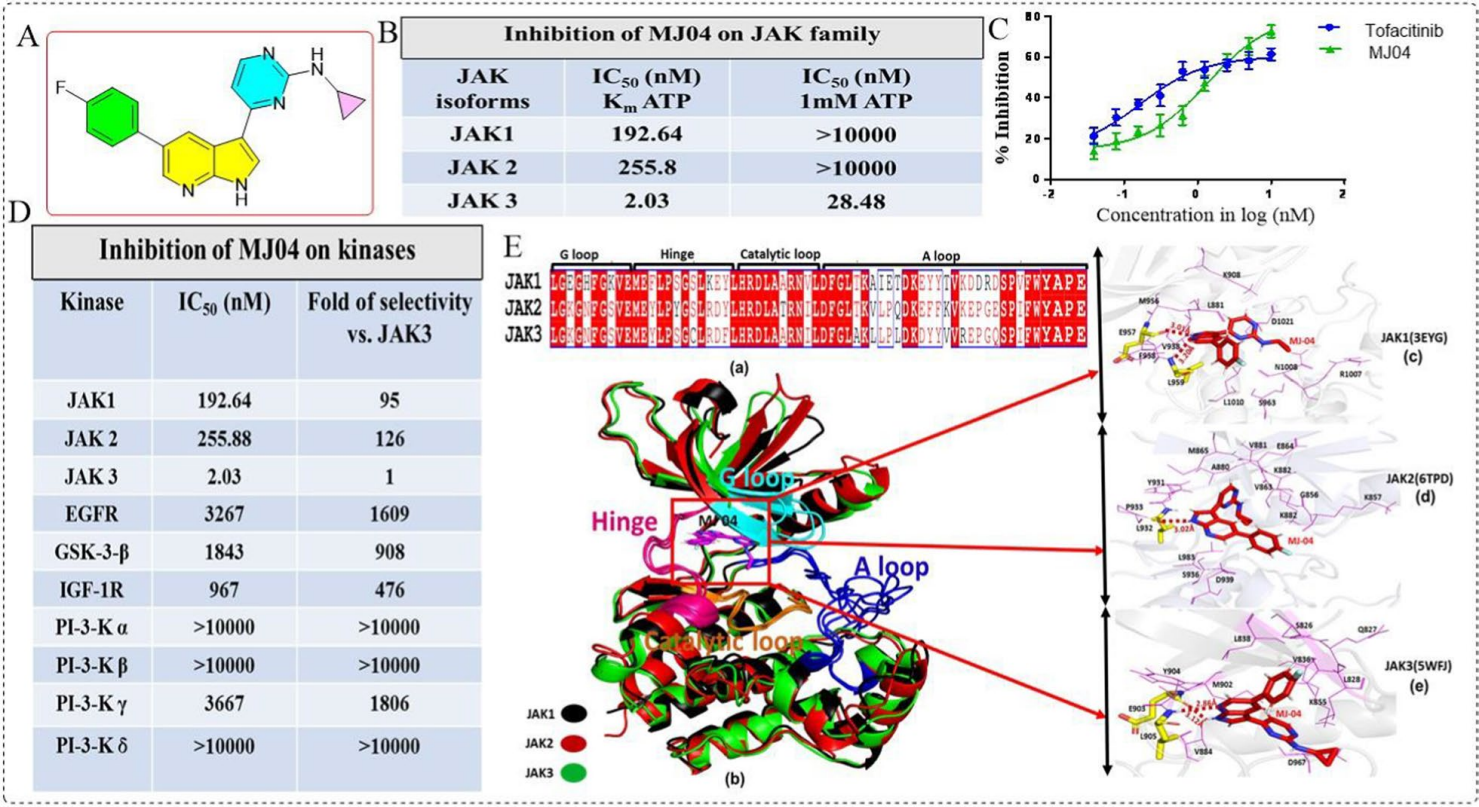
Identification of MJ04 as JAK3 inhibitor and its biological characteristics. **A** The chemical structure of compound MJ04. **B** Inhibitory activity and selectivity of MJ04 for JAK family kinases at Km and 1 mM ATP concentration. **C** The IC50 of MJ04 and tofacitinib inhibiting JAK3 at Km ATP concentration. **D** The inhibitory potency and selectivity of MJ04 against a panel of 10 kinases at Km ATP concentration. **E** Intermolecular interactions of MJ04 with JAK1 (3EYG), JAK2 (6TPD) and JAK3 (5WFJ) kinase domain: **E** (a) Multiple sequence alignment of JAK1, JAK2 and JAK3 with conserved regions labelled as G loop (glycine rich loop, L881-E890-JAK1, L855-E864-JAK2, L828-E837-JAK3), hinge region (M956-L968-JAK1, M929-L941-JAK2, M902-L914-JAK3) catalytic loop (H1001-L1010-JAK1, H974-L981-JAK2, H947-L956-JAK3) and A loop (activation loop, D1021-E1051-JAK1, D994-E1024-JAK2, D967E997-JAK3) indicating the conserved residues in each region. **E** (b) represents the superimposed structure of JAK1, JAK2 and JAK3 with inhibitor molecule MJ04 present in the hinge region and different regions are coloured and labelled as hinge region (hot pink), catalytic loop (orange), activation loop (blue) and glycine rich loop (cyan), the **E** (c), **E** (d) and **E** (e) is representing the enlarged view of intermolecular interactions of MJ04 with JAK1, JAK2 and JAK3 respectively with inhibitor MJ04 in stick coloured in red and heteroatoms coloured differently, hydrophobic interacting residues in wire and magenta colour and hydrogen bonding residues in stick coloured in yellow with heteroatoms differently coloured, H-bonds are represented as red coloured dotted lines with length labelled

Since the topical treatment of mouse and human skin with tofacitinib, pyrimidino pyridine scaffold, as JAK3 inhibitor was shown to promote hair growth, we conducted comprehensive screening of a small library of 3-pyrimidinylazaindole-based series of compounds for JAK3 activity. Initially, these compounds were screened at 10.0 μM concentration, and compounds that showed > 60% inhibition were further screened at 1.0 μM concentration. After several rounds of screening, ten compounds were seen to show dose-dependent response to JAK3 inhibition in cell-free assays (Additional file 1: S1-Table 1), and MJ04 was identified as the most potent ATP competitive inhibitor of JAK3 with an IC_50_ value of 2.03 nM in the presence of 2.5 µM ATP (K_m_) and IC_50_ value of 28.48 nM at 1 mM ATP concentration (Fig. 1A–C, Additional file 1: Fig. S1A). Pertinently, MJ04 showed highest selectivity for JAK3 inhibition when compared to JAK isoforms (JAK1 and 2) and a panel of other kinases which include IGF1R, GSK3β, EGFR, p110α, p110β, p110γ and p110δ (Fig. 1D). Docking simulation revealed that MJ04 binds tightly within the ATP binding region of the JAK3 through hydrogen bonding and hydrophobic interactions. MJ04 showed binding affinities of − 9.8 kcal/mol, − 9.1 kcal/mol, and − 9.8 kcal/mol with the kinase domain of JAK1 (3EYG), JAK2 (6TPD), and JAK3 (5WFJ) respectively (Additional file 1: S1-Table 2). The MJ04 interacts with the hinge region of the kinase domain in all three JAKs. MJ04 binding to JAK1 is stabilized by strong hydrogen bonding interactions of hinge region residue E957 with pyridine N atom, and residue L959 with pyrrole NH atom in the 3-azaindole ring of MJ04 (Fig. 1E-c). MJ04 pyrrole NH atom in the 3-azaindole ring showed H-bonding interactions with hinge region residue L932 in JAK2 (Fig. 1E-d), similarly, pyrroleNH atom showed H-bonding interactions with hinge region residues E903, and pyridine N atom in the 3-azaindole ring with L905 residue in JAK3 (Fig. 1Ee). Moreover, MJ04 is stabilized and kept intact in the hinge region by various hydrophobic interactions of kinase domain residues. The JAK1 hydrophobic interacting residues with MJ04 are L881, K908, V938, S963, M956, F958, R1007, N1008, L1010 and D1021, the JAK2 hydrophobic interacting residues are G856, K857, V863, E864, M865, A880, V881, K882, Y931, P933, S936, D939 and L983 and the JAK3 hydrophobic interacting residues are S826, Q827, L828, V836, L838, K855, D867, V884, M902 and Y904 (Fig. 1Ea-e). The root means square deviations (RMSDs) and root mean square fluctuations (RMSFs) graph showed that all three JAK complexes with MJ04 are stabilized within 4 Å deviations (Additional file 1: Fig. S1B, C). The binding affinity and intermolecular hydrogen bonding and hydrophobic interactions of the hinge region and other regions of the kinase domain are prevalently more in the case of JAK3, followed by JAK1 and less in the case of JAK2 with inhibitor MJ04, as observed during in silico analysis of docking and simulations studies (Additional file 1: Fig. S1D, E). Taken together, MJ04 was demonstrated to selectively inhibit JAK3 kinase activity under the experimental conditions applied.

### Structure–activity relationship of MJ04 and its impact on JAK-STAT phosphorylation

**Fig. 2:**
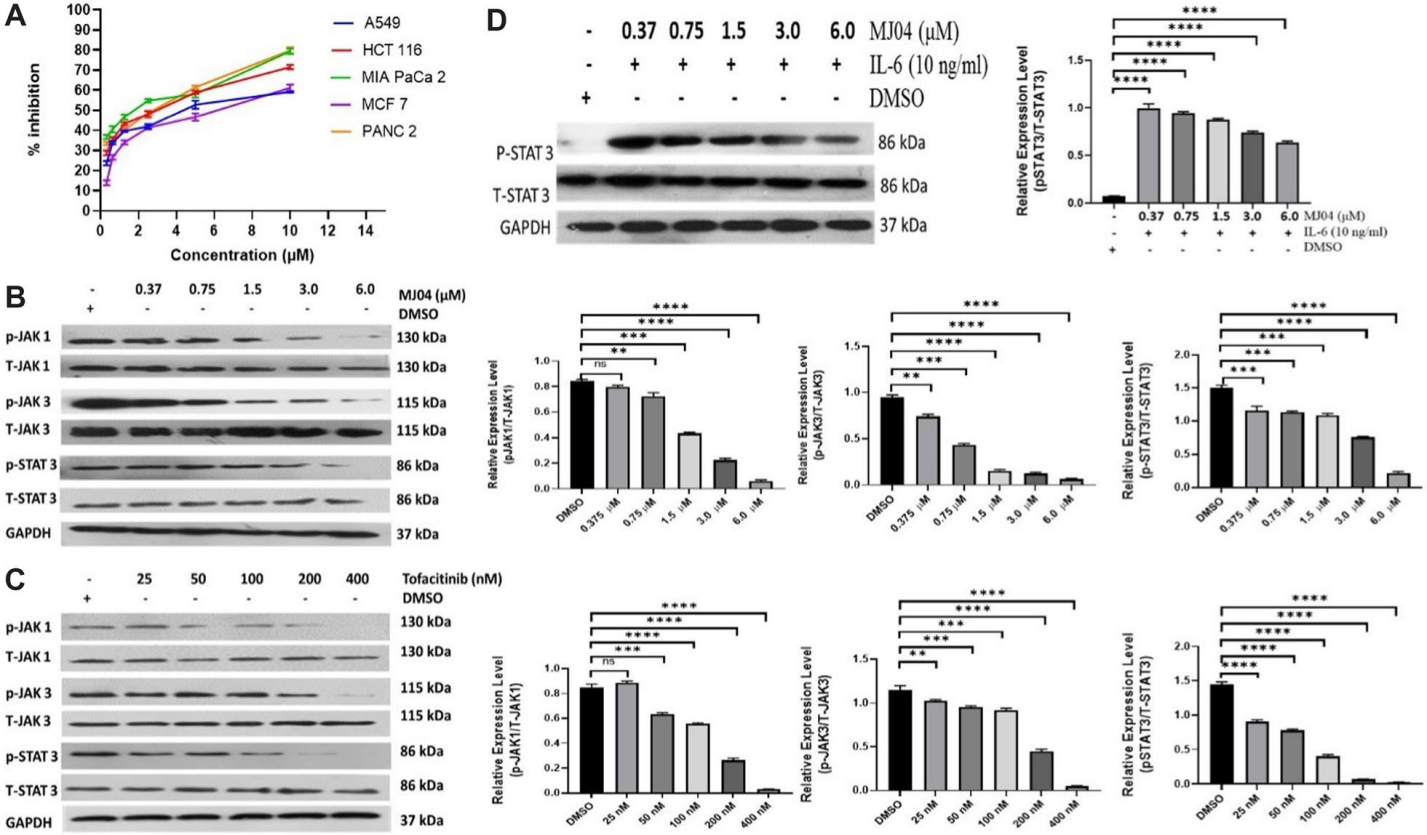
Selective Inhibition of the JAK3-Dependent Signaling Pathway by MJ04. **A** Assessment of cell cytotoxicity induced by MJ04 and its determination of IC50 values across different cancer cell lines. **B** Examination of the impact of MJ04 on the phosphorylation status of JAK1, JAK3, and STAT3. **C** Evaluation of the effect of Tofacitinib on the phosphorylation of JAK1, JAK3, and STAT3. **D** Assessment of MJ04’s impact on STAT3 phosphorylation in the presence of IL-6 of A549. GAPDH served as the loading control. Densitometry analysis results for each immunoblot are depicted in the accompanying graph to the right. All data analysis was performed using MS Excel, GraphPad Prism-V8.0 Software, and Image-J. Statistical significance was determined through one-way analysis of variance (ANOVA). Data points are presented as mean ± SD, and significance values are denoted as *P < 0.05, **P < 0.005, ***P < 0.0005, and ****P < 0.00005."

In humans, the JAK family comprises four isoforms: JAK1, JAK2, JAK3, and Tyk2, which are selectively associated with the cytoplasmic domains of membrane-bound receptors thus allowing trans-phosphorylation and subsequent activation of JAKs. These activated JAKs, in turn, phosphorylate the receptors, and STAT proteins. There are seven isoforms of STATs reported in humans, which include STAT1, STAT2, STAT3, STAT4, STAT5a, STAT5b, and STAT6. Upon phosphorylation, STATs dimerize and translocate into the nucleus to activate or repress transcription of target genes. To evaluate the anti-proliferative abilities of 3-pyrimidinylazaindole-based series of compounds, previously reported by us as protein kinase inhibitors [31], we synthesized their analogues and performed MTT assays in five cancer cell lines, which include A549, HCT-116, Mia PaCa-2, MCF-7, Panc-2, and one normal cell line (HEK293) (Fig. 2A and Additional file 1: Fig. S2A–E). The 3-pyrimidinylazaindole has three rings: A, B, and C. While the molecules MJ07 and MJ08, with substitution on ring A only, are less active; the molecules MJ01, MJ02, MJ03, MJ04, MJ05, MJ06, MJ09, and MJ10, with substitutions on ring A and C, show significant inhibitory activity against JAK3 in kinase assays and in MTT assays (Additional file 1: S1-Table 1, S2-Table 1). To test the impact of MJ04 on JAK and STAT phosphorylation, we incubated A549 cells with increasing concentrations of MJ04 and observed a significant reduction in the phosphorylation state of JAK1 and JAK3 (Fig. 2B) [32, 33]. Pertinently, the phosphorylation status of STAT3 at Tyr705 also showed a significant decrease upon treatment with MJ04 in a dose-dependent manner. Interestingly, no detectable JAK2, Tyk2, STAT1, and STAT5 levels were seen in A549 cells. Also, treatment of A549 cells with Tofacitinib, used as a positive control, showed a significant decrease in the phosphorylation of JAK3, JAK1, and STAT3 in a dose-dependent manner (Fig. 2C). Similarly, we stimulated A549 cells with IL-6, measured STAT3 levels, and observed a significant decrease in STAT3 phosphorylation (Fig. 2D). These results suggest that MJ04 is involved in inhibiting STAT3 phosphorylation through JAK/STAT3 pathway.

### MJ04 is involved in promoting hair growth in C57/B6 mice

**Fig. 3:**
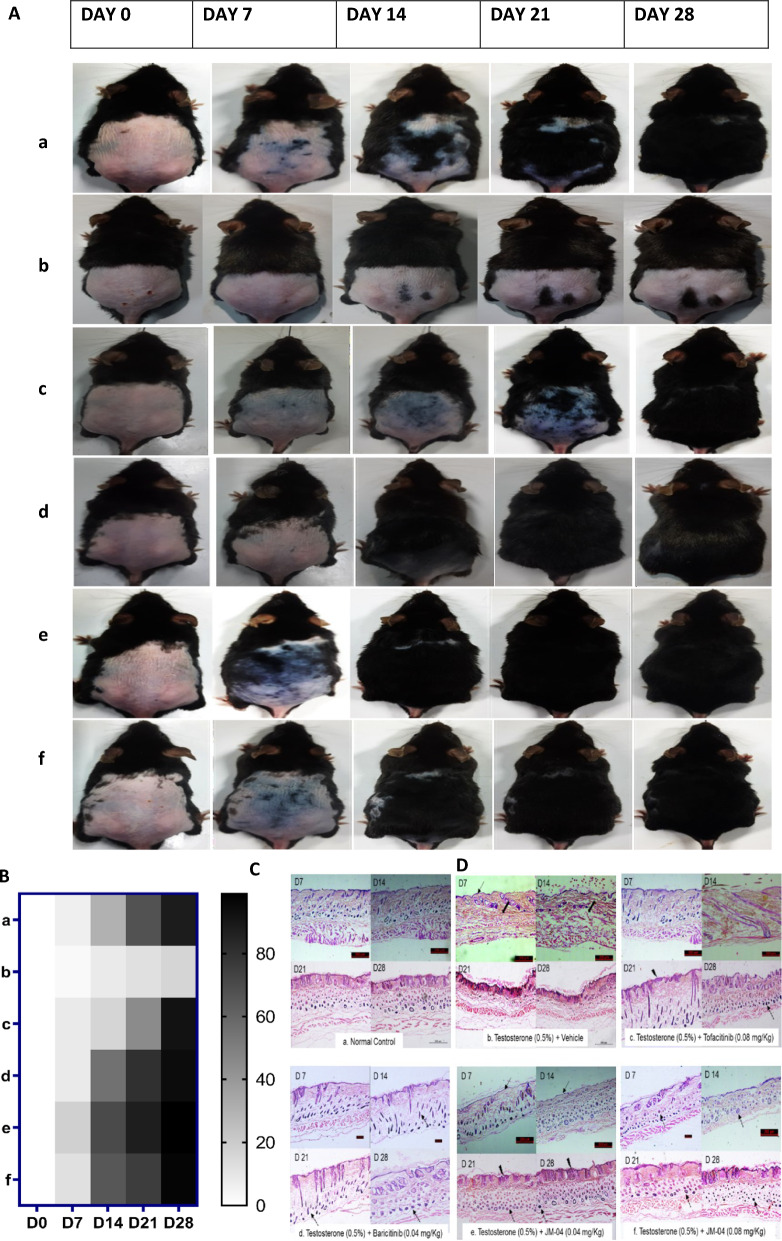
Effect of MJ04 on hair regrowth in the DHT induced AGA mice model. The shaved back skin of C57BL/6J mice were daily treated with 0.5% testosterone for 1 h prior to topical application of various concentration of tofacitinib, MJ04 and baricitinib for 28 days. **A** MJ04 administration induced the telogen-anagen transition. Digital photographs were capture from a representative area using Nikon digital camera (n = 8 mice), **B** Qualitative analysis:—The quantification of anagen initiation was assessed by observing changes in the dorsal skin colour of C57BL/6 mice, a phenomenon closely linked to the onset of anagen phase. Anagen quantification was carried out by employing threshold analysis on dorsal skin colouration using ImageJ. The darkening of the skin, attributed to melangenesis, strongly correlates with progression of anagen. This analysis is visually depicted as a heatmap. Here a = Control; b = Testosterone treated; c = Tofacitinib treated (0.08mg/kg), d = Baricitinib treated (0.04mg/kg), e = MJ04 treated (0.04mg/kg) and f = MJ04 treated (0.08mg/kg), **C** Dorsal skin samples from C57BL/6J mice were shaved and collected for histological and molecular analyses on Days 7, 14, 21, and 28. The histopathological examination of the samples were executed. n = 8 mice per group H&E stained skin section of C57BL/6J mice (× 200 magnification). a. Normal histoarchitecture with hair follicles. b. Epidermal thinning  with very tiny hair follicles . c. Minimal epidermal thinning with few hair follicles on Days 7 and 14 and increased hair follicles on Days 21 and 28 . d. Minimal degree of epidermal thickening with increased distinct hair follicles  in the dermal and hypodermal regions. e. Minimal degree of epidermal atrophy  with hair follicles on Days 7 and 14 and minimal epidermal thickening  with increased hair follicles  on Days 21 and 28. f. Normal epidermis with increased hair follicles  on Days 14 and 28. **D** Mean Weekly Body Weight: Monitoring of mean weekly body weight of DHT induced AGA mice model throughout the evaluation of MJ04’s impact on hair regrowth. Here, the groups are denoted as follows a = Control; b = Testosterone treated; c = Tofacitinib treated (0.08mg/kg), d = Baricitinib treated (0.04mg/kg), e = MJ04 treated (0.04mg/kg) and f = MJ04 treated (0.08mg/kg). The body weight of the induced AGA mice model was recorded on days 1, 7, 14, 21, and 28

In order to investigate the in vivo efficacy of MJ04, we used DHT-induced AGA mice model [22, 23, 34]. This is an excellent model system for studying the hair cycle because, unlike in humans, short hair cycle in mice is completed in about 3 weeks only, and the first two cycles of hair growth are synchronized in mice [35]. The C57/B6 mice in the early telogen phase (7 weeks) were shaved and topically treated either with vehicle control (negative control), tofacitinib, and baricitinib or with various concentrations of MJ04 in the presence of testosterone once a day for 28 days. While the vehicle-treated mice remained in the telogen phase for the entire period of this experiment, mice treated with tofacitinib, baricitinib, or MJ04 showed rapid entry into the anagen phase within seven days of treatment (Fig. 3A, B, Additional file 1: Fig. S3A–K). Pertinently, treatment of mice with 0.04 mg/Kg MJ04 consistently yielded significantly higher and homogeneous hair growth when compared to controls and tofacitinib-treated mice. Meanwhile, stability assessment of MJ04 was conducted and the compound was found stable in the formulation used for animal experiments (Additional file 1: Fig. S3L). To further assess the hair growth-promoting activity of MJ04, we conducted a histopathological analysis of skin samples taken on days 7, 14, 21, and 28. While the hairs in vehicle control group were in the early anagen phase (hair bulb in the dermis), the hair follicles in the tofacitinib, baricitinib, or MJ04 treated groups were at least in the anagen IIIc-IV phase, showing maximal size of hair bulb, hair follicles deep in the subcutis, and newly formed hair shaft reaching the level just below the sebaceous gland (Fig. 3C). To further demonstrate the robustness of sustained hair growth promotion upon MJ04 treatment, we monitored various other parameters like skin darkening (Fig. 3B), physical appearance, body weight (Fig. 3D), behaviour, food intake, and each mouse was closely monitored during this period, and blood samples were collected at day 21 and 28 to examine their biochemical and pharmacological parameters (Additional file 1: S3-Table 1-3). Remarkably, around 90% of mice treated with tofacitinib or MJ04 displayed intense skin darkening followed by hair growth within 10 days of treatment compared to the vehicle control group. The experiments were independently replicated, and reproducible results were consistently observed. Collectively, these data suggest that MJ04 treatment of mice causes rapid onset of hair growth.

### MJ04 stimulates the early onset of hair growth in athymic nude mice (NU/J *Foxn1*^*nu*^) and growth of human hair follicles under ex-vivo conditions

**Fig. 4:**
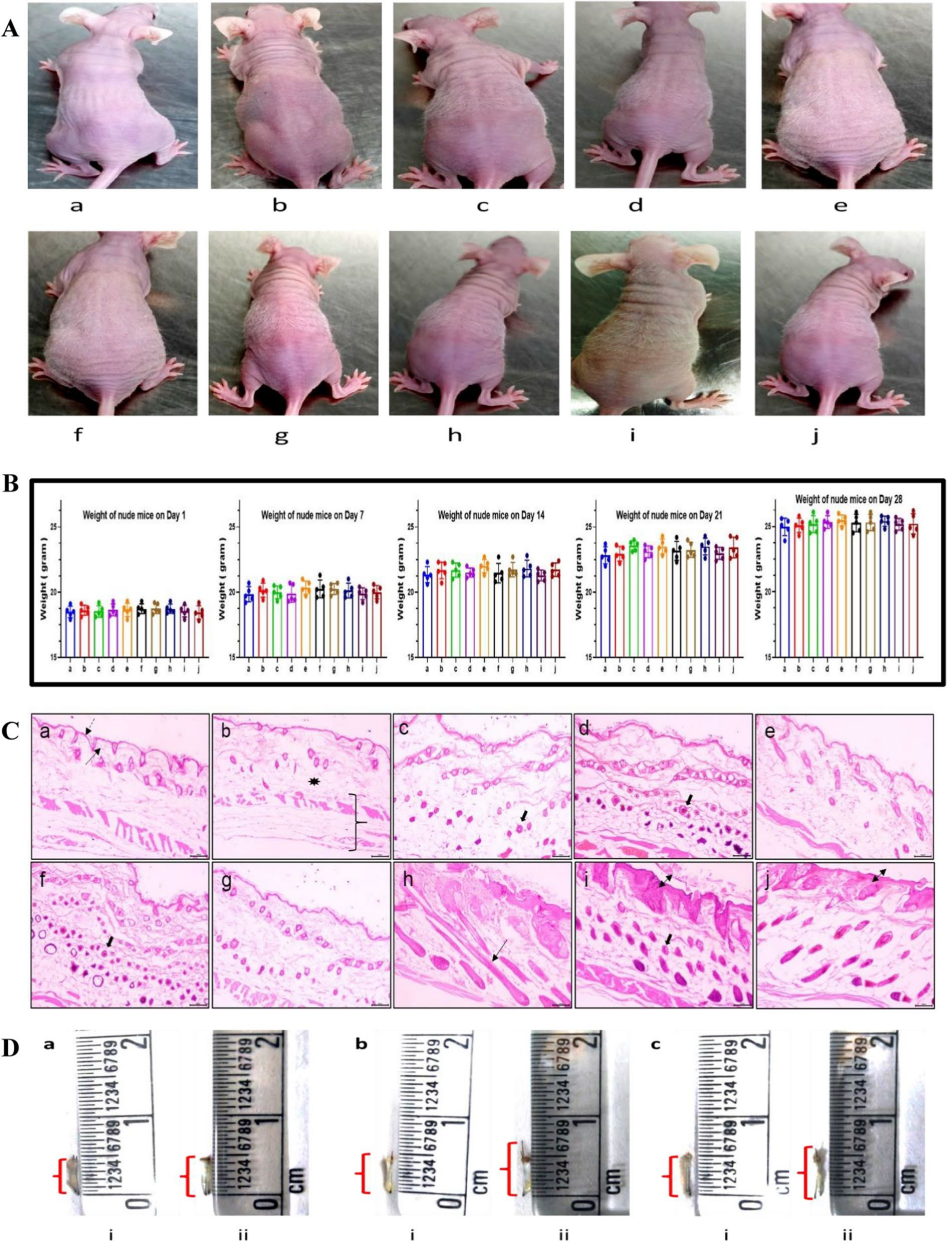
MJ04 induces early onset in anagen development, hair cycle progression in nude mice, and growth of human hair follicles under ex-vivo conditions. **A** The treatment with MJ04 resulted in the transition from telogen-anagen, as evidenced by the transformation of initially hairless dorsal skin to white-haired skin. Digital photographs were captured from the representative areas using a Nikon digital camera (n = 5 mice). a- Control, b-Vehicle control, cTofacitinib (0.8mg/Kg), d-Tofacitinib (0.08mg/kg). e-MJ04 (0.08mg/Kg), f- MJ04 (0.04mg/kg), g- MJ04 (0.016mg/kg), h- Baricitinib (0.1mg/kg), i- Baricitinib (0.04 mg/kg), j- Baricitinib- (0.02 mg/Kg). **B** Mean Weekly Body Weight: Monitoring of mean weekly body weight of athymic nude mice (NU/J Foxn1nu) mice model throughout the evaluation of MJ04's impact on hair regrowth. Here a- Control, b-Vehicle control, c-Tofacitinib (0.8mg/Kg), d-Tofacitinib (0.08mg/kg). e-MJ04 (0.08mg/Kg), f- MJ04 (0.04mg/kg), g- MJ04 (0.016mg/kg), h- Baricitinib (0.1mg/kg), i- Baricitinib (0.04 mg/kg), j- Baricitinib- (0.02 mg/Kg). The body weight of the nude mice was recorded on days 1, 7, 14, 21, and 28. **C** The skin samples collected from athymic nude mice (NU/J Foxn1nu) were preserved in 10% neutral buffered formalin and subsequently subjected to tissue sectioning. Skin sections (10 µm) of nude mice were stained with haematoxylin and eosin (H&E) and viewed under 10× and 40× objectives of the light microscope (Magnus, Mx21i). The histopathological examination of the samples was performed, and the hair follicles in the skin sections were graded on a scale ranging from 0 to 4 (++++). H&E stained the skin section of nude mice (×100 magnification). a and b (control & vehicle control): Normal histoarchitecture with the intact epidermis (, dermis , hypodermis , panniculus carnosus, and adventitia . c and d (Tofacitinib citrate—0.08 and 0.8 mg/kg): Mild epidermal thickening with the increased number of hair follicles [] (++++). e (MJ04—0.08 mg/kg): Minimal epidermal thickening with moderate hair follicle (++). f (MJ04—0.04 mg/kg): Mild epidermal thinning with increased hair follicles in the dermis and hypodermis (++++). g (MJ04—0.016 mg/kg): Mild epidermal thickening with increased hair follicles (+++). h (Baricitinib–0.1 mg/kg): Increased and morphologically distinct hair follicles  in the dermis and hypodermis regions (+++). i and j (Baricitinib—0.04 and 0.02 mg/kg): Moderate degree of epidermal thickening  with more hair follicles in the dermis and hypodermis regions (++++), and **D** MJ04 promotes the growth of human hair follicles under ex-vivo conditions. (a) Control group: (i) Basal length, (ii) Length posttreatment, (b) Experimental group treated with 100 μl of MJ04 (0.50 μg/μl): (i) Basal length, (ii) Length post-experiment, and (c) Experimental group treated with 100 μl of MJ04 (0.25 μg/μl): (i) Basal length, (ii) Length post-treatment. The red bracket indicates the length of the human hair follicle

Athymic nude mice are not totally hairless, they rather develop same number of hair bulbs in the hypodermis as normal mice [36]. These mice exhibit a narrow band of thin hair from head to tail in a 20-day hair cycle. Since the keratinization process of hair is impaired due to *FOXN1* gene mutation, the hair shaft is short, bent, crippled, breaks, or hardly emerges from other parts of the body [37]. Baricitinib, tofacitinib, and MJ04 were topically applied to 5 mice (each group) for two hair cycles, and 5 mice in the control group received vehicle only. Systematic monitoring of treated and untreated mice through two full growth cycles was done by photographing each animal. Significant differences in hair growth were observed in the treated mice as compared to the control mice. Early onset of hair was observed at day 12 in MJ04, tofacitinib, and baricitinib treated mice when compared to control mice (Fig. 4A, Additional file 1: Fig. S4i–x). During this period, physical appearance, behaviour, food intake, and body weight of each mouse was closely monitored, and no changes were observed in the treated and control groups (Fig. 4B, Additional file 1: S4-Table 1). Histopathological analysis of skin samples taken on day 28 showed distinct changes in the dermis and epidermis of mice, with the significantly increased number of hair follicles in the treated mice (Fig. 4C). We also analyzed the effect of JAK inhibition on hair shaft elongation using the human hair follicle organ culture model. Individual hair follicles micro-dissected from the scalp tissue of volunteers were cultured in the presence of MJ04 or vehicle (DMSO) for 20 days. Treatment of these follicles with MJ04 significantly increased the length of hair shafts when compared to the vehicle and untreated follicles, thereby indicating a positive effect of MJ04 on hair shaft elongation (Fig. 4D). The data suggests the growth-promoting effect of JAK3 specific inhibitors in human hair follicle organ culture. Together, this data implies that inhibition of JAK-STAT signaling promotes anagen re-entry and allows the progression of the normal hair cycle.

### JAK3 inhibitor MJ04 dampens the naive T-cell differentiation into proinflammatory phenotypes and inhibits the proinflammatory cytokines in LPS-induced macrophages

**Fig. 5:**
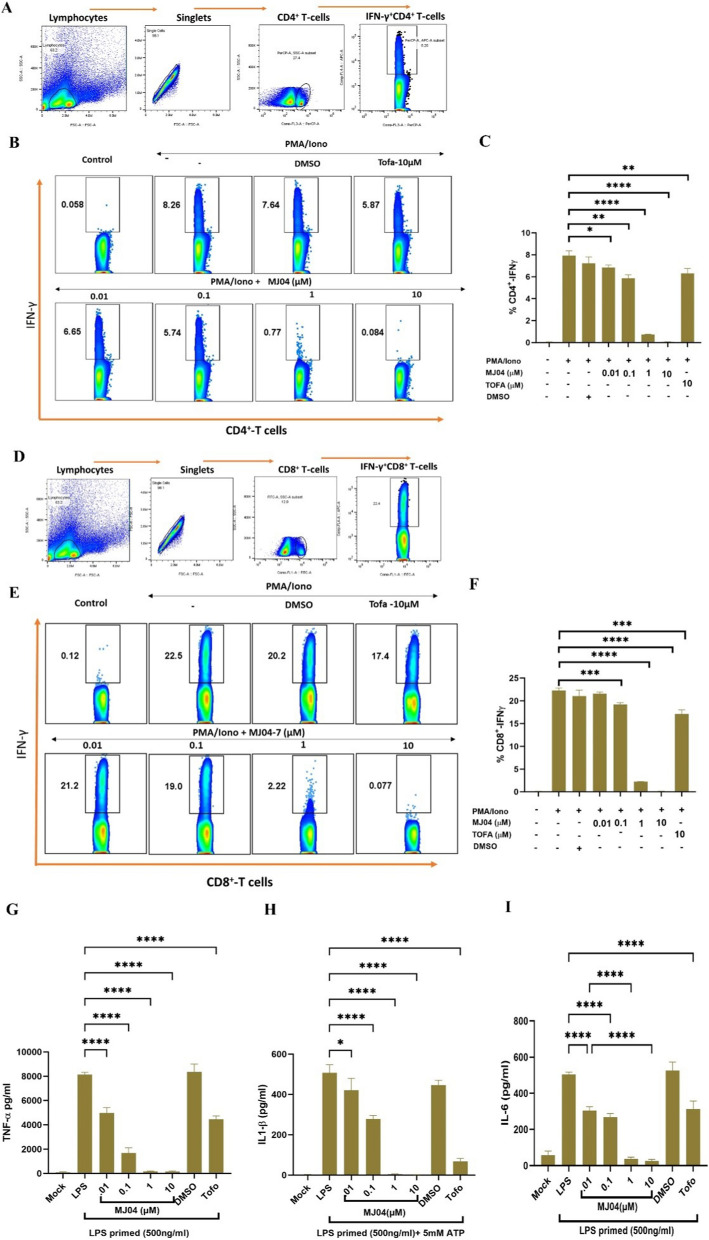
MJ04 inhibits the differentiation of naïve T-cell into Th1 and Tc1 subsets, also inhibits the level of proinflammatory cytokines in LPS primed macrophages. Splenoctyes from healthy mice were used as a source of T-cells, Spelnocytes were stimulated with anti-CD3/C28 in the presence and absence of MJ04 (0.01, 0.1, 1 and 10 µM) for 96 h and before harvesting these cells were further stimulated with PMA/Ionomycin for additional 4 h. **A**–**D** is the gating strategy to deduce the cell of interest. The expression of IFN-γ in the CD4^+^(**C**,** D**) and CD8^+^ (**E**, **F**) T-cells were analyzed by flowcytometry. In another set of experiments, mouse macrophage (J774a.1) were induced with LPS and then treated with different concentration of MJ04 and Tofacitinib. The level of pro inflammatory cytokines (TNF-α, IL1-β and IL-6) were measured by sandwich ELISA from LPS induced macrophages (**G**–**I**). Each data point is representative of n = 3). Data points are represented as mean ± SEM and value of significance represents *P < 0.05, **P < 0.005, ***P < 0.0005 and ****P < 0.00005

The differentiation of naïve CD4^+^ and CD8^+^ T-cells into functionally diverse subsets, such as Th1 and Tc1, is an important phenomenon to mount effective immunity against pathogens. However, dysregulation of these immunophenotypes triggers autoimmunity and other inflammatory diseases [38, 39]. The differentiation process is primarily regulated by γc-dependent cytokines such as IL-2 and IL-21, which operates through the JAK-STAT signalling pathway [40]. Therefore, we evaluated the potential of MJ04 in restricting the differentiation of naïve T-cells into Th1 and Tc1 proinflammatory subsets. In brief, splenocytes from C57BL/6 mice were isolated and stimulated with anti-CD3/CD28 antibody in the presence or absence of MJ04 and tofacitinib for 96 h. Further, we analyzed the IFN-γ-producing CD4 + T-cells and CD8 + T-cells which are immunophenotypes of Th1 and Tc1 subsets, respectively, that play an important role in the autoimmune and other inflammatory diseases. Results clearly demonstrate that MJ04 inhibits the differentiation of naïve T cells under the polarizing condition into Th1 subsets in a dose-dependent manner by abolishing the production of IFN-γ cytokines, therefore attenuating the polarizing signals (Fig. 5A, B and Additional file 1: Fig. S5) and Tc1 (Fig. 5C and D). Furthermore, we performed the innate inflammatory responses in the LPS-primed murine macrophages (J774a.1) upon treatment of MJ04. Data clearly demonstrates that MJ04 dampens the level of proinflammatory cytokines, i.e., TNF-α, IL1-β, and IL-6, in a dose-dependent manner in the LPS-primed macrophages (Fig. 5G–I). In conclusion, JAK3 inhibitor MJ04 effectively hinders the polarization of naïve T-cells into the proinflammatory immunophenotypes and also attenuates the myeloid inflammatory cytokine milieu, consequently, it has the potential to restore the immune homeostasis and manage autoimmune inflammatory conditions.

### Safety, dermal toxicity, and pharmacokinetic properties of MJ04

**Fig. 6:**
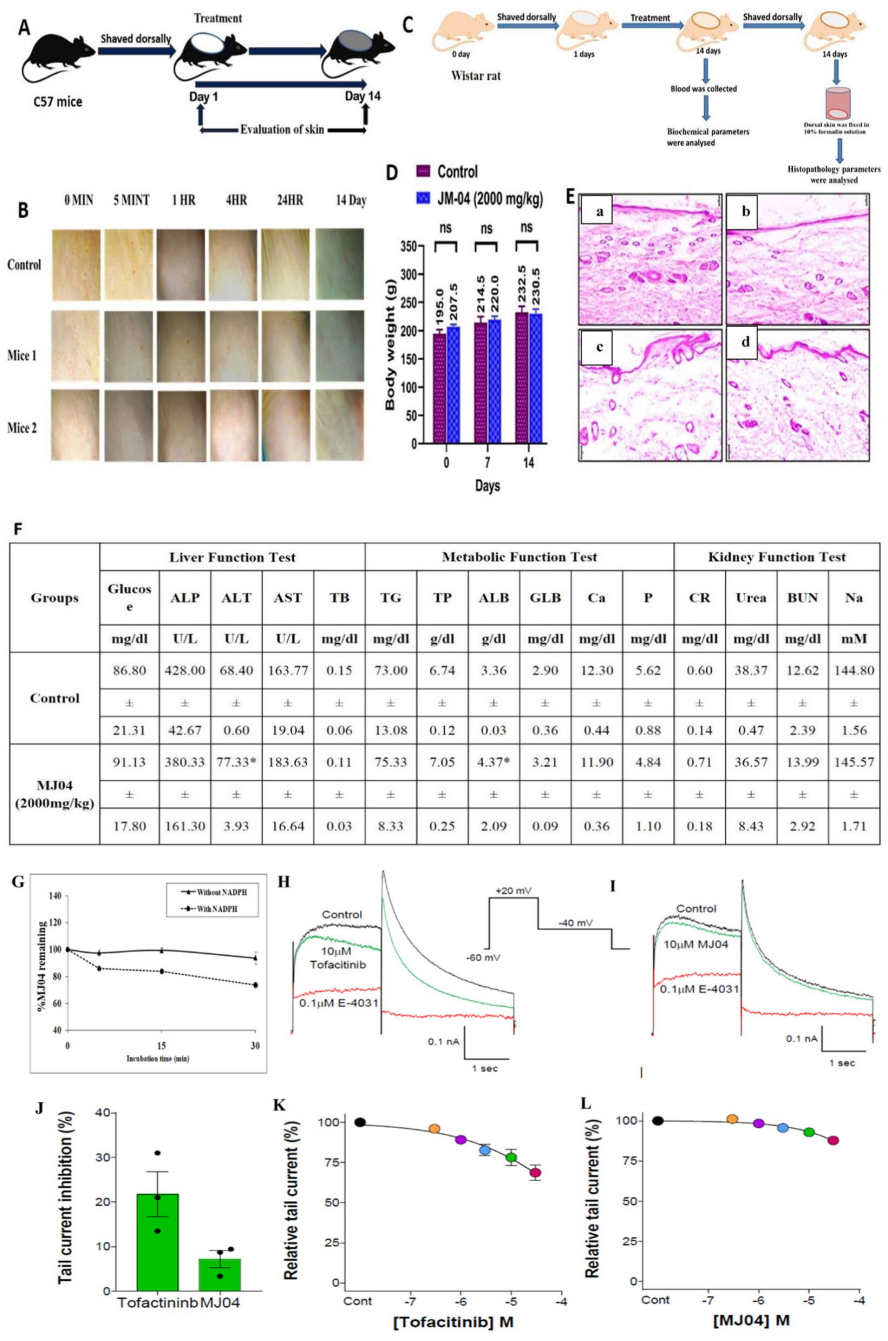
Preclinical safety and toxicity profile of MJ04. **A** Experimental Overview: The dorsal skin of C57BL/6J mice was shaved, and subsequent treatments were applied to the dorsal skin surface on the following day. **B** Acute Dermal Toxicity Assessment: Signal dose acute dermal toxicity tests were conducted in C57BL/6 mice. **C** Experimental overview of Median Lethal Dose (LD_50_) Determination: Acute dermal toxicity tests were conducted in Wistar rats. Limited testing included a single dose at 2 g/kg body weight. **D** Mean Weekly Body Weight: Monitoring of mean weekly body weight during the acute dermal toxicity assessment of MJ04. **E** Histopathological Analysis of dorsal skin tissue: Haematoxylin and eosin images depict the dorsal skin of mice (n = 5 mice per group). The histopathology images are presented at 10× magnification and show the skin of the control (a and b) and MJ04 treated group (2000 mg/kg) (c and d). **F** Mean serum biochemistry of acute dermal toxicity of MJ04. The values are expressed as mean ± SD (n = 3); *Significant difference from the control group at P < 0.05; b.w.: Body weight; GLU: Glucose, ALP: Alkaline Phosphatase, ALT: Alanine Transaminase, AST: Aspartate Amino Transferase, TB: Total Bilirubin, TG: Triglycerides, TP: Total Protein, ALB: Albumin, GLB: Globulin, CR: Creatinine, BUN: Blood Urea Nitrogen, Ca: Calcium, P: Phosphorus, Na: Sodium; No significant difference in the means of treatment groups and control group at 0.05 level. **G** Represents the metabolic stability of MJ04 in human liver microsomes. **H**–**L** shows the effect of MJ04 on hERG channel currents. Representative current traces show the effect of control (0.1% DMSO, black traces), 0.1 µM E-4031 (red traces), 10 µM Tofacitinib (green traces, **H**), 10 µM MJ04 (green traces, **I**). The recording protocol employed is shown in inset. **J** Mean tail current % inhibition for each drug is plotted. **K**, and **L** shows the summary dose response for 5 concentrations of the Tofacitinib (**K**) and MJ04 (**L**) ranging from 0.3 µm to 30 µM. Data points are the mean ± SEM (n = 3)

To further estimate the safety, metabolism, and pharmacokinetic properties of MJ04, we carried out skin irritation test in mice and dermal toxicity analysis in rats via its topical application. C57BL/6 J mice were treated with a single dose of MJ04 (0.08 mg/kg) and monitored daily for 14 days. Notably, no skin irritation/corrosion, mortality or clinical signs of toxicity were observed upon treatment of mice (Fig. 6A, B). The acute dermal toxicity of MJ04 showed no mortality and clinical signs of toxicity at 2000 mg/kg body weight. (Fig. 6C–E). MJ04-treated animals did not show any adverse effect on biochemical parameters (liver, kidney function test, and lipid profile) and body weight of the animals (Fig. 6D–F). In addition, no gross histopathological changes were observed in the liver, kidney, lungs, and brain upon treatment with MJ04 (2 g/kg) when compared to control rats (Additional file 1: Fig. S6A). Based on the findings, the LD_50_ of MJ04 was found to be > 2000 mg/kg body weight under tested conditions and shall be classified under Category-5 as per the Globally Harmonized Classification System (GHS) for Chemical Substances and Mixtures. To evaluate the pharmacokinetic properties of MJ04, a topical dose of MJ04 at 30 mg/kg showed a reasonable pharmacokinetic profile with a C_max_ of 0.48 µM and T_max_ of 1 h (Additional file 1: Fig. S6B). The compound remained in plasma for an adequate time, and during metabolic stability analysis, a similar line of time was observed in human liver microsomes (Fig. 6G). Evaluation of cardiac toxicity has become a primary regulatory requirement of early pre-clinical drug development. Drug induced blockade of cardiac hERG channels is considered as one of the primary causes of cardiotoxicity, therefore, we evaluated the effects of MJ04 and compared it to Tofacitinib using the conventional whole cell patch clamp technique. In this assay, HEK293 cells stably expressing hERG channels were treated with vehicle (0.1% DMSO), E-4031 (0.1 μM), Tofacitinib (0.3–30 μM) and MJ04 (0.3–30 μM). Figure 6H, I illustrates the representative current traces before (control, black traces) and after the application of 10 μM Tofacitinib and MJ04 (green traces) and 0.1 μM E-4031 (red traces). Tofacitinib significantly reduced the hERG outward tail currents by 22 ± 5% (p < 0.01), whereas the MJ04 application did not produce any significant reduction in the current (7.1 ± 2%) (Fig. 6J). E-4031 treatment reduced the hERG tail current by approximately 95% as reported earlier [41]. The concentration–response curves (ranging from 0.3 to 30 μM) for Tofacitinib and MJ04 are presented in panels Fig. 6K, L**,** respectively. The inhibitory effects of MJ04 were ~ 2.5 fold less than that of Tofacitinib at the highest concentration tested (30 μM). Furthermore, SwissADME and ADMETlab 2.0 web servers were utilized to calculate the pharmacokinetic characteristics and toxicity of MJ04 in humans. The results indicated that MJ04 adhered to the Lipinski Rule and did not possess any cytochrome P450 CYP2D6 enzyme inhibitory activity (Additional file 1: Table S6). Taken together, MJ04 demonstrated a favorable safety profile, drug-like pharmacokinetic properties, and metabolic stability under in-vivo and in-vitro conditions.

## Discussion

**Fig. 7:**
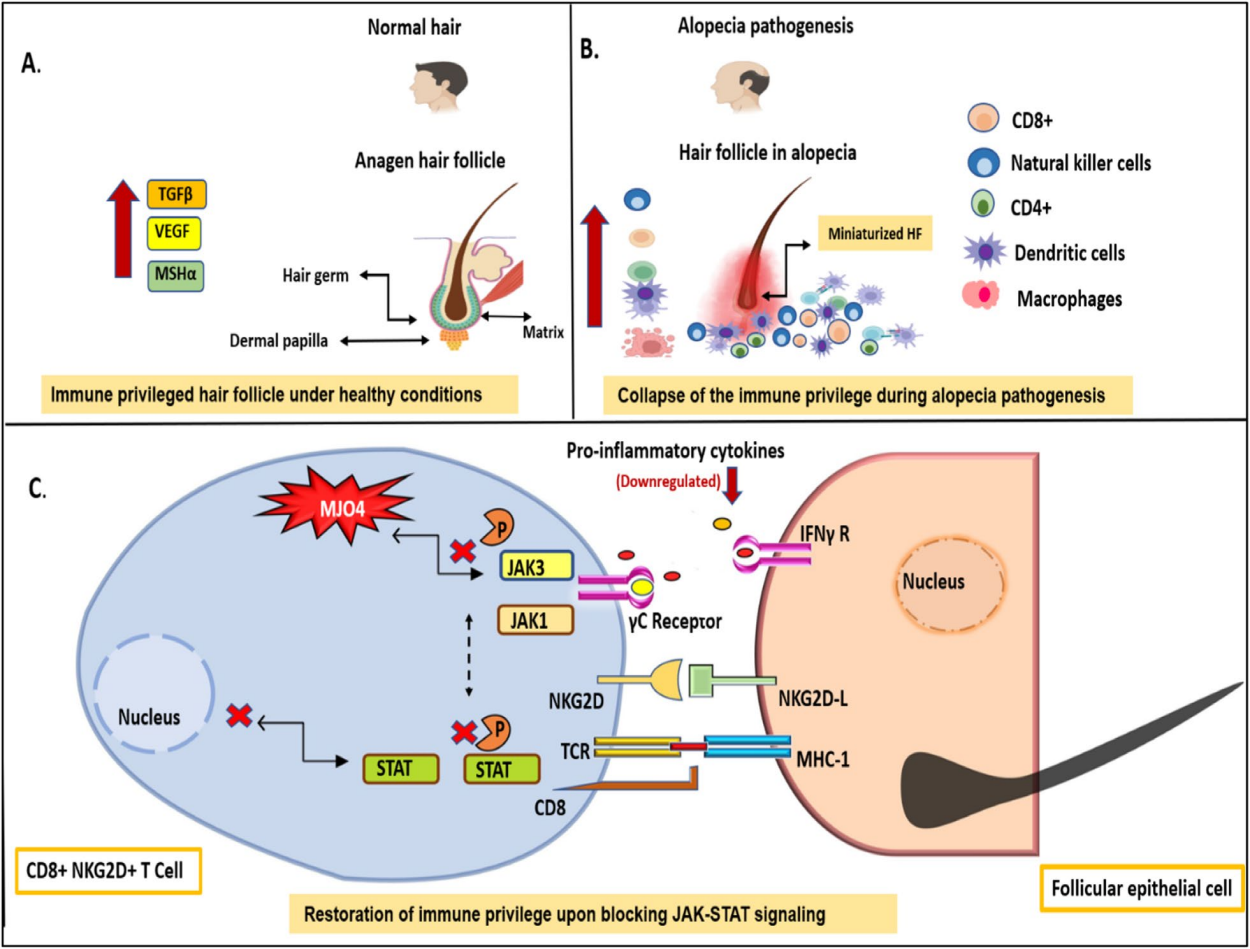
The model shows the fate of hair follicles under alopecia pathogenesis and restoration of growth of hair follicles upon blocking the production of pro-inflammatory cytokines with MJ04 as JAK3 inhibitor. **A** Shows the healthy hair follicle in the anagen phase of the growth cycle. **B** Shows the disrupted microenvironment of hair follicles due to infiltration of proinflammatory cytokines and immune cells during alopecia pathogenesis. **C** Blocking JAK-STAT signaling in immune cells inhibits the production of pro-inflammatory cytokines that restore back homeostasis around hair follicles allowing them to enter into a new growth cycle

JAK-STAT pathway is an evolutionarily conserved signaling pathway involved in transmitting extracellular signals from cell-membrane receptors to the nucleus to regulate cell growth, survival, differentiation, proliferation, and apoptosis [42]. The extracellular signaling pathways include a wide range of cytokines, interferons, growth factors, and related molecules that upon binding to type I and II cytokine receptors cause dimerization of these receptors and result in receptor activation [43]. Upon receptor activation, JAK proteins associate with the juxtamembrane regions of these receptors to initiate transphosphorylation of JAKs, and subsequent recruitment of STAT proteins to this complex [42]. This culminates in the phosphorylation-mediated dimerization of STATs which then translocate into the nucleus to regulate transcription of myriad target genes. Among the four isoforms of JAK: JAK1, JAK2, and TYK2 are ubiquitously expressed in all cell types, and JAK3 is confined only to hematopoietic cells where it is involved in mediating activity of several cytokine (γC family of cytokines) [44]. Understanding the crucial role of JAK3 signaling in the regulation of cytokine-mediated immune response, it becomes imperative to project its involvement in autoimmune-associated disorders. Hair loss disorder, commonly called alopecia areata is an autoimmune disorder in which hair follicle immune privilege is disrupted by constitutive amplification of proinflammatory cytokines in the HF microenvironment. Thus, JAK3 has become an ideal target for the treatment of inflammatory and autoimmune diseases including hair loss [45]. In 2015, Harel et al*.* reported that JAK-STAT signaling plays a critical role in promoting hair regeneration in mice. Ruxolitinib and tofacitinib, as JAK3 inhibitors, were shown to block this pathway and cause hair follicles, that have lost the ability to divide, to wake up and enter into a new growth phase [11]. In subsequent studies, the efficacy of tofacitinib in the treatment of hair loss (alopecia areata) was demonstrated in many clinical studies. Although tofacitinib is described as a JAK3-specific inhibitor, it also inhibits JAK1 and JAK2 in biochemical and cellular assays [46]. Therefore, in the pursuit of isoform selectivity, several JAK3 selective inhibitors have been reported for the treatment of rheumatoid arthritis, psoriasis, etc. [47]. Keeping in view the critical nature of JAK-STAT signaling in the regulation of hair growth and the fact that multiple drugs would come to market in coming years, we initiated the discovery and development of JAK3 selective inhibitor(s) with properties suitable for their preclinical assessment as well as for their subsequent advancement to human clinical use as hair growth promoters. We synthesized a series of 3-pyrimidinylazaindole based analogues and identified MJ04 as the most potent JAK3 inhibitor with an IC_50_ value of 2.03 nM. In biochemical assays, MJ04 demonstrated higher selectivity for JAK3 over other JAK isoforms, with no significant inhibition on a panel of 10 different kinases assessed in this study. This isoform selectivity of MJ04 is of paramount importance to avoid side effects associated with pan-inhibition of the other JAK isoforms [48]. Furthermore, cellular effects of JAK3 inhibition were examined, and MJ04 was seen to significantly inhibit JAK3 and STAT3 phosphorylation in a dose-dependent manner in A549 cells, one of the cell lines found to be sensitive to MJ04 in in-vitro cytotoxicity assays, with a safety ratio of ∼ 26 (IC_50_ against HEK293 normal cell line versus IC_50_ in A549 cancer cell line). Since JAK3 isoform primarily regulates the activity of γc-dependent cytokines, which includes IL-2 and IL-21, that are produced by Th1 or Th17 cells, we examined the effect of MJ04 on the differentiation of naive CD4^+^ and CD8^+^ T-cells into functionally diverse subsets of Th1 and Tc1 and observed a significant decrease in the polarization of naïve T-cells into proinflammatory immunophenotypes. In addition to this, MJ04 was seen to dampen the level of proinflammatory cytokines, i.e., TNF-α, IL1-β, and IL-6 in the LPS primed macrophages, thereby reiterating its role in effectively inhibiting polarization of naïve T-cells into the proinflammatory immunophenotypes in order to reinstate the immune homeostasis and manage the autoimmune inflammatory response associated with induction of alopecia areata [49, 50]. Among several strains of mice, C57BL/6 and C3H mouse strains are most frequently used for understanding hair biology and are commonly used for hair research [51]. Although several procedures and protocols have been established using mouse models, but these models still lack in providing actual representations of disease conditions. These limitations have narrowed the efforts to develop the most suitable animal or mouse model to recapture the exact mechanism of hair grow in humans [52]. To evaluate the in vivo efficacy of MJ04 in promoting hair growth, we used a DHT-challenged AGA mouse model. In this mouse model, small molecules are examined for their impact on the reversal of DHT-induced early hair regression and hair miniaturization [22]. The C57BL/6 mice were treated with MJ04, tofacitinib and baricitinib at 7 weeks of age for 28 days, and robust and reproducible regrowth of hair was observed in treated mice when compared to vehicle control mice. Pertinently, treatment of mice with 0.04 mg/kg MJ04 consistently yielded significantly higher and homogeneous hair growth when compared to controls and tofacitinib-treated mice. Indeed, ~ 90% of mice treated with tofacitinib or MJ04 displayed intense skin darkening followed by hair growth within 10 days of treatment when compared to control mice group. MJ04 was well tolerated with no serious or adverse effects as confirmed by the analysis of biochemical and haematological parameters, and many other parameters like physical appearance, body weight, behaviour, food intake, skin sensitivity toxicity. Each mouse was closely monitored during this period, blood samples were taken at day 21 and 28 to examine their biochemical and pharmacological parameters. We also tested the impact of MJ04 on human hair follicles and observed that JAK3 inhibition via MJ04 treatment significantly increases the growth rate of hair shafts under *ex-vivo* conditions. This was further corroborated by testing impact of MJ04 on the 7-week-old nude mice in telogen phase. Systematic monitoring of treated and untreated mice through two full growth cycles was carried out. Based on results, topical administration of MJ04 caused early onset of hair growth at day 12, implying that inhibition of JAK-STAT signalling promotes early anagen re-entry in nude mice. Safety analysis, dermal toxicity and pharmacokinetic properties of MJ04 was carried out in mice and rats to determine its effect on over all physiological parameters of these animals The results show that MJ04 did not have any adverse effect on general biochemical and hematological parameters of kidney, liver and lipid profile as well as the body weight of animals. Interestingly, MJ04 treatment at a high dose of 2 g/kg body weight for two weeks did not impair the total levels of WBC, monocytes, lymphocytes, neutrophils, red blood cells, hemoglobin and hematocrit. In addition to this, no pathological changes were observed in the liver, kidney, lungs, and brain upon treatment with MJ04 (2 g/kg body weight) for two weeks when compared to control rats. In quest of early pre-clinical drug development, validation for cardiac toxicity has become a primary regulatory requirement. Thus, we evaluated the effect of MJ04 on cardiotoxicity using conventional whole cell patch clamp technique. Unlike, Tofacitinib, MJ04 displayed low probability for hERG channel binding, therefore, diminishing the chances of any undesirable cardiotoxic effect. These investigations collectively suggests that long-term administration of MJ04 is quite safe and has the potential to be developed for its eventual use as a clinical candidate for hair regeneration. In summary, we show that targeting JAK-STAT signaling pathway in alopecia inhibits the production of inflammatory cytokines which  helps in restoration of homeostasis around hair follicles necessary for their re-entry into growth phase of hair cycle (Fig. 7). Hair follicles exhibit immune privilege (IP) during anagen stage of growth cycle, however, during alopecia, infiltration of pro-inflammatory cytokines cause follicle injury and loss of immune privilege around HFs. Under alopecia pathogenesis, hair follicles present auto antigens through MHC class-1 which are recognized by TCR on CD8 + NKG2D + T cells [53]. This interaction causes release of pro-inflammatory cytokines like IL-15, INF-γ, and IL-2 etc. from the activated CD8 + cells and they further interact with follicular cells. In addition to that, IL-6 released from the HFs trigger autoantigen production, leading to the amplification of autoimmune signals in AA [54]. In response to this, JAK-STAT signaling is activated in CD8 + cells that leads to intensification of cytokines around the hair follicles, therefore, disrupting the immune privilege of hair follicles [55]. On blocking the JAK-STAT mediated pro-inflammatory cytokine production, the microenvironment around agitated hair follicles is restored. This relieves the stress around hair follicles and allows them to enter a new growth cycle.

### Supplementary Information

**Additional file 1:** **Figure S1. A** (a) IC_50_ of MJ04 Km (ATP concentration). (b) IC_50_ of MJ04 at 1mM ATP concentration and (c) IC_50_ of Tofacitinib at Km ATP concentration. **B**–**F** RMSDs, RMSFs, and intermolecular interactions of MJ04 with JAK1, JAK2, and JAK3: **B.** Root Mean Square deviations (RMSDs). **C.** root mean Square Fluctuations (RMSFs) illustrating dynamic fluctuations during simulation. **D**, **E**, and **F** represent intermolecular interactions of MJ04 with JAK1, JAK2, and JAK3, respectively. **S1. Table 1.** Inhibition activities (IC_50_, nM) of small molecules against JAK3 kinase (Cell-free assay). **S1. Table 2.** Binding affinity and H-bonding interactions of ligand molecule (MJ04) in the hinge region of the kinase domain of JAK1, JAK2, and JAK3 proteins. **S2 Table 1.** Inhibition activities (IC50, μM) of small molecules against A549, HCT-116, Mia PaCa-2, MCF-7-2and Panc-2. **Figure S2A**. Schematic representation flow regarding selection of compounds against the target. **S2B.** General scheme for the synthesis of MJ04. **S2C.** ^1^H—NMR of MJ04. **S2D.** ^19^F—NMR of MJ04.**S2E.** HRMS of MJ04. **Figure S3.** Hair regrowth induced by MJ04 in a DHT-Induced Androgenetic Alopecia (AGA) mouse model. The experiment involved the daily treatment of the shaved dorsal skin of C57BL/6J mice with 0.5% testosterone for 1 h before the topical application of different concentrations of tofacitinib, MJ04, and baricitinib for 28 days. Digital photographs were taken from the representative area using a Nikon digital camera (n = 8 mice). (i)- Control group, (ii)-Testosterone group, (iii)-Vehicle group, (iv)- Tofacitinib group (0.8mg/Kg), (v)- Tofacitinib group (0.08 mg/kg), (vi)- MJ04 group (0.08 mg/kg), (vii) MJ04 group (0.04 mg/kg), (viii) MJ04 group (0.016 mg/kg), (ix) Baricitinib group (0.1 mg/kg), (x) Baricitinib group (0.04 mg/kg), and (xi) Baricitinib group (0.02 mg/kg). **Figure S3L.** Stability analysis of the formulation used for in vivo studies. The stability study of the lead compound (MJ04) was performed using HPLC analysis wherein the samples were studied after different time intervals. The 0.6 mg of MJ04 was taken and dissolved in 1 mL of ethanol to achieve 0.6 mg/mL concentration. Out of this, 500 μL was further diluted to 0.3 mg/mL by adding 500 μL ethanol to it (Ethanol sample). To remaining 500 μL, we added 500 μL PEG- 300 to achieve final concentration of 0.3 mg /mL (Ethanol: PEG sample). HP LC of these samples was performed at various time points (0 h, 12 h, 24h and 48 h) upon incubating at room temperature. The HPLC of samples was performed by RP-18 end capped; 5 μM, 4.6 x 250 mm. The mobile phase containing water (30%) and acetonitrile (70%) was used at the flow rate of 1 mL/min at a column temperature of 27 °C at 210 nm wavelength. A volume of 5 μL of sample was injected and the total run time of the assay was 30 min using the isocratic technique in the HPLC system. HPLC chromatograms are depicted in Figure A and Figure B. **S3-Table 1.** Weight of C57BL/6J mice. **S3-Table 2.** Biochemical Parameters in C57/B6 mice. **S3-Table 3.** Haematological Parameter in C57/B6 mice. **Figure S4**. MJ04 induces early onset in anagen development and hair cycle progression in nude mice (NU/J Foxn1nu). (A) MJ04 induced telogen-anagen transition, as shown by the dorsal skin turning from hairless to white hair. Digital photographs were taken from the representative area using a Nikon digital camera (n = 5 mice). (i)- Control group, (ii)-Vehicle group, (iii)- Tofacitinib group (0.8 mg/Kg), (iv)- Tofacitinib group (0.08 mg/kg), (v)- MJ04 group (0.08 mg/kg), (vi) MJ04 group (0.04 mg/kg), (vii) MJ04 group (0.016 mg/kg), (viii) Baricitinib group (0.1 mg/kg), (ix) Baricitinib group (0.04 mg/kg), and (x) Baricitinib group (0.02 mg/kg). **Figure S5.** Evaluation of toxicity of MJ04 and Tofacitinib in splenocytes by MTT assay. **Figure S6. A** Histological images of organs from subacute toxicity experiment. Organs stained by H&E was obtained from the heart, liver, lung, and brain of Wistar rats after treatment with or without MJ04 (2000 mg/Kg). **B** Pharmacokinetic profile of MJ04, illustrating its dynamic behavior and disposition in C57BL/6 mice over a defined period of time.

## Data Availability

All data needed to evaluate the conclusions in the paper are present in the paper and/or the Additional files.
