# Supplementary material for: Discovery of a novel and highly selective JAK3 inhibitor as a potent hair growth promoter
Source: J Transl Med. 2024 Apr 18;22:370. doi: 10.1186/s12967-024-05144-4 (PMC11025159; doi:10.1186/s12967-024-05144-4)
Supplement: Supplementary file 1 — Additional file 1: Figure S1. A (a) IC50 of MJ04 Km (ATP concentration). (b) IC50 of MJ04 at 1mM ATP concentration and (c) IC50 of Tofacitinib at Km ATP concentration. B–F RMSDs, RMSFs, and intermolecular interactions of MJ04 with JAK1, JAK2, and JAK3: B. Root Mean Square deviations (RMSDs). C. root mean Square Fluctuations (RMSFs) illustrating dynamic fluctuations during simulation. D, E, and F represent intermolecular interactions of MJ04 with JAK1, JAK2, and JAK3, respectively. S1. Table 1. Inhibition activities (IC50, nM) of small molecules against JAK3 kinase (Cell-free assay). S1. Table 2. Binding affinity and H-bonding interactions of ligand molecule (MJ04) in the hinge region of the kinase domain of JAK1, JAK2, and JAK3 proteins. S2 Table 1. Inhibition activities (IC50, μM) of small molecules against A549, HCT-116, Mia PaCa-2, MCF-7-2and Panc-2. Figure S2A. Schematic representation flow regarding selection of compounds against the target. S2B. General scheme for the synthesis of MJ04. S2C. 1H—NMR of MJ04. S2D. 19F—NMR of MJ04.S2E. HRMS of MJ04. Figure S3. Hair regrowth induced by MJ04 in a DHT-Induced Androgenetic Alopecia (AGA) mouse model. The experiment involved the daily treatment of the shaved dorsal skin of C57BL/6J mice with 0.5% testosterone for 1 h before the topical application of different concentrations of tofacitinib, MJ04, and baricitinib for 28 days. Digital photographs were taken from the representative area using a Nikon digital camera (n = 8 mice). (i)- Control group, (ii)-Testosterone group, (iii)-Vehicle group, (iv)- Tofacitinib group (0.8mg/Kg), (v)- Tofacitinib group (0.08 mg/kg), (vi)- MJ04 group (0.08 mg/kg), (vii) MJ04 group (0.04 mg/kg), (viii) MJ04 group (0.016 mg/kg), (ix) Baricitinib group (0.1 mg/kg), (x) Baricitinib group (0.04 mg/kg), and (xi) Baricitinib group (0.02 mg/kg). Figure S3L. Stability analysis of the formulation used for in vivo studies. The stability study of the lead compound (MJ04) was performed using HPLC analy [file 12967_2024_5144_MOESM1_ESM.pdf]

Fig S1A

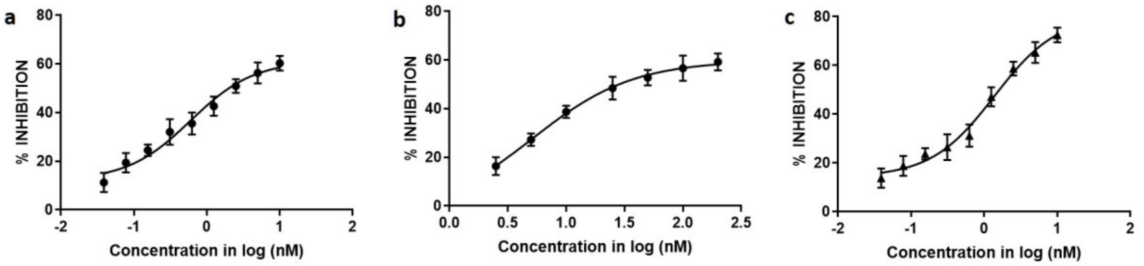

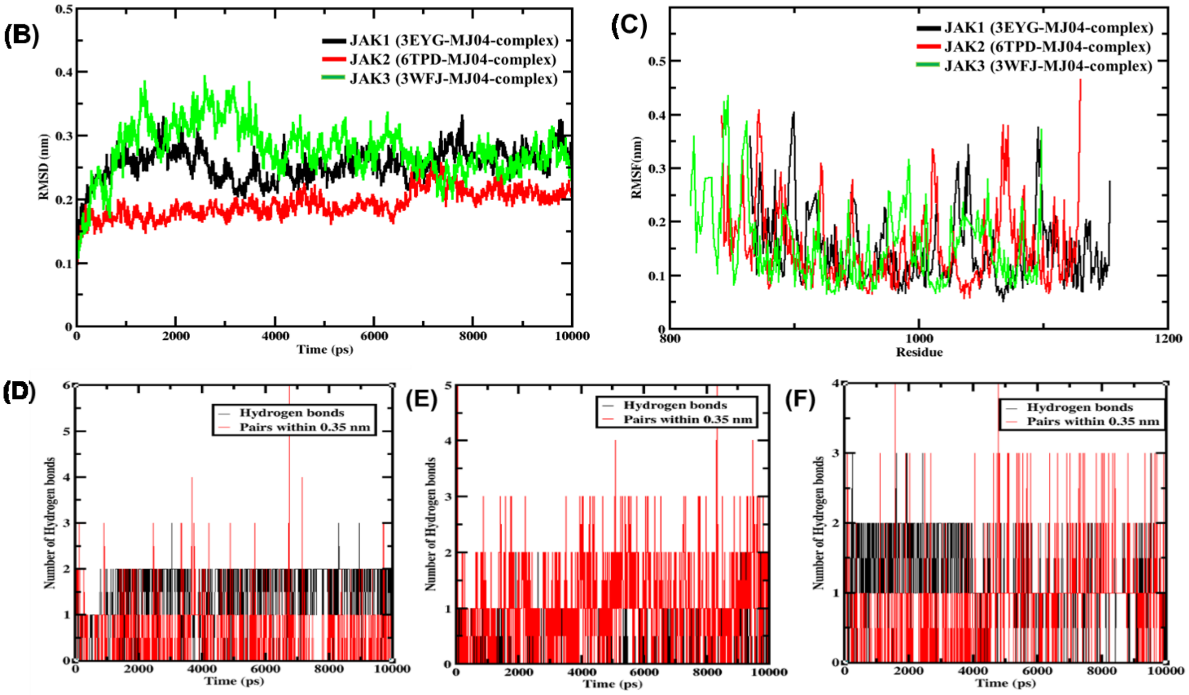

Fig S1A: (i) IC_{50} of MJ04 Km (ATP concentration). (ii) IC_{50} of MJ04 at 1mM ATP concentration and (iii) IC_{50} of Tofacitinib at Km ATP concentration.

Fig S1 (B-F): RMSDs, RMSFs, and intermolecular interactions of MJ04 with JAK1, JAK2, and JAK3: The Fig. S1 (B) Root Mean Square deviations (RMSDs) and (C) root mean Square Fluctuations (RMSFs) illustrating dynamic fluctuations during simulation. Fig. S1 D, E, and F represent intermolecular interactions of MJ-04 with JAK1, JAK2, and JAK3, respectively.

S1 Table 1: Inhibition activities (IC_{50}, nM) of small molecules against JAK3 kinase (Cell-free assay)

S.No.	Compounds	Structure	IC_{50} (nM)
1.	MJ-01	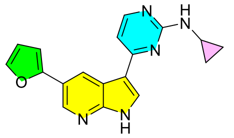	22.0
2.	MJ-02	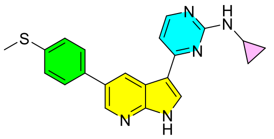	55.0
3.	MJ-03	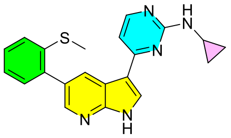	215.0
**4.**	**MJ-04**	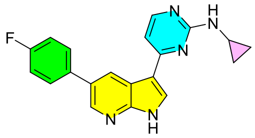	**2.03**
5.	MJ-05	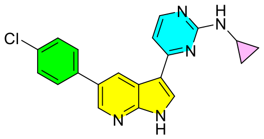	3.2
6.	MJ-06	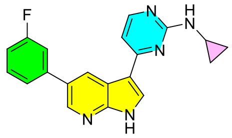	4.3
7.	MJ-07	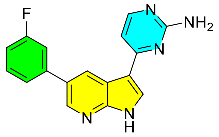	4.8
8.	MJ-08	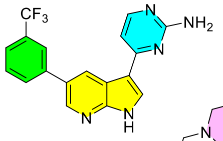	412.0
9.	MJ-09	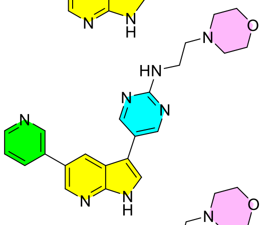	146.0
10.	MJ-10	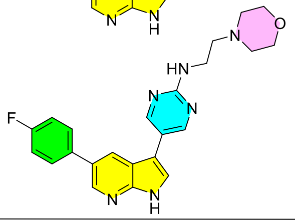	44.0

S1 –Table2

Binding affinity and H-bonding interactions of ligand molecule (**MJ-04)** in the hinge region of the kinase domain of **JAK1, JAK2,** and **JAK3** proteins.

PDB ID	Ligands/Inhibitors	Binding Affinity (kcal/mol)	H-bonding interactions (Hinge region)
**JAK1 (3EYG)**	MJ-04	-9.8	E957, L959
**JAK2 (6TPD)**	MJ-04	-9.1	L932
**JAK3 (5WFJ)**	MJ-04	-9.8	E903, L905

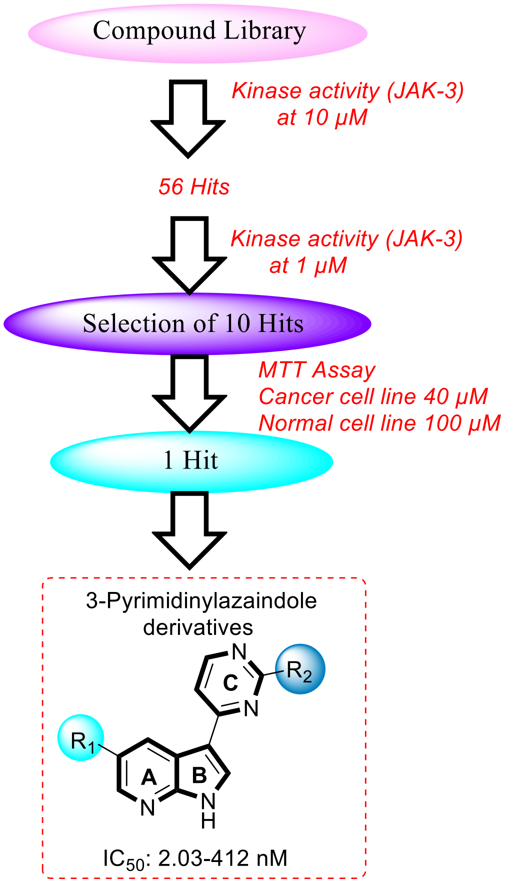

Fig S2A: Schematic representation flow regarding selection of compounds against the target

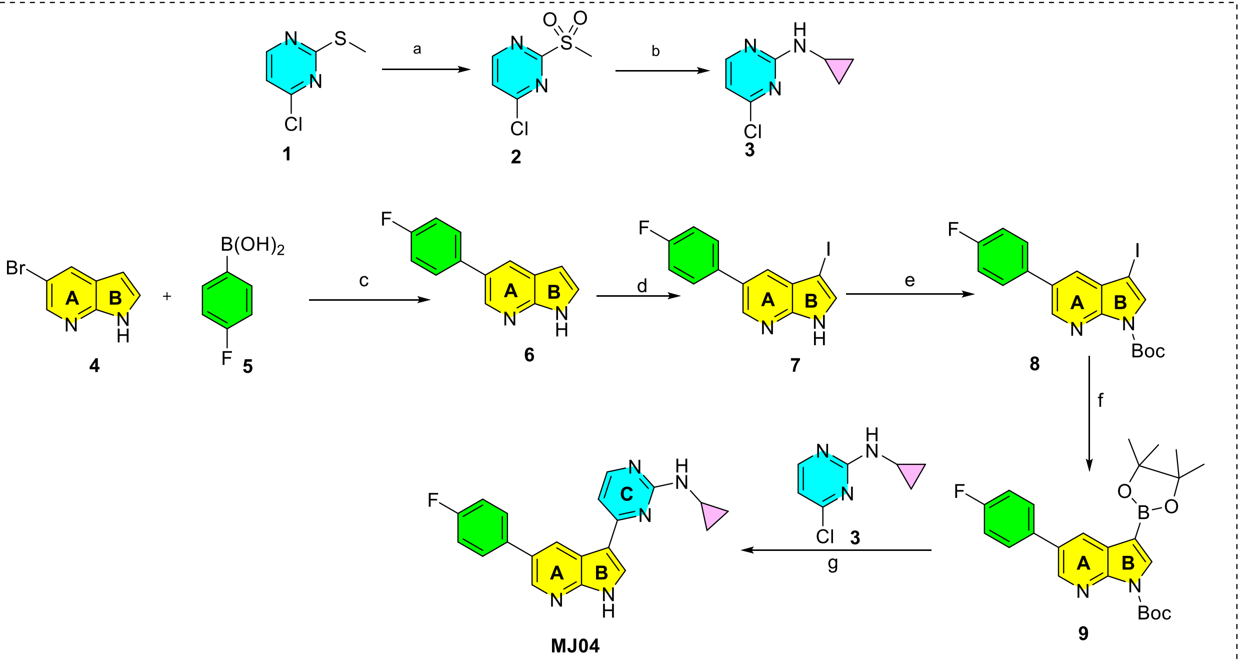

Reagents and conditions : (a) m-CPBA, CH_2Cl_2, 0 ^oC - rt, 2 h; (b) Cyclopropyl amine, THF, rt, 2 h; (c) $PdCl_2$(dppf), K_2CO_3, dioxane:H_2O, 80 ^oC, 6-12 h; (d) I_2, KOH, DMF, rt, 0.5-1 h; (e) Boc anhydride, DMAP, DCM, rt, 0.5-1 h; (f). $Pd(PPh_3)_4$, anhydrous Et_3N, anhydrous dioxane, HBPin, 80 ^oC, 3-4 h; (g) $Pd(PPh_3)_4$, **3**, Cs_2CO_3, anhydrous MeOH, 100 ^oC, 35-49 h.

Fig S2B:- General scheme for the synthesis of MJ04

Procedure for the synthesis of compound MJ04

meta-Chloroperbenzoic acid (*m*-CPBA) (1.5 mmol) was added portion wise to an ice-cooled solution of **1** (1.0 mmol) in dichloromethane, and the resulting reaction mixture was stirred at 25 ^oC for 2 h. After that completion of the reaction was monitored with the help of thin-layer chromatography, and then the reaction mixture was washed with saturated solution of sodium bicarbonate, extracted it with dichloromethane, and concentrated in-vaccuo to obtain solid compound **2**, which was used for the next step without further purification. To the solution of **2** in tetrahydrofuran, cyclopropyl amine (2.0 equiv) was added, and resulting reaction mixture was stirred at room temperature for 2 h and then solvents were removed in vaccuo after that residue was absorbed onto celite and purified chromatographically on silica gel with ethyl acetate/hexane system to obtained pure compounds **3**. Next, we started synthesis of compound 6 that began with a Suzuki cross coupling reaction between 5-bromo-7-azaindole and 4-fluoro phenylboronic acid that gave 5-aryl substituted 7-azaindole **(6)** in good yield (81 %). Iodination of 6 in presence of

iodine, potassium hydroxide gave **(7)** which on further treated with Boc anhydride gave **(8)** in good yield (84 %). Next by using tetrakis (triphenylphosphine)-palladium (0) (3 mol %) and intermediates (8) (1.00 mmol) were placed under argon atmosphere in a dry screw-cap vessel with septum. Then, 5 mL of dry dioxane was added, and the mixture was degassed with argon. Dry triethylamine (10.0 mmol, 10.0 equiv) and 4,4,5,5-tetramethyl-1,3,2- dioxaborolane (1.50 mmol, 1.50 equiv) were successively added to the mixture, which was stirred at 80 °C (preheated oil bath) for 3-4 h to obtain 9 (monitored by TLC). Then, after cooling at 25 ºC (water bath), tetrakis (triphenylphosphine)-palladium (0) (3 mol %), 5 mL of dry methanol, 1.00 mmol of compound 3, and cesium carbonate (2.50 mmol, 2.50 equiv) were successively added, and the mixture was stirred at 100 °C (preheated oil bath) for 35-49 h. Then, after cooling at 25 ºC (water bath), the solvents were removed in vaccuo, and the residue was absorbed onto Celite and purified chromatographically on silica gel 230-400 with dichloromethane-methanol aqueous ammonia (isocratic or stepwise gradient). The obtained bis(hetero)aryls can be further purified by suspending them in dichloromethane, sonication in an ultrasound bath for 0.5- 1.0 h, filtration, and drying in vaccuo overnight for 12 h to obtain the compound MJ04.

Spectral Data of Compound MJ04

TLC (MeOH: DCM (1:9)) R_f= 0.6; Yield: 54 % Yellow Solid, m.p. = 243 - 245 ºC. ^{1}H NMR (400 MHz, DMSO d^6): δ 12.32 (s, 1H), 8.55 (d, 4 Hz, 1H), 8.44 (d, J = 4 Hz, 1H), 8.20 (d, J = 4 Hz, 1H), 7.75 (m, 2H), 7.35 - 7.31 (m, 3H), 7.15 (d, J = 4 Hz, 1H), 2.82 (s, 1H), 1.92 (s, 1H), 0.71- 0.68 (m, 2H), 0.57- 0.53 (m, 2H) ppm. HRMS (ESI-TOF) calculated for $C_{20}H_{17}FN_5$ [M + H^+] 346.1465

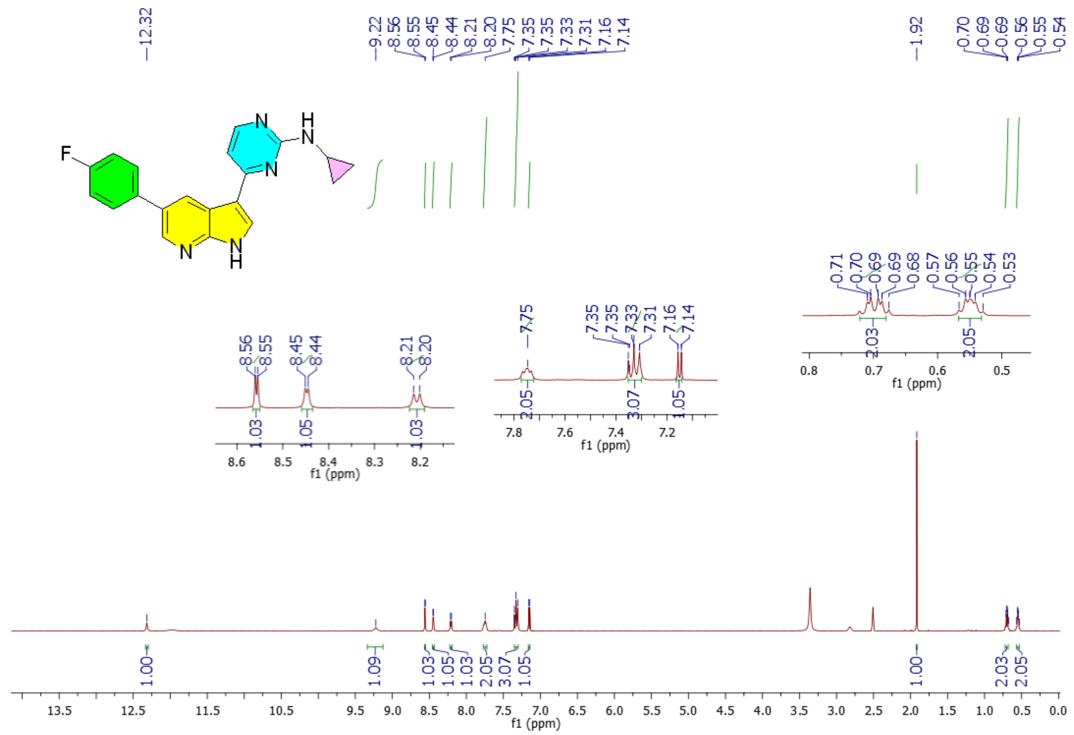

Fig 2SC:- ^{1}H - NMR of MJ04

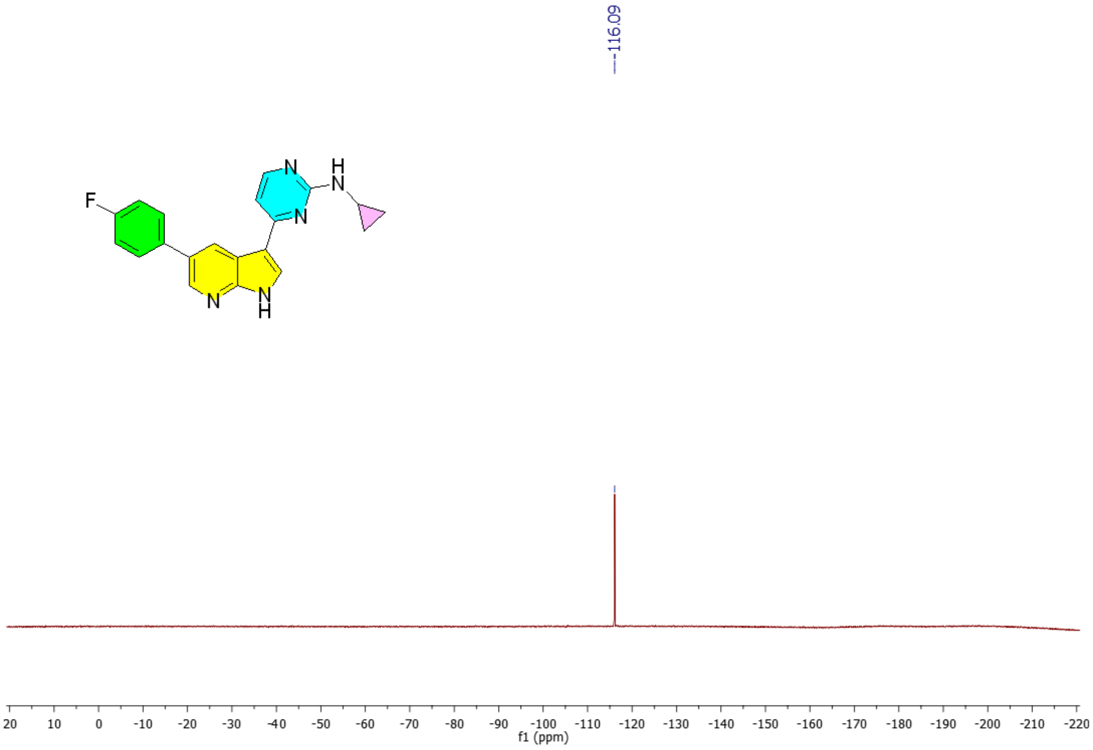

Fig 2SD:- ^{19}F - NMR of MJ04

Single Mass Analysis
Tolerance = 50.0 PPM / DBE: min = -1.5, max = 50.0
Element prediction: Off
Number of isotope peaks used for i-FIT = 3

Monoisotopic Mass, Even Electron Ions
16 formula(e) evaluated with 1 results within limits (up to 3 closest results for each mass)
Elements Used:
C: 0-20 H: 0-100 N: 0-5 F: 0-1

IIIM-4F | QMI DIVISION, CSIR-IIIM JAMMU | 25-Sep-2023
Xevo G2-XS QTOF YFC2015 | 13:33:10
250923_03 8 (0.172) | 1: TOF MS ES+ | 9.14e+006

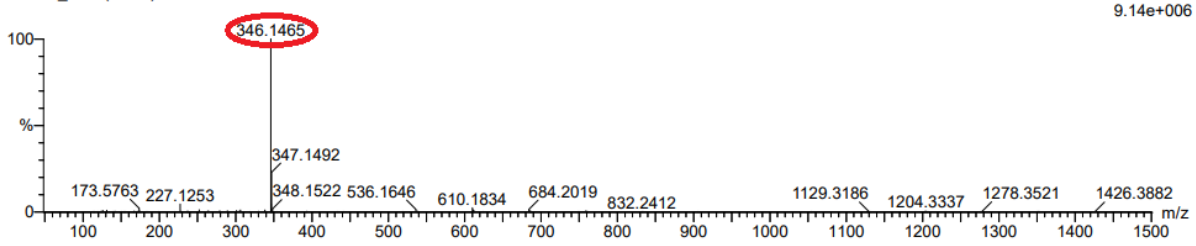

Minimum:				-1.5				
Maximum:		2.0	50.0	50.0				
Mass	Calc. Mass	mDa	PPM	DBE	i-FIT	Norm	Conf(%)	Formula
346.1465	346.1468	-0.3	-0.9	14.5	866.1	n/a	n/a	C20 H17 N5 F

Fig S2E:- HRMS of MJ04

S2 Table 1:- Inhibition activities (IC50, µM) of small molecules against A549, HCT-116, Mia PaCa-2, MCF-7-2and Panc-2

S. No:	Name	Structure	IC_{50} (µM)					
			A549	HCT-116	Mia PaCa-2	MCF-7	Panc-2	HEK-293
1	MJ01	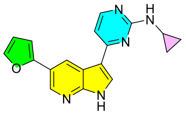	0.47	1.89	8.30	1.65	3.16	>100
2	MJ02	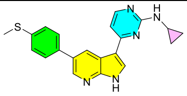	3.13	3.12	7.10	1.83	4.67	>100
3	MJ03	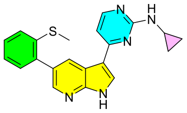	2.88	8.20	18.12	10.22	11.01	>100
4	MJ04	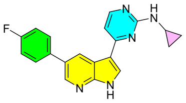	3.79	2.60	2.27	4.47	2.77	>100
5	MJ05	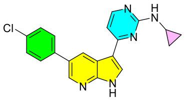	3.71	8.72	6.59	4.47	7.76	>100
6	MJ06	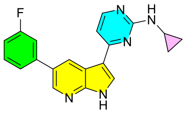	4.31	1.52	3.66	3.31	7.33	>100
7	MJ07	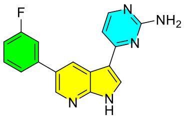	5.55	6.34	3.22	1.38	3.12	>100
8	MJ08	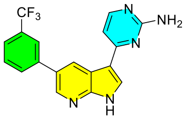	5.10	1.18	5.38	6.88	19.28	>100
9	MJ09	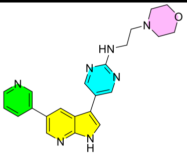	5.74	3.22	2.16	11.48	26.29	>100
10	MJ10	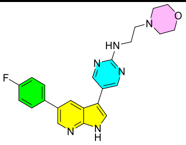	6.99	5.36	6.71	8.61	33.63	>100

Fig-S3A

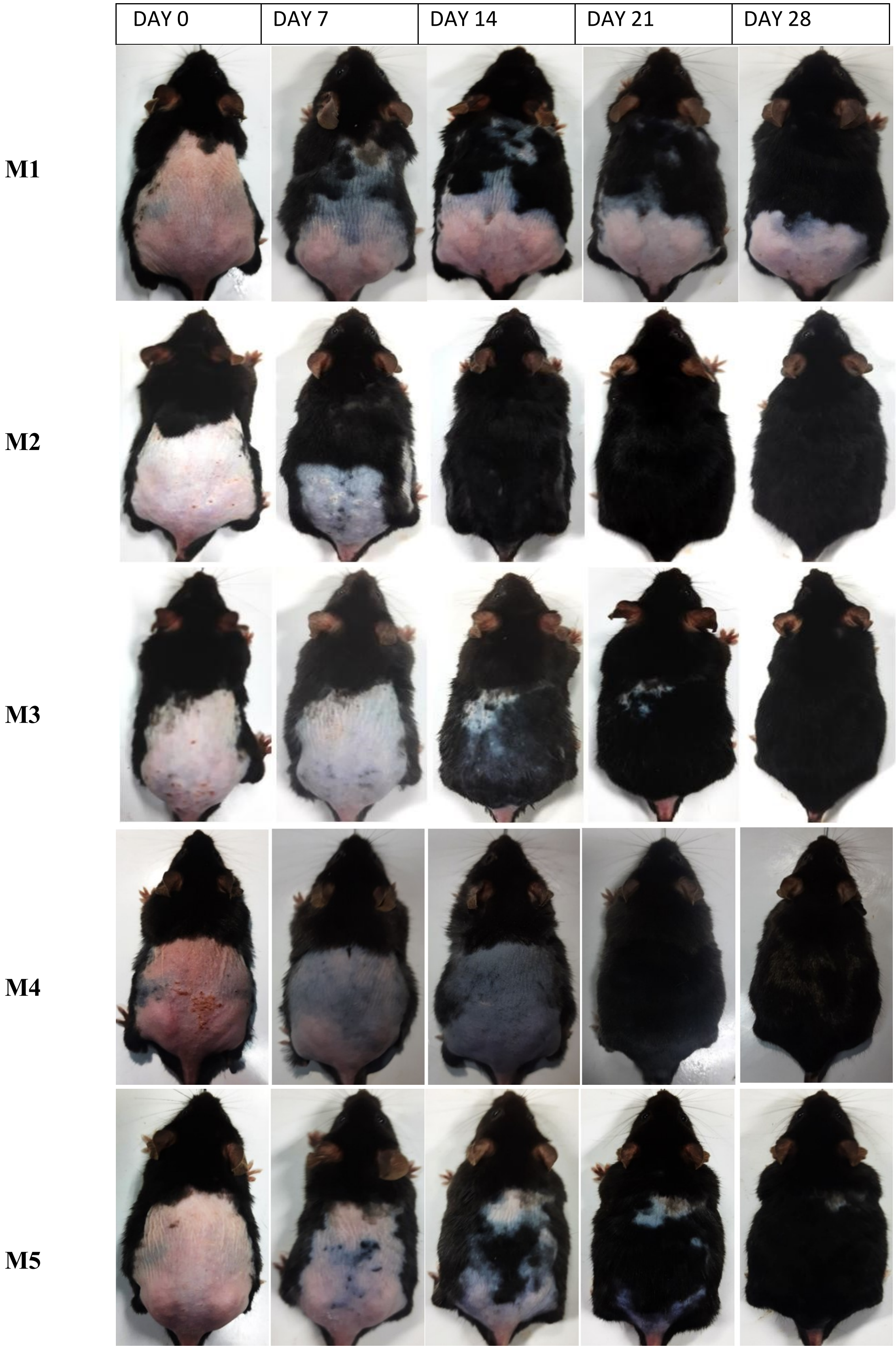

Fig-S3B

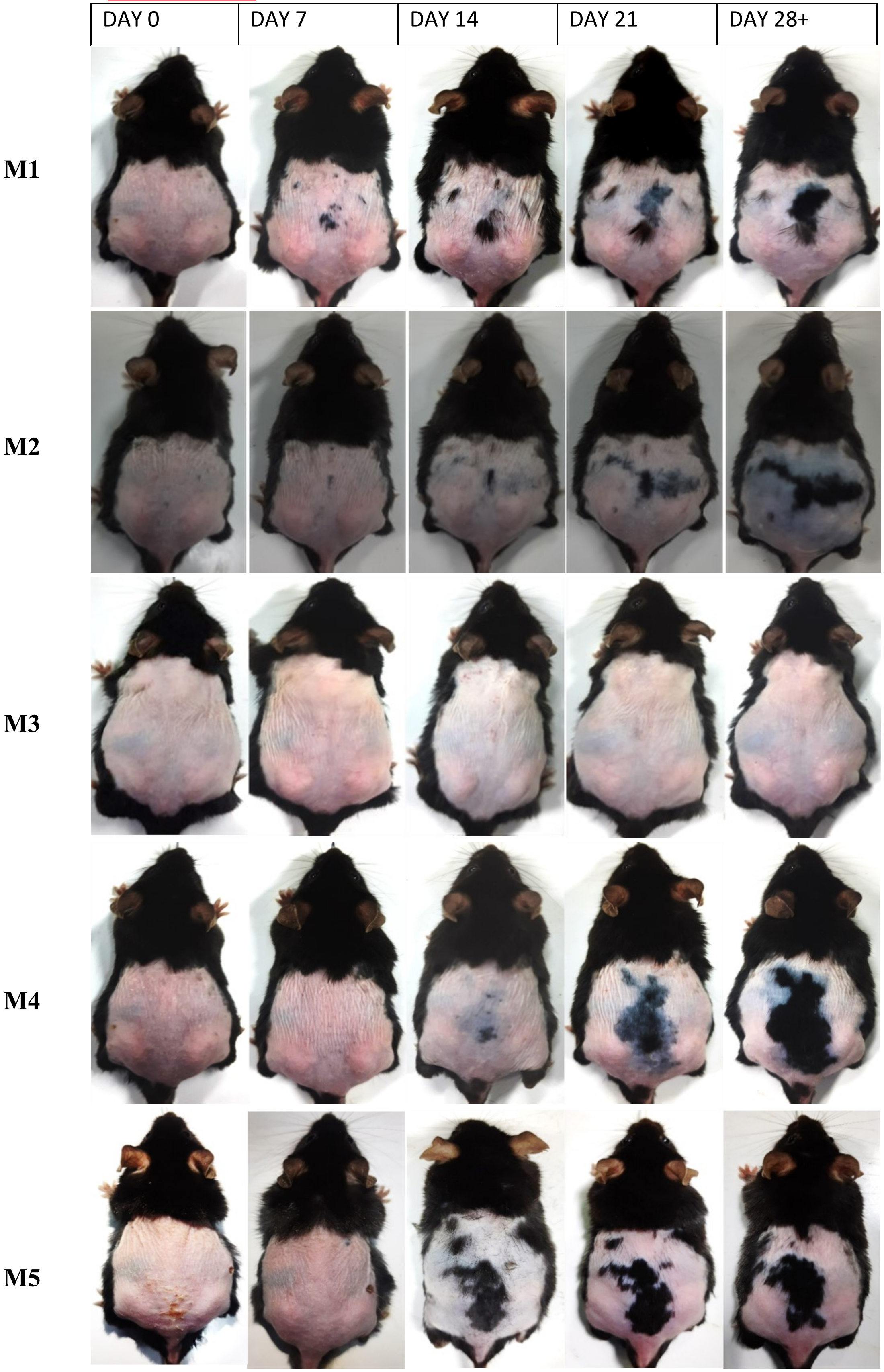

Fig-S3C

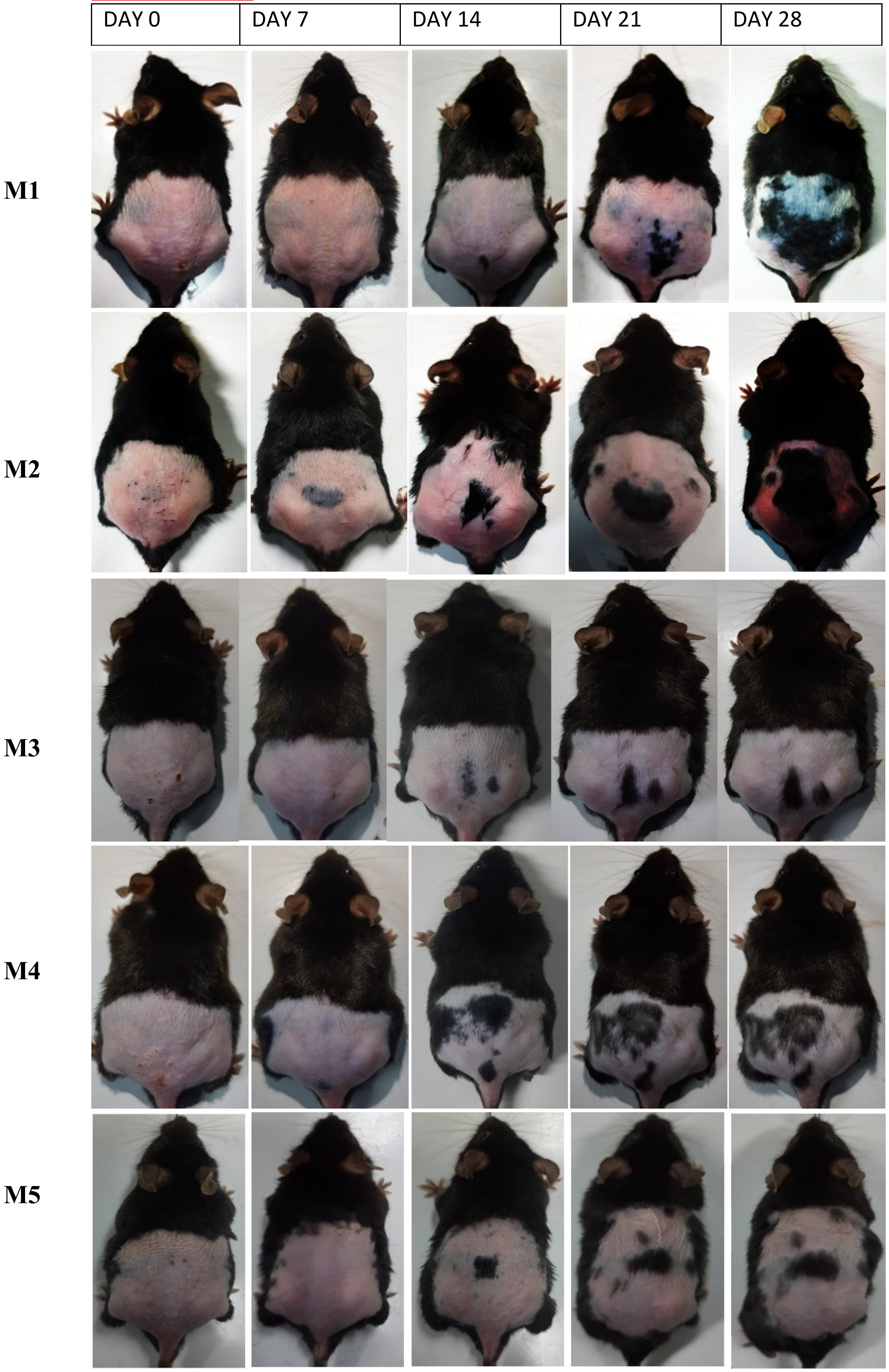

Fig-S3D

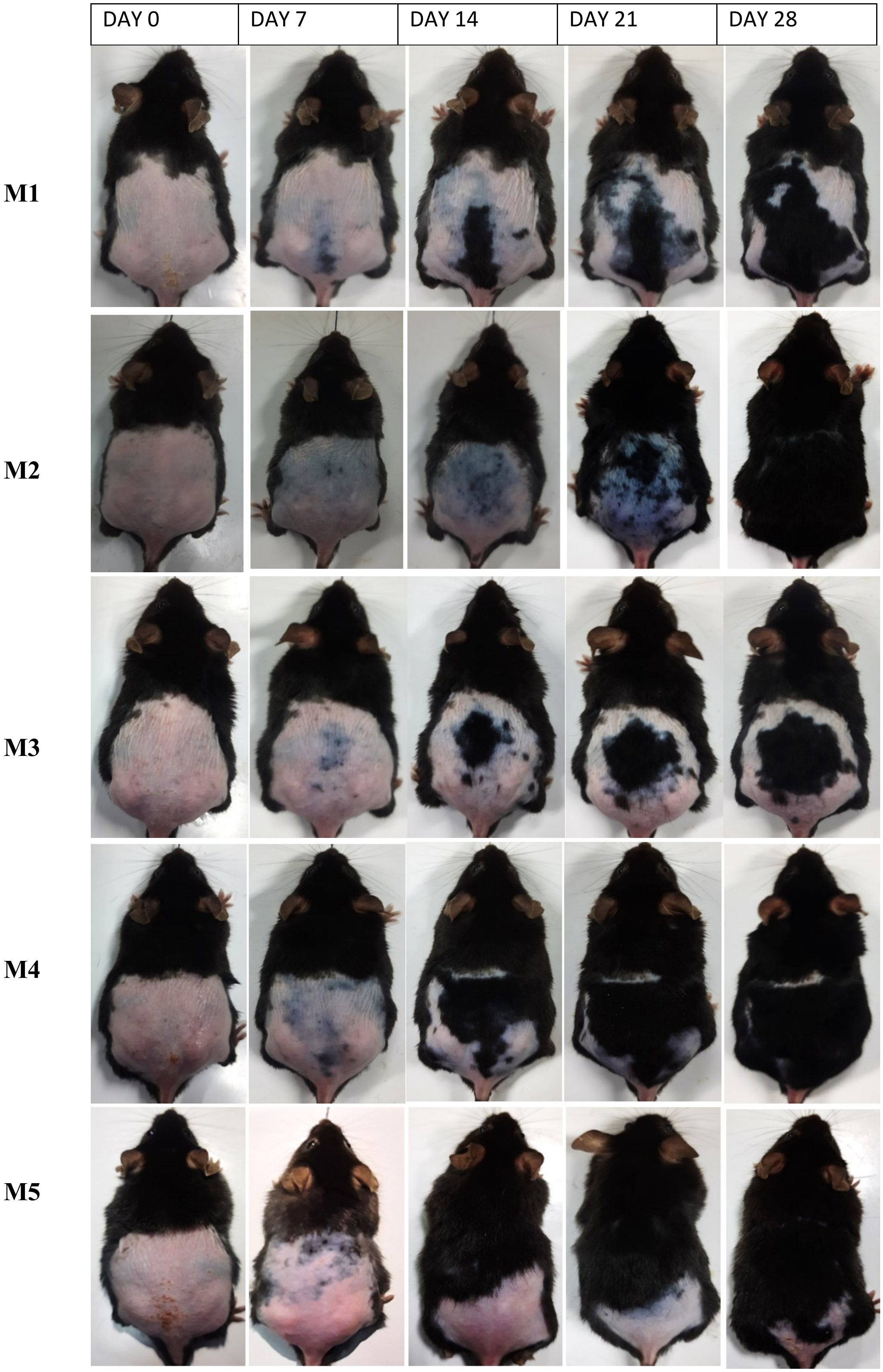

Fig-S3E

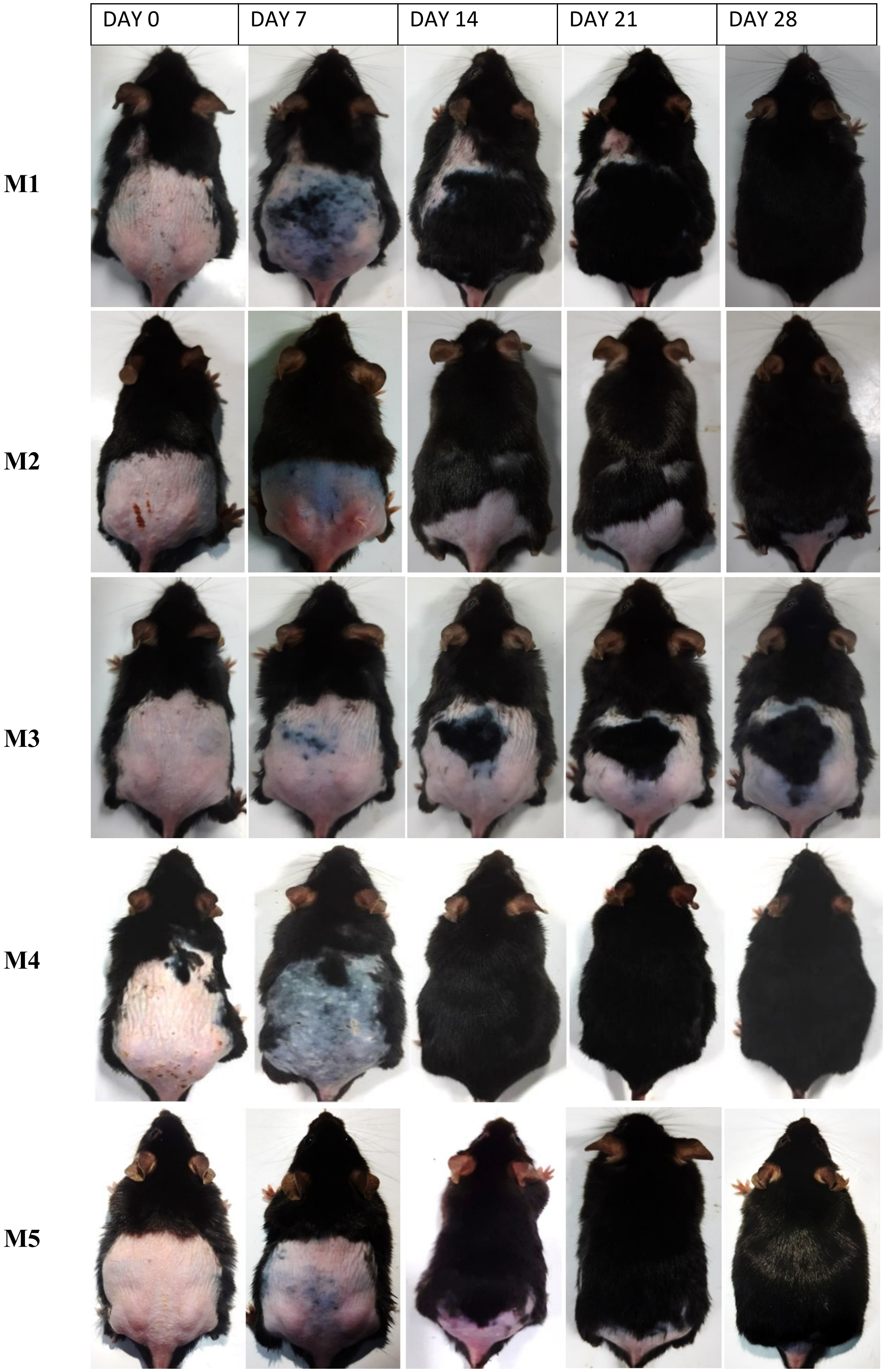

Fig-S3F

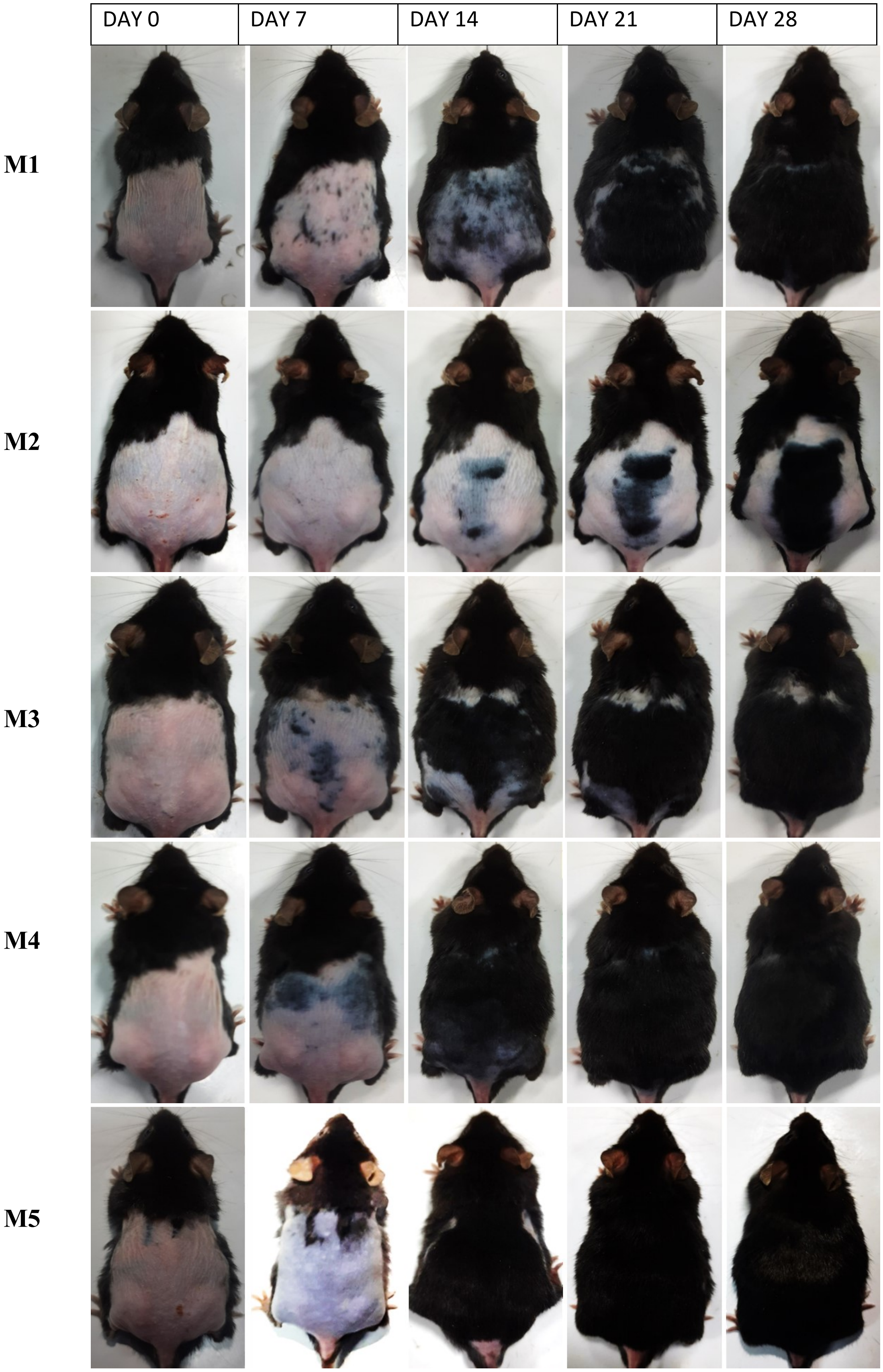

Fig-S3G

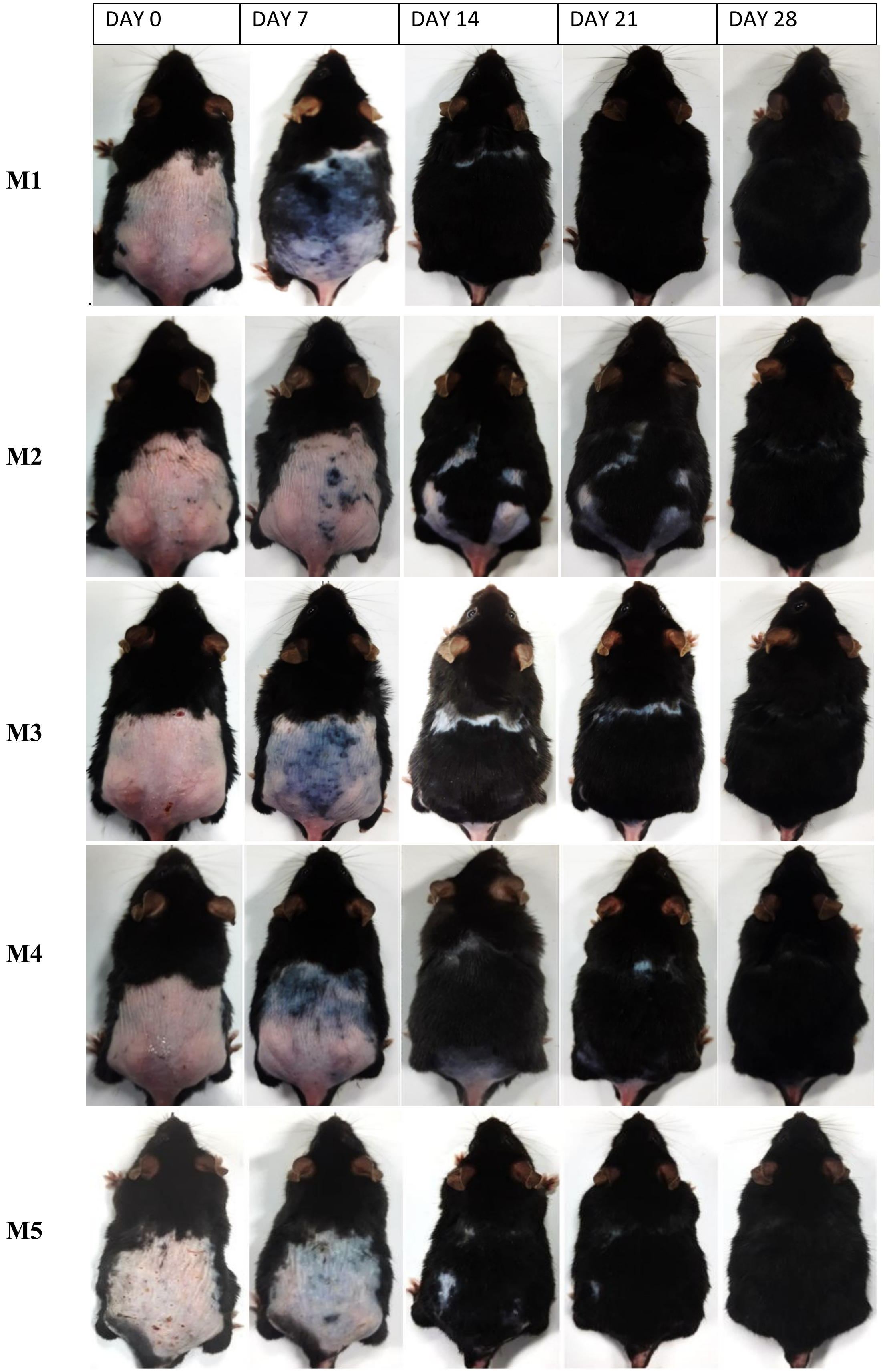

Fig-S3H

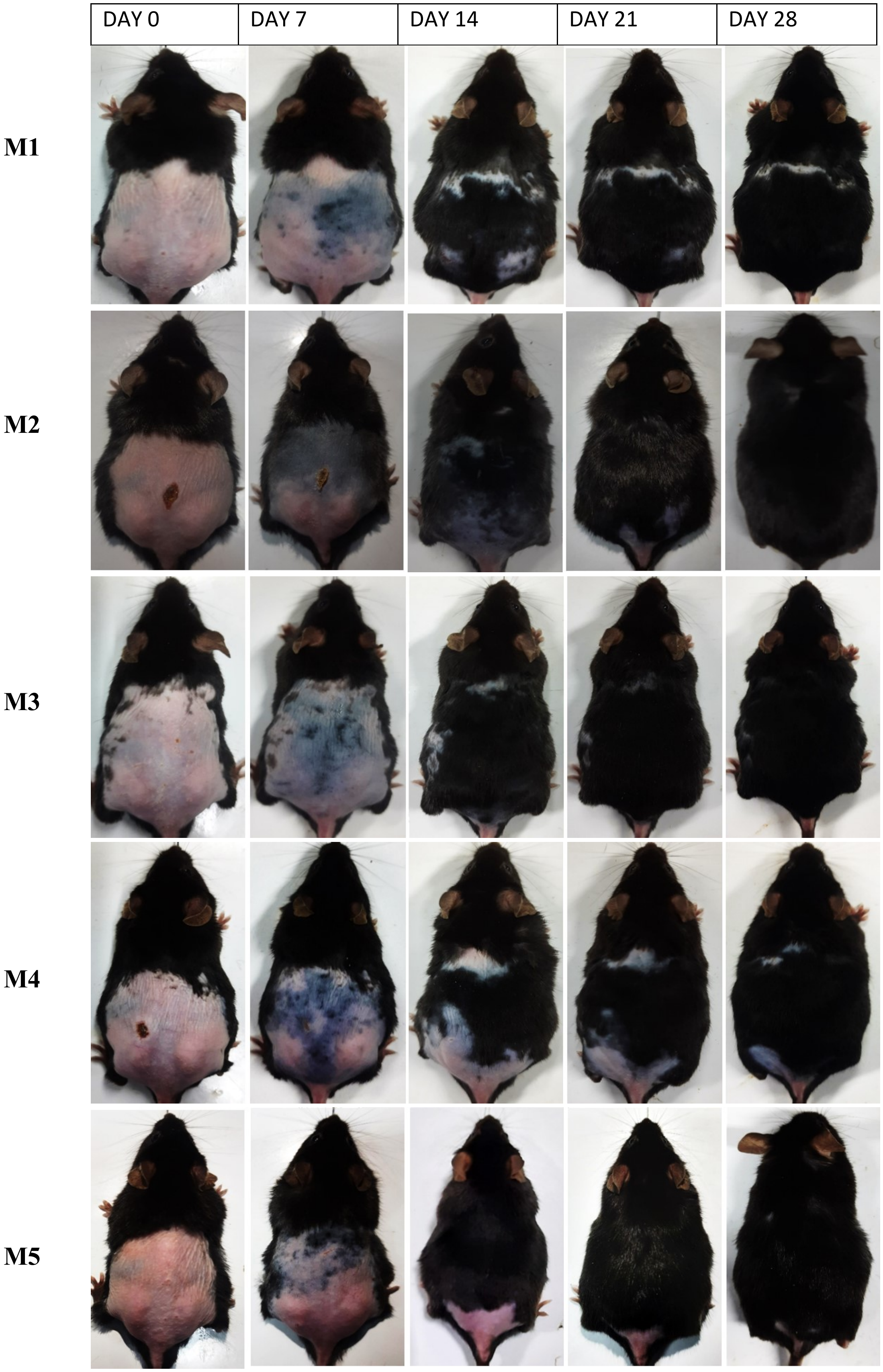

Fig-S3I

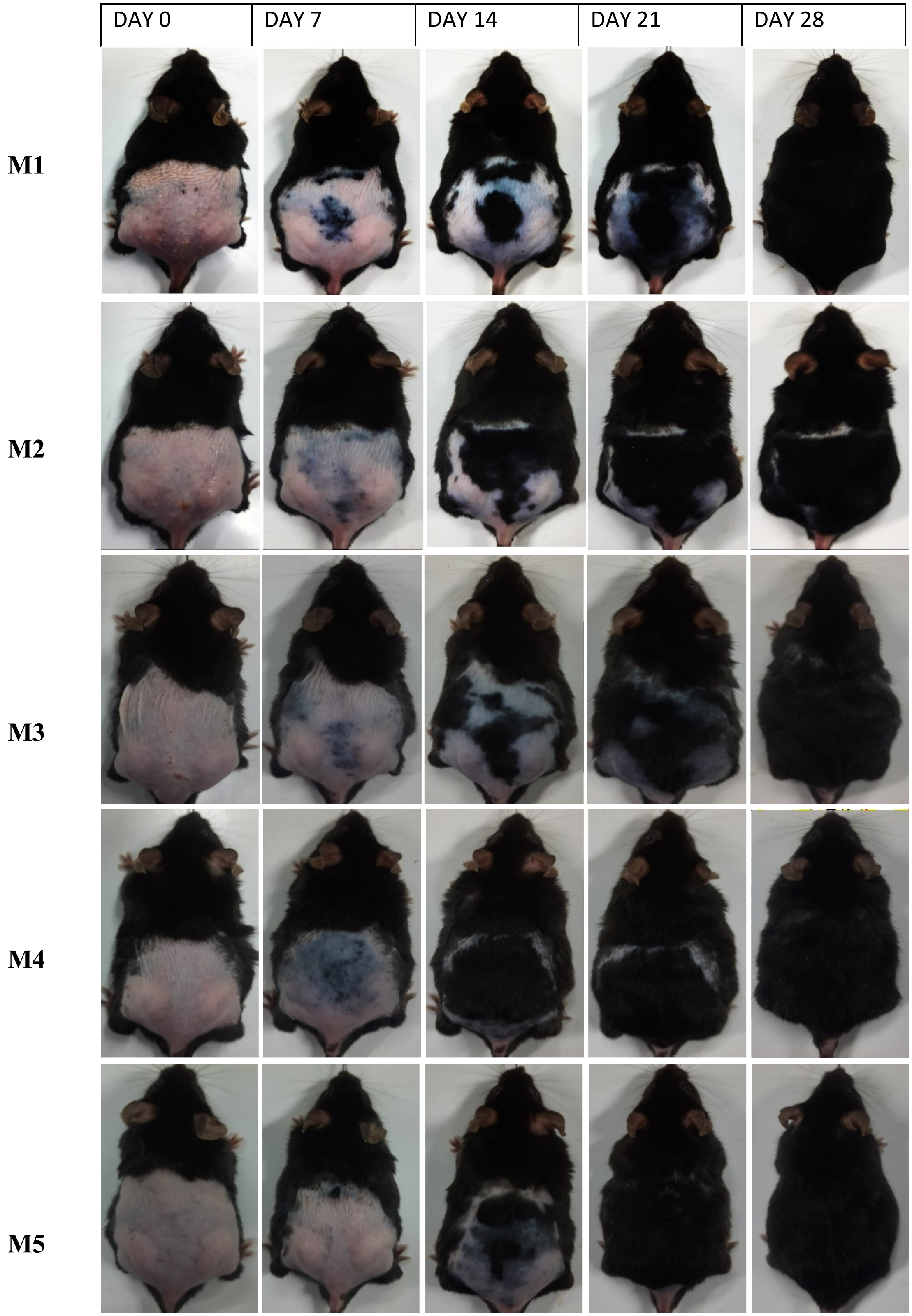

Fig-S3J

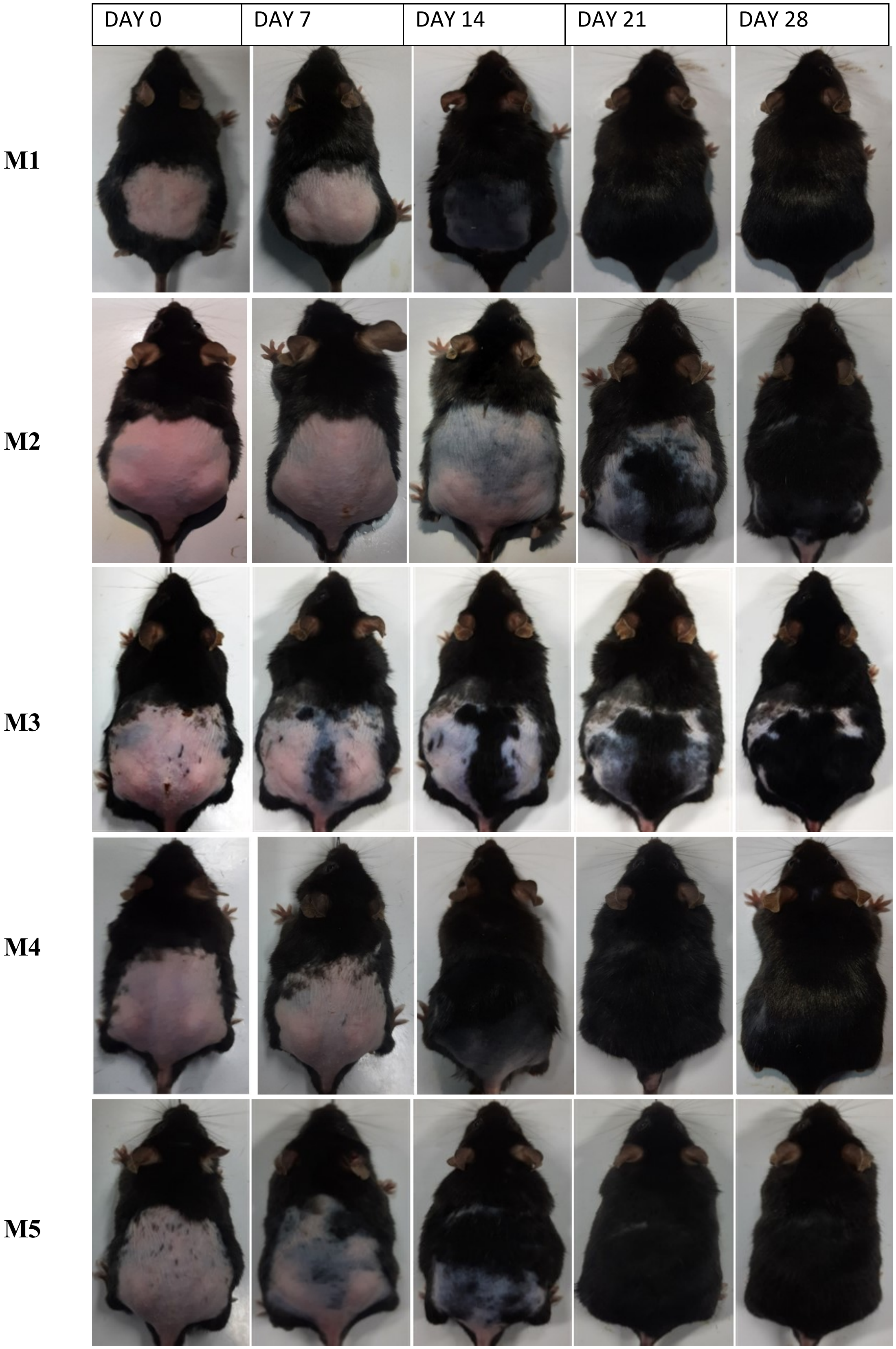

Fig-S3K

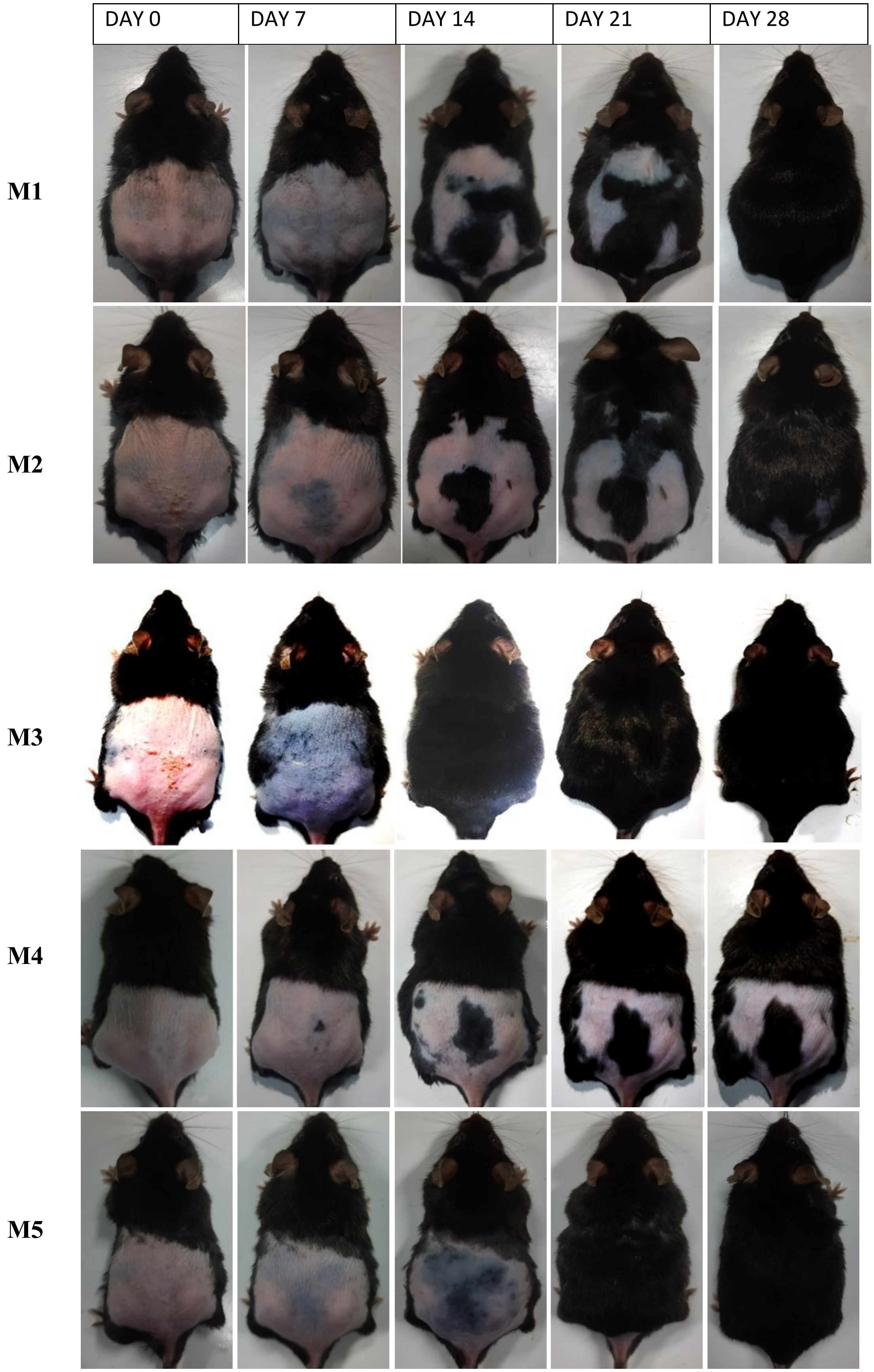

Figure S3: Hair regrowth induced by MJ04 in a DHT-Induced Androgenetic Alopecia (AGA) mouse model. The experiment involved the daily treatment of the shaved dorsal skin of C57BL/6J mice with 0.5% testosterone for 1 hour before the topical application of different concentrations of tofacitinib, MJ04, and baricitinib for 28 days. Digital photographs were taken from the representative area using a Nikon digital camera (n = 8 mice). Fig S3(i)- Control group, Fig S3(ii)-Testosterone group, Fig S3(iii)-Vehicle group, Fig S3(iv)- Tofacitinib group (0.8mg/Kg), Fig S3(v)- Tofacitinib group (0.08 mg/kg), Fig S3(vi)- MJ04 group (0.08 mg/kg), Fig S3(vii) MJ04 group (0.04 mg/kg), Fig S3(viii) MJ04 group (0.016 mg/kg), Fig S3(ix) Baricitinib group (0.1 mg/kg), Fig S3(x) Baricitinib group (0.04 mg/kg), and Fig S3(xi) Baricitinib group (0.02 mg/kg).

S3 Table 1: - Weight of C57BL/6J mice

		DAY 1	DAY 7	DAY 14	DAY 21	DAY 28
Control	M1	24.7	26.4	28.4	32.4	34.8
	M2	24.9	26.7	29.1	32.7	35.6
	M3	25.3	27.3	29.7	33.5	35.8
	M4	25.6	27.8	30.4	33.9	36.4
	M5	25.7	28.1	30.8	32.8	37.2
	M6	25.9	28.4	31.6	33.7	
	M7	26.6	29.3	32.4		
	M8	26.8	29.8			
Tesostrone	M1	24.9	26.4	28.9	31.2	33.7
	M2	25.4	27.2	29.7	31.5	33.8
	M3	25.6	27.8	30.2	32.8	34.2
	M4	25.6	28.3	31.5	33.2	35.1
	M5	25.8	28.9	32.1	34.5	36.4
	M6	26.2	29.6	32.5	34.9	
	M7	26.7	29.7	33.4		
	M8	26.9	30.1			
Vehicle	M1	24.8	26.3	28.1	30.3	33.4
	M2	24.9	26.7	28.4	30.8	34.2
	M3	25.4	27.6	28.9	31.8	35.2
	M4	25.7	28.3	29.4	32.9	36.2
	M5	26.2	28.8	30.1	33.3	36.8
	M6	26.5	29.7	31.8	34.6	
	M7	26.8	29.7	32.1		
	M8	26.3	28.9			
Tofa (0.8 mg/Kg)	M1	24.8	26.1	27.4	30.2	31.2
	M2	25.2	26.4	28.3	30.2	31.2
	M3	25.3	26.5	28.3	30.4	31.5
	M4	25.4	26.7	28.7	30.4	31.7
	M5	25.6	26.8	28.7	31.2	32.5
	M6	25.7	27.4	29.4	31.6	
	M7	26.3	27.8	29.7		
	M8	27.3	28.4			
Tofa (0.08 mg/Kg)	M1	24.3	25.7	27.6	28.9	30.3
	M2	24.3	25.8	27.9	29.4	30.4
	M3	24.5	25.8	28.4	29.8	31.2
	M4	24.9	26.3	29.1	30.4	32.1
	M5	25.7	26.9	29.3	30.7	32.3
	M6	25.9	27.8	29.3	30.8	
	M7	26.7	27.9	29.4		

	M8	27.2	28.4			
**MJ 04 (0.08 mg/Kg)**	M1	24.8	25.5	27.1	28.6	30.2
	M2	25.9	25.7	27.4	29.4	30.3
	M3	25.4	26.7	27.6	29.4	31.2
	M4	26.4	26.7	28.1	29.7	31.3
	M5	25.4	26.7	28.1	30.1	31.3
	M6	24.2	27.3	28.4	30.7	
	M7	26.4	27.5	29.3		
	M8	25.6	28.37			
**MJ 04 (0.04 mg/Kg)**	M1	24.2	26.1	28.1	29.4	30.3
	M2	24.6	26.3	28.3	29.7	31.2
	M3	25.6	26.4	28.3	29.8	31.2
	M4	25.6	26.7	28.5	30.1	31.3
	M5	25.7	26.9	28.6	30.4	31.5
	M6	25.8	27.1	28.7	30.4	
	M7	26.3	27.2	29.6		
	M8	27.4	28.7			
**MJ 04 (0.016 mg/Kg)**	M1	23.8	25.1	26.4	27.6	30.3
	M2	24.6	26.1	27.3	28.7	30.4
	M3	24.7	26.1	27.4	28.9	30.4
	M4	25.6	26.9	28.1	29.7	31.6
	M5	25.7	27.1	28.4	30.1	31.7
	M6	25.8	27.3	28.7	30.4	
	M7	26.4	27.4	29.3		
	M8	27.2	28.1			
**Bari (0.1 mg/ Kg)**	M1	24.4	26.8	29.4	30.2	33.1
	M2	24.5	26.8	29.3	31.4	34.2
	M3	24.8	27.4	30.1	31.6	34.3
	M4	24.9	28.3	29.8	32.4	34.9
	M5	25.2	28.7	30.2	33.1	35.6
	M6	25.6	29.5	31.2	33.9	
	M7	25.8	30.1	31.8		
	M8	27.13	31.2			
**Bari (0.04 mg/Kg)**	M1	23.9	25.4	26.7	29.6	31.2
	M2	24.6	26.7	28.1	30.2	31.5
	M3	25.4	26.7	28.6	30.4	31.8
	M4	25.5	27.4	28.9	30.4	32.1
	M5	25.6	27.5	29.1	31.2	33.5
	M6	25.9	27.8	29.8	31.3	
	M7	26.3	28.3	30.1		
	M8	26.7	28.4			
	M1	23.6	25.2	26.4	28.9	30.7

**Bari (0.02 mg/Kg)**	M2	24.8	26.1	27.6	29.3	30.9
	M3	24.8	26.5	27.6	29.6	31.2
	M4	25.4	26.5	27.9	30.1	31.5
	M5	25.3	26.8	29.4	30.7	32.3
	M6	25.7	27.3	29.6	31.2	
	M7	26.3	28.2	30.1		
	M8	26.9	28.7			

S3-Table 2 Biochemical Parameters in C57/B6 mice

Day 21	Group 1	Group 2	Group 3	Group 4	Group 5	Group 6	Group 7	Group 8	Group 9	Group 10	Group 11
ALP (U/L)	138	107	207	132	198	111	121	99	144	99	108
Creatine (mg/dL)	0.61	0.57	0.60	0.63	0.49	0.49	0.69	0.72	0.48	0.69	0.72
Triglyceride (mg/dL)	94	72	95	118	141	108	127	114	119	139	98
Cholesterol (mg/dL)	194	137	212	211	216	184	138	154	173	171	171
HDL(mg/dL)	114	91	126	116	110	105	111	112	108	122	105
LDL (mg/dL)	113	79	125	126	134	102	101	103	100	106	101
Total protein (g/dL)	5.5	5.2	5.2	5.2	5.0	5.2	4.9	5.2	5.0	4.9	5.0
AST/SGOT (U/L)	190	115	96	111	80	114	148	143	104	98	143
ALT/SGPT (U/L)	86	63	94	56	82	58	74	86	92	64	74
Urea (mg/dL)	55	60	43	71	49	71	49	55	51	54	69
**Day 28**	**Group 1**	**Group 2**	**Group 3**	**Group 4**	**Group 5**	**Group 6**	**Group 7**	**Group 8**	**Group 9**	**Group 10**	**Group 11**
ALP (U/L)	159	196	235	252	199	189	243	193	184	174	159

**Creatine (mg/dL)**	0.17	0.13	0.14	0.12	0.13	0.13	0.14	0.20	0.13	0.11	0.16
**Triglyceride (mg/dL)**	113	62	57	132	135	174	122	132	72	123	90
**Cholesterol (mg/dL)**	161	210	184	240	238	194	165	194	164	182	213
**HDL(mg/dL)**	98	122	115	128	133	106	108	94	134	143	173
**LDL (mg/dL)**	70	99	85	113	111	78	91	109	78	85	98
**Total protein (g/dL)**	6.2	5.5	5.5	6.1	6.5	6.2	5.8	6.3	6.2	6.3	5.8
**AST/SGOT (U/L)**	89	79	89	125	136	113	107	75	86	123	129
**ALT/SGPT (U/L)**	76	74	63	134	177	63	69	85	74	88	71
**Urea (mg/dL)**	63	52	56	50	62	79	52	54	53	66	60

Group 1- No treatment, Group 2- treated with 0.5% testosterone, Group 3 - treated with 0.5% testosterone followed by vehicle treatment after 1 h, Group 4 - treated with 0.5% testosterone followed by Tofacitinib citrate (0.8 mg/Kg) treatment after 1 h, Group 5 - treated with 0.5% testosterone followed by Tofacitinib citrate (0.08 mg/Kg) treatment after 1 h, Group 6- treated with 0.5% testosterone followed by MJ04 (0.08 mg/Kg) treatment after 1 h, Group 7- treated with 0.5% testosterone followed by MJ04 (0.04 mg/Kg) treatment after 1 h, Group 8- treated with 0.5% testosterone followed by MJ04 (0.016 mg/Kg) treatment after 1 h, Group 9 - treated with 0.5% testosterone followed by Baricitinib (0.1mg/Kg) after 1 h, Group 10 - treated with 0.5% testosterone followed by Baricitinib (0.04 mg/Kg) after 1 h, and Group 11 - treated with 0.5% testosterone followed by Baricitinib (0.02 mg/Kg) after 1 h.

S3-Table 3 Haematological Parameter in C57/B6 mice

Day 21	Group 1	Group 2	Group 3	Group 4	Group 5	Group 6	Group 7	Group 8	Group 9	Group 10	Group 11
WBC (10^3/µl)	1.3	2.4	3.1	1.7	3.0	3.3	3.0	6.5	6.4	3.6	4.3
RBC (10^{12}/l)	9.7	8.5	10.0	8.1	9.2	6.9	9.0	9.7	9.6	8.6	8.2
HGB (g/l)	14	13	15	12	13	10	13	14	14	12	12
HCT (%)	50	46	49	42	45	36	46	48	48	43	45
MCV (fL)	50	54	49	52	51	50	50	50	50	52	52
MCHC (g/dL)	29	29	30	29	28	28	29	29	29	29	28
PLT (10^3/µl)	1117	1306	777	917	587	424	899	1105	1149	1082	946
NEUT (%)	15.90	22.00	17.0	21.5	20.2	32.4	39.4	25.7	26.8	30.5	30.5
LYMPH	84.1	76.7	80.1	77.9	78.8	63.0	60.3	73.3	71.3	67.4	67.4
**Day 28**	**Group 1**	**Group 2**	**Group 3**	**Group 4**	**Group 5**	**Group 6**	**Group 7**	**Group 8**	**Group 9**	**Group 10**	**Group 11**
WBC (10^3/µl)	4.7	4.6	5.2	5.6	6.7	7.1	7.5	7.8	3.9	3.1	6.3
RBC (10^6/µl)	9.0	8.3	7.2	5.1	8.1	8.6	7.5	7.4	6.4	8.0	7.8
HGB (g/dl)	13	14	13	11	14	15	14	14	14	14	15

**HCT (%)**	53.0	53.0	41.0	31.0	47.0	52.0	46.0	46.0	43.0	48.0	48.0
**MCV (fL)**	59.0	63.0	56.0	60.0	58.0	61.0	62.0	63	67	60	62
**MCHC (g/dL)**	25.0	27.0	31.0	34.0	29.0	29.0	30.0	30.0	33.0	29.0	30.0
**PLT (10^3/µl)**	2233	1766	1767	1053	1475	1362	1687	1727	1394	1427	1724

Group 1- No treatment, Group 2- treated with 0.5% testosterone, Group 3 - treated with 0.5% testosterone followed by vehicle treatment after 1 h, Group 4 - treated with 0.5% testosterone followed by Tofacitinib citrate (0.8 mg/Kg) treatment after 1 h, Group 5 - treated with 0.5% testosterone followed by Tofacitinib citrate (0.08 mg/Kg) treatment after 1 h, Group 6- treated with 0.5% testosterone followed by MJ04 (0.08 mg/Kg) treatment after 1 h, Group 7- treated with 0.5% testosterone followed by MJ04 (0.04 mg/Kg) treatment after 1 h, Group 8- treated with 0.5% testosterone followed by MJ04 (0.016 mg/Kg) treatment after 1 h, Group 9 - treated with 0.5% testosterone followed by Baricitinib (0.1mg/Kg) after 1 h, Group 10 - treated with 0.5% testosterone followed by Baricitinib (0.04 mg/Kg) after 1 h, and Group 11 - treated with 0.5% testosterone followed by Baricitinib (0.02 mg/Kg) after 1 h.

Fig S4-i

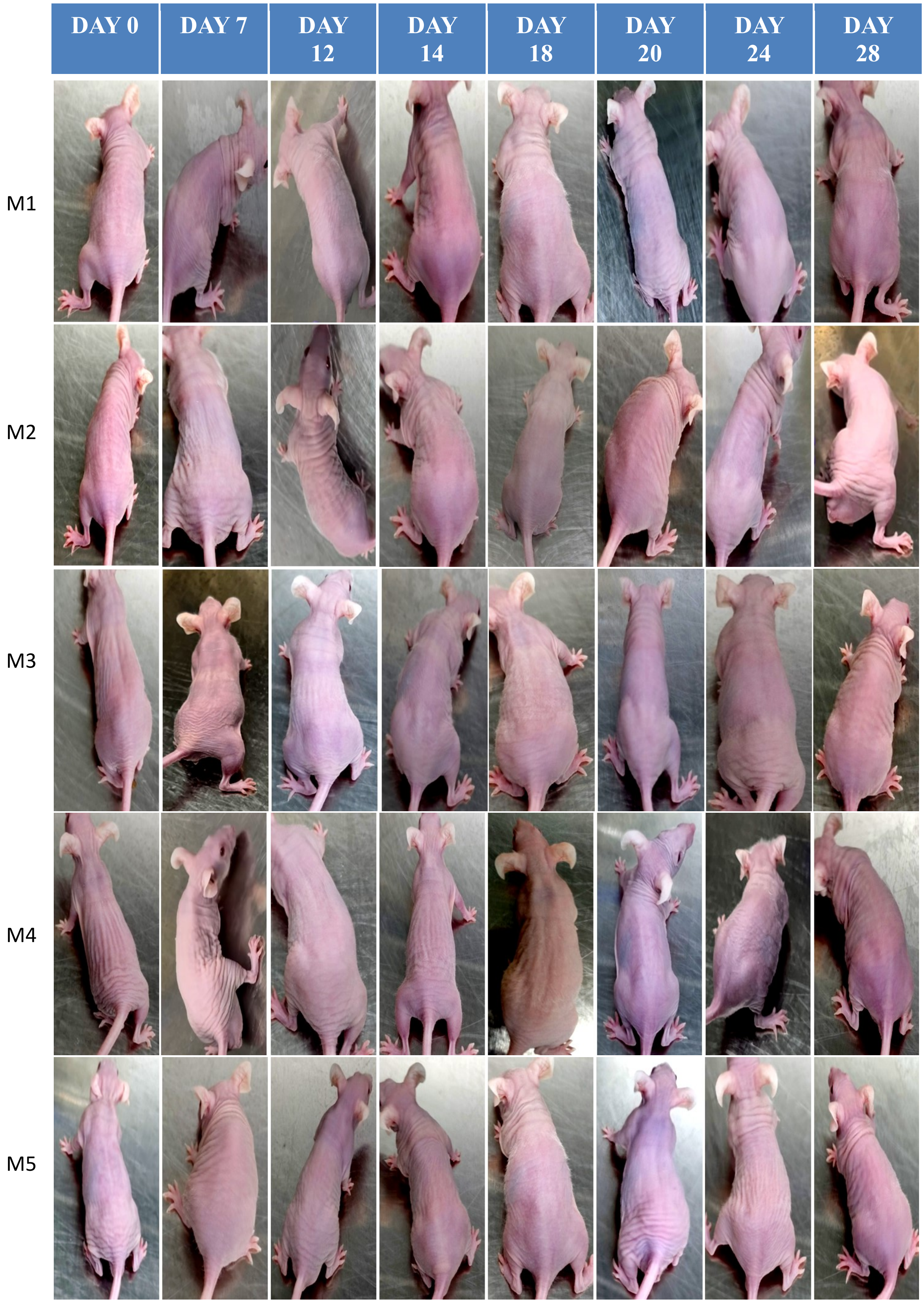

Fig S4-ii

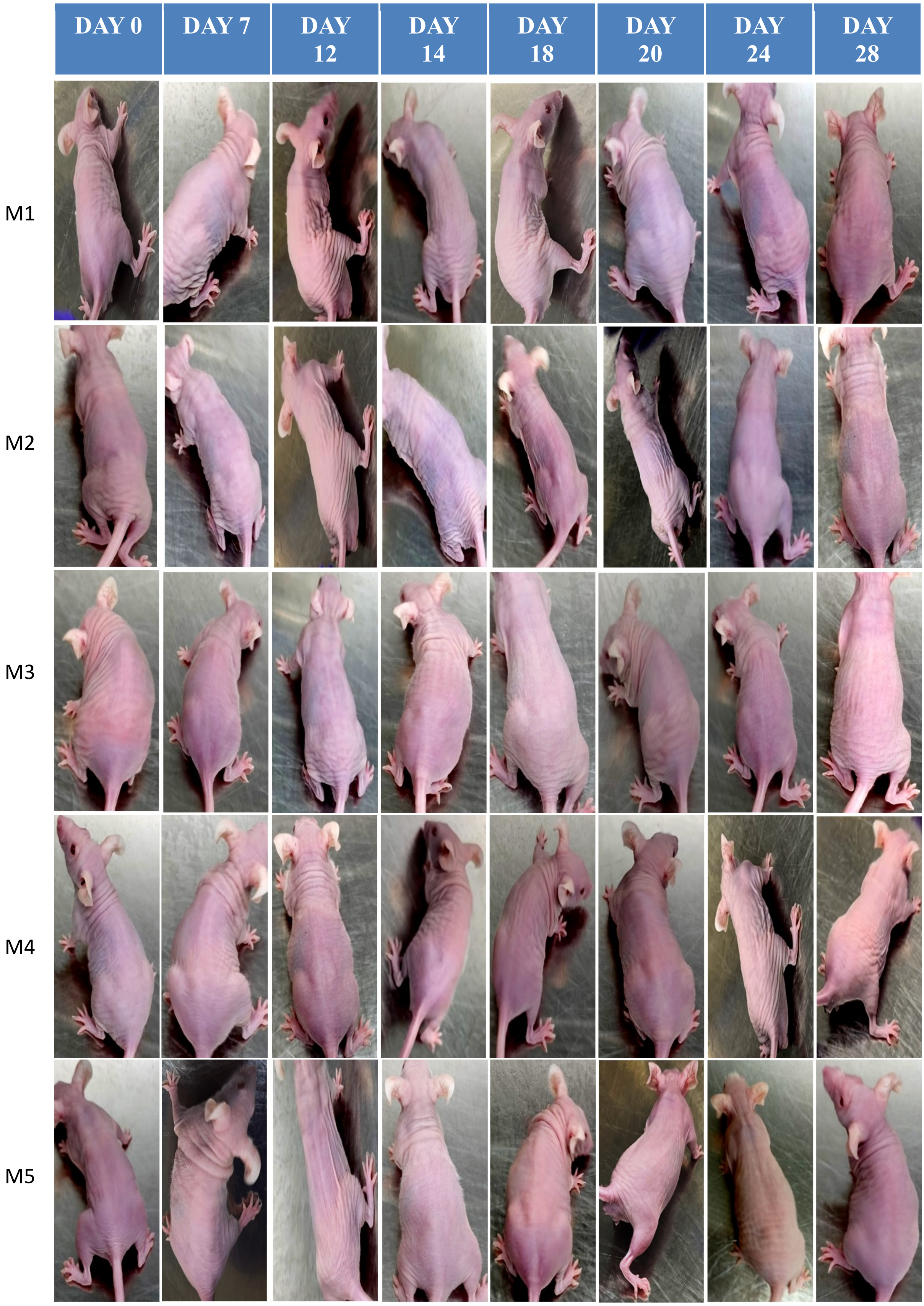

Fig S4-iii

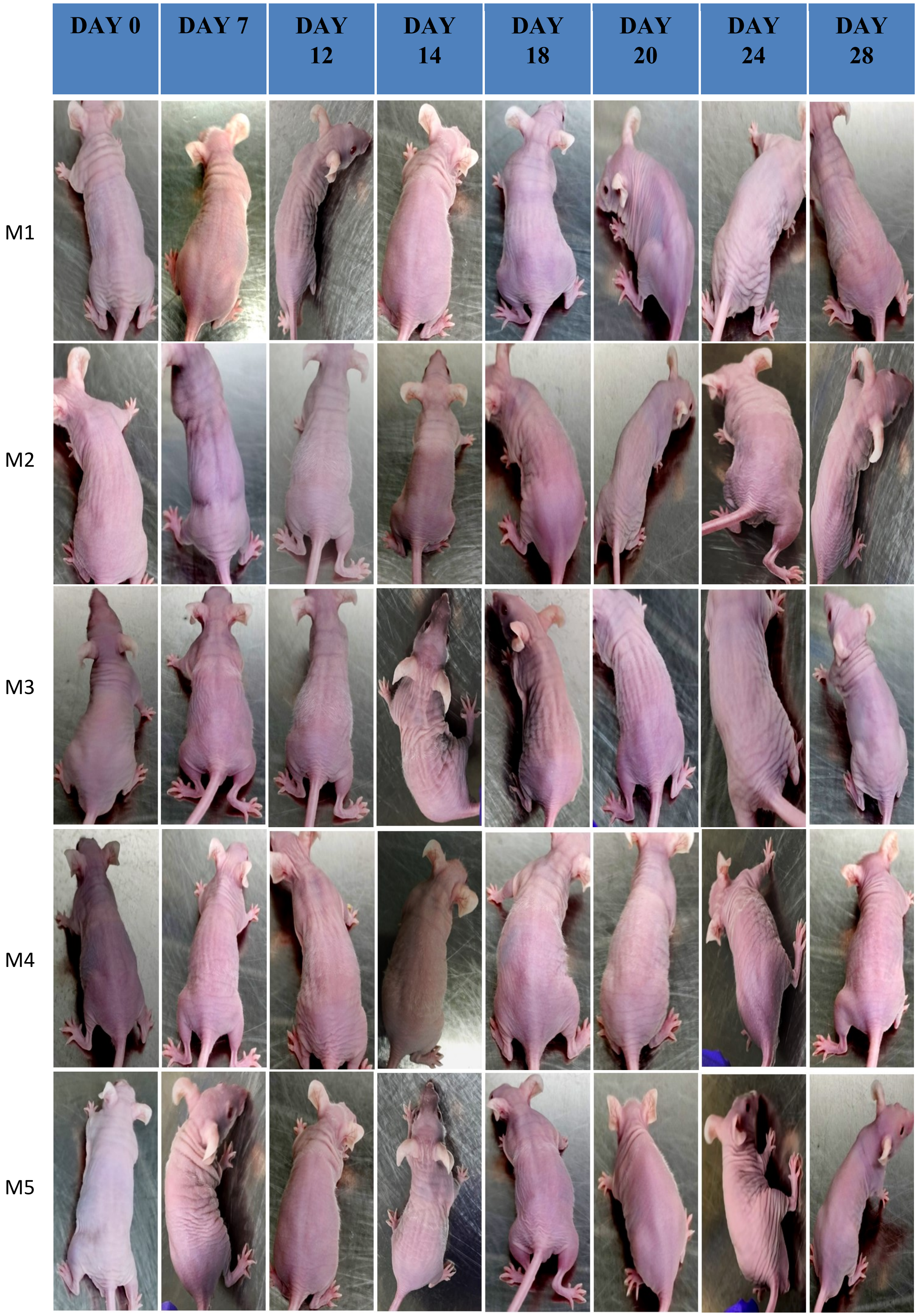

Fig S4-iv

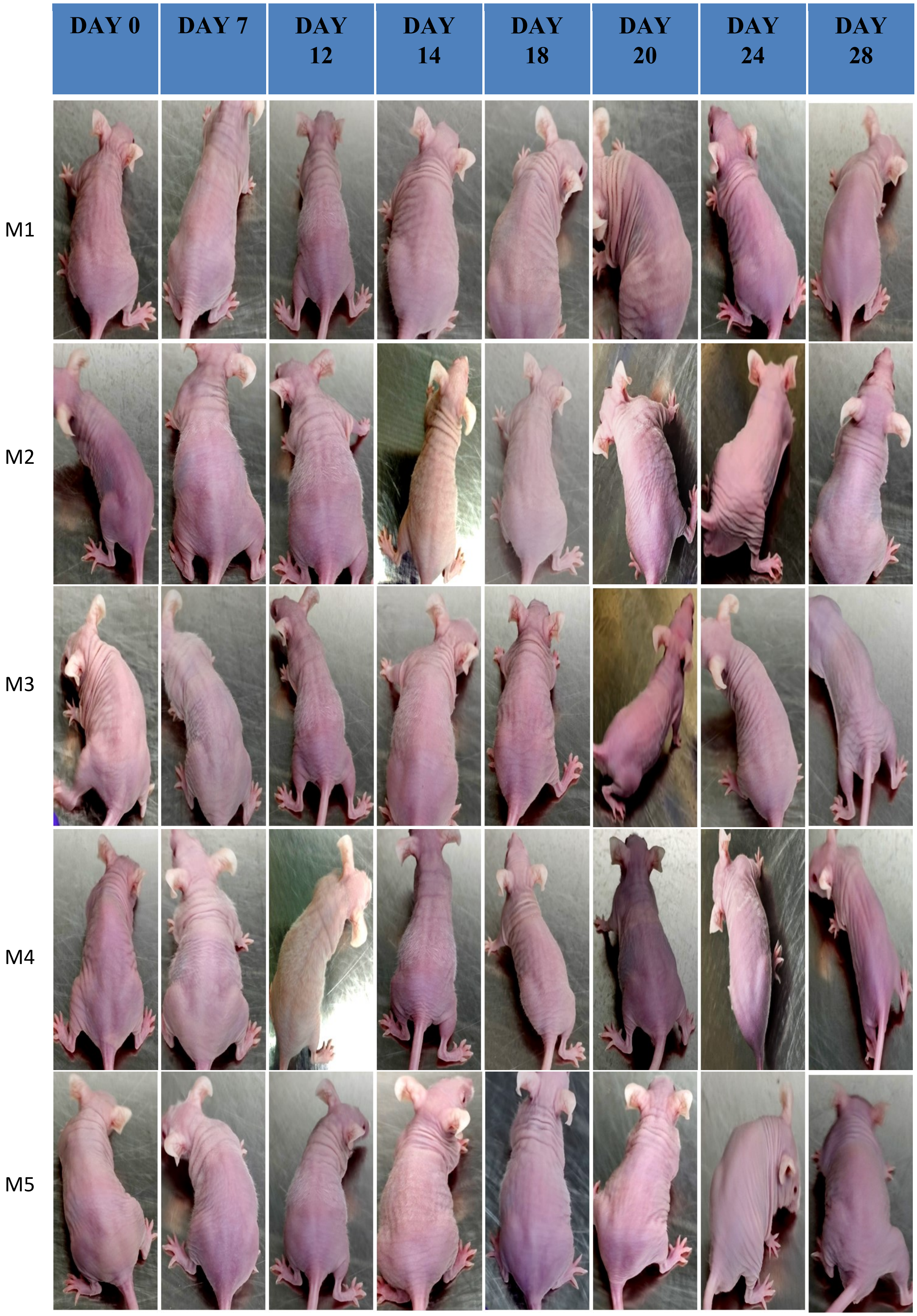

Fig S4-v

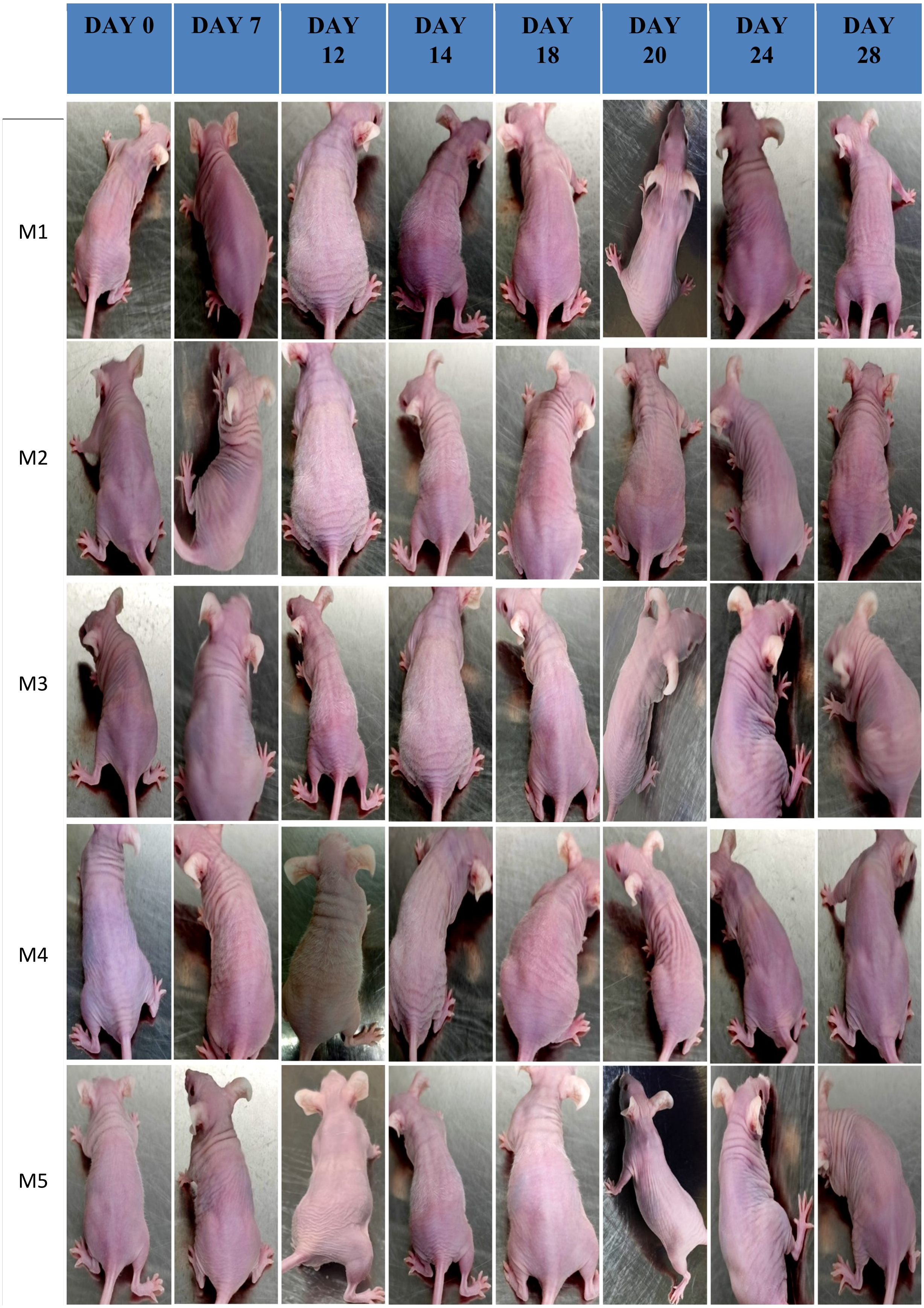

Fig S4-vi

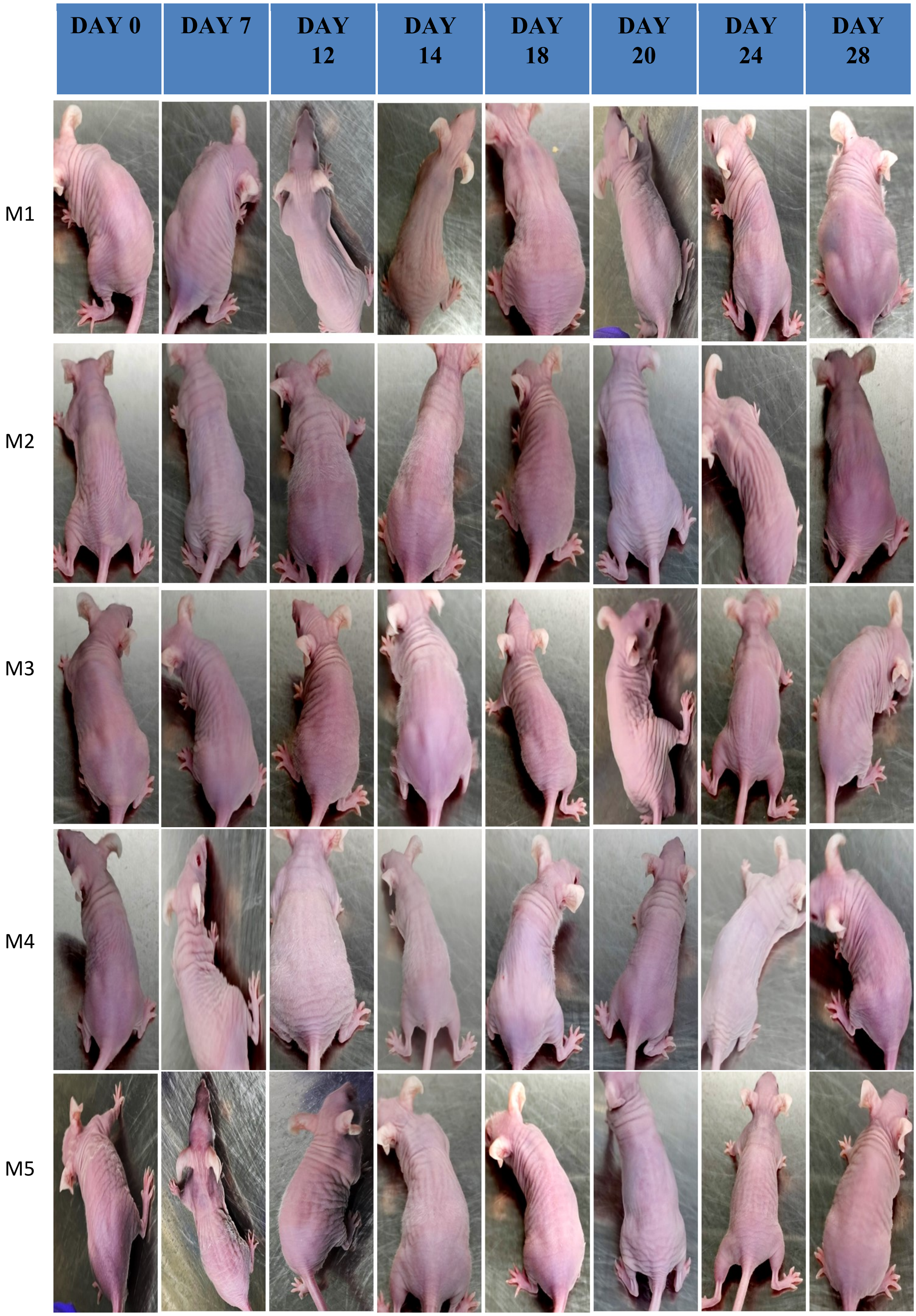

Fig S4-vii

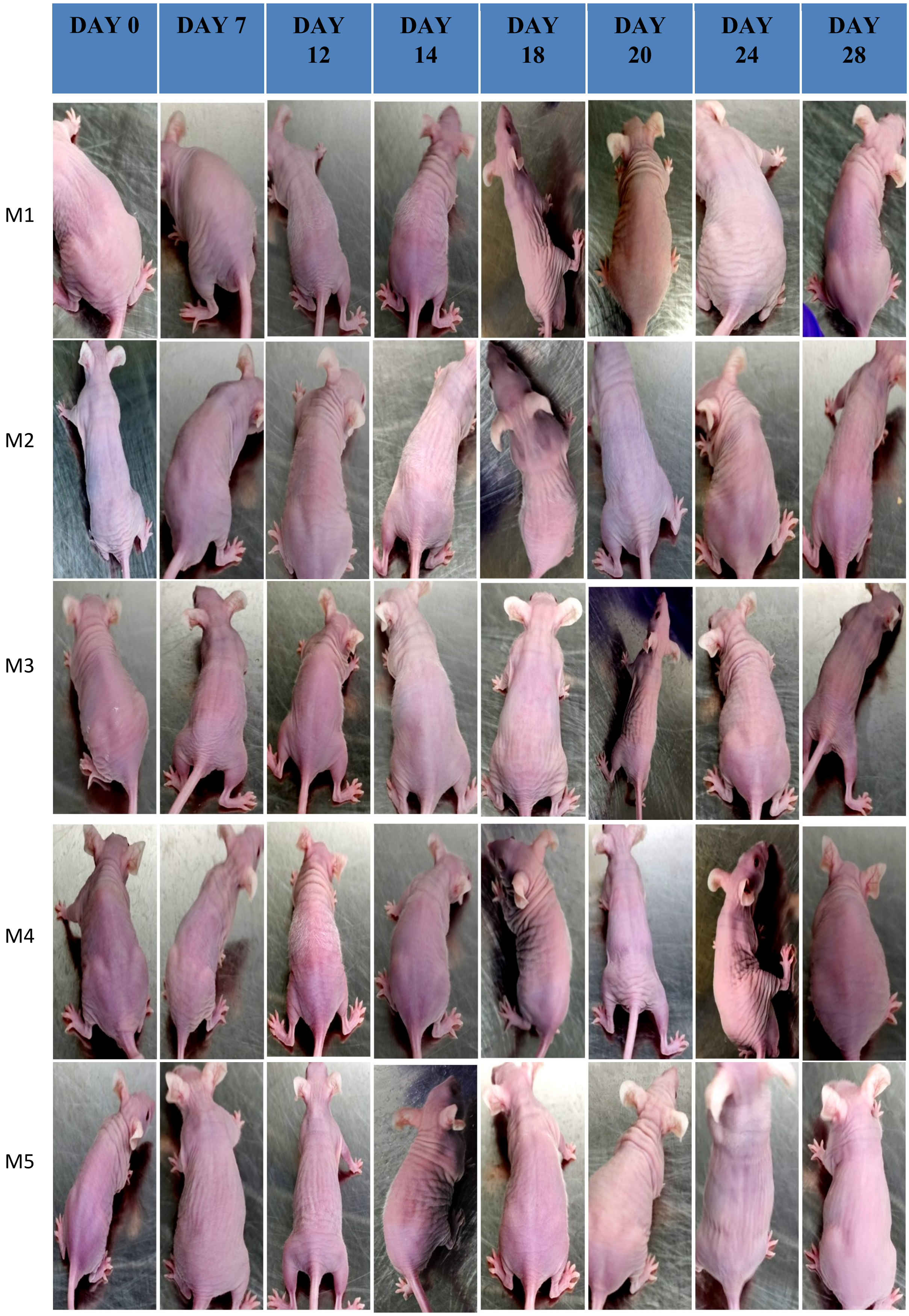

Fig S4-viii

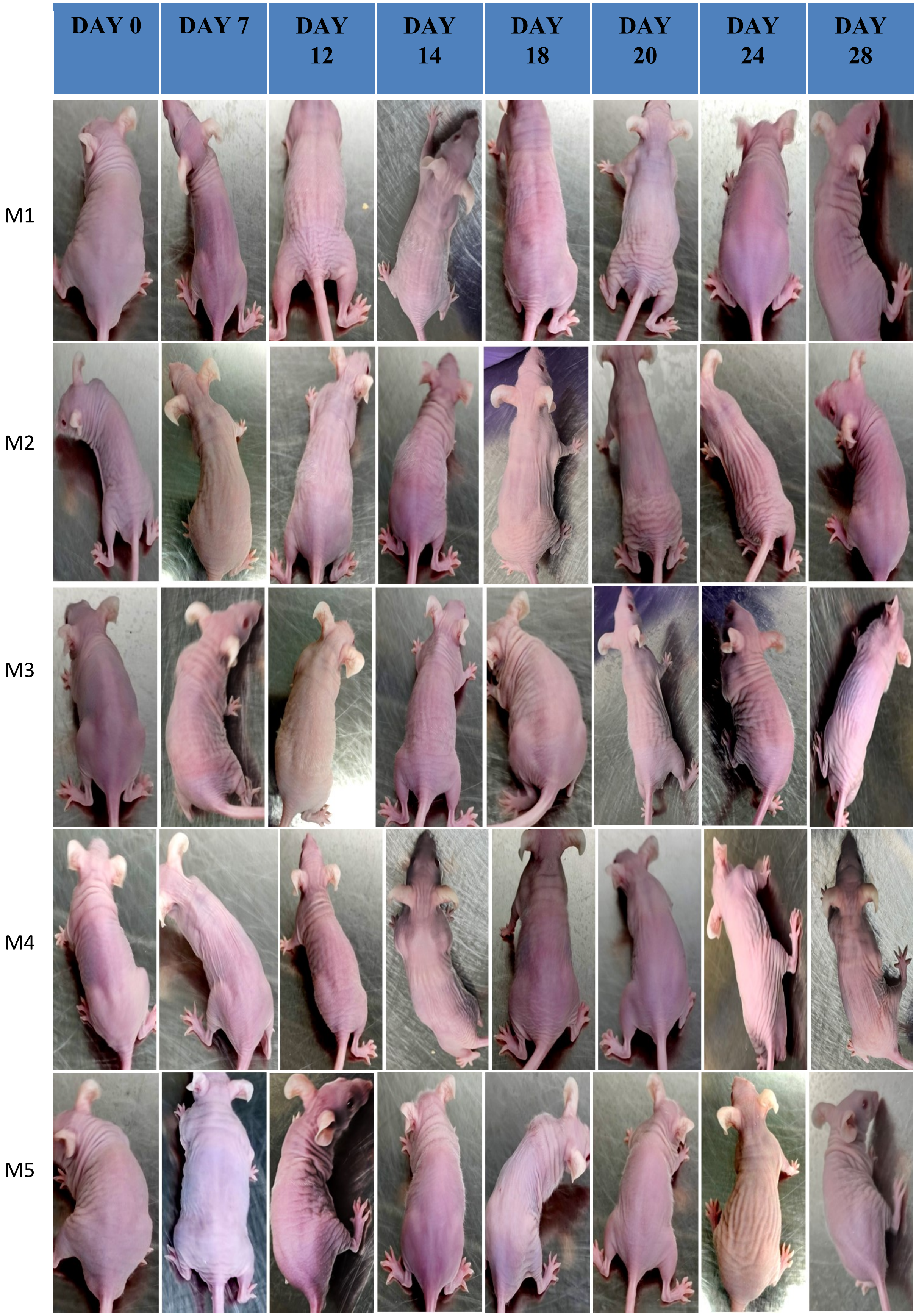

Fig S4-ix

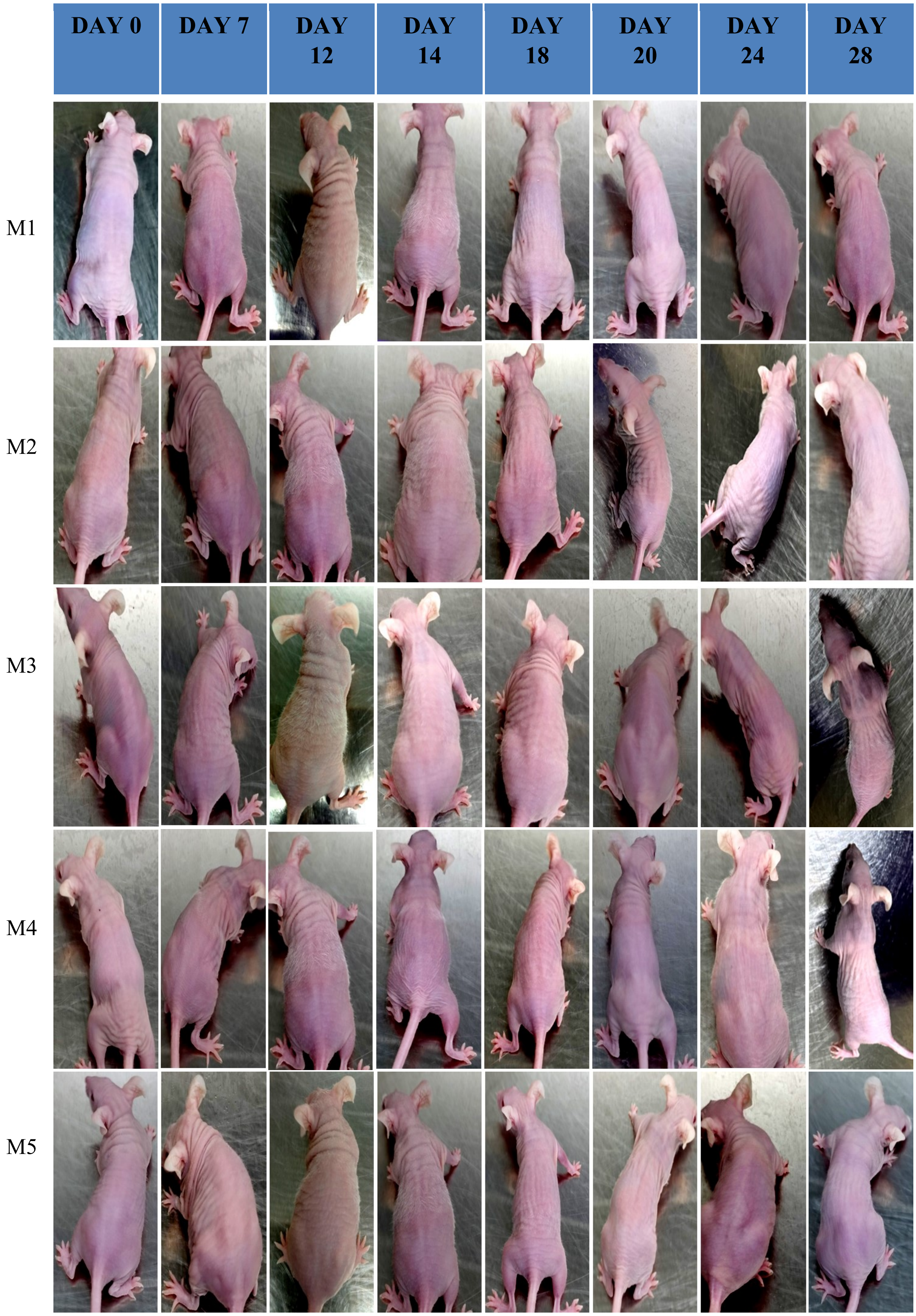

Fig S4-x

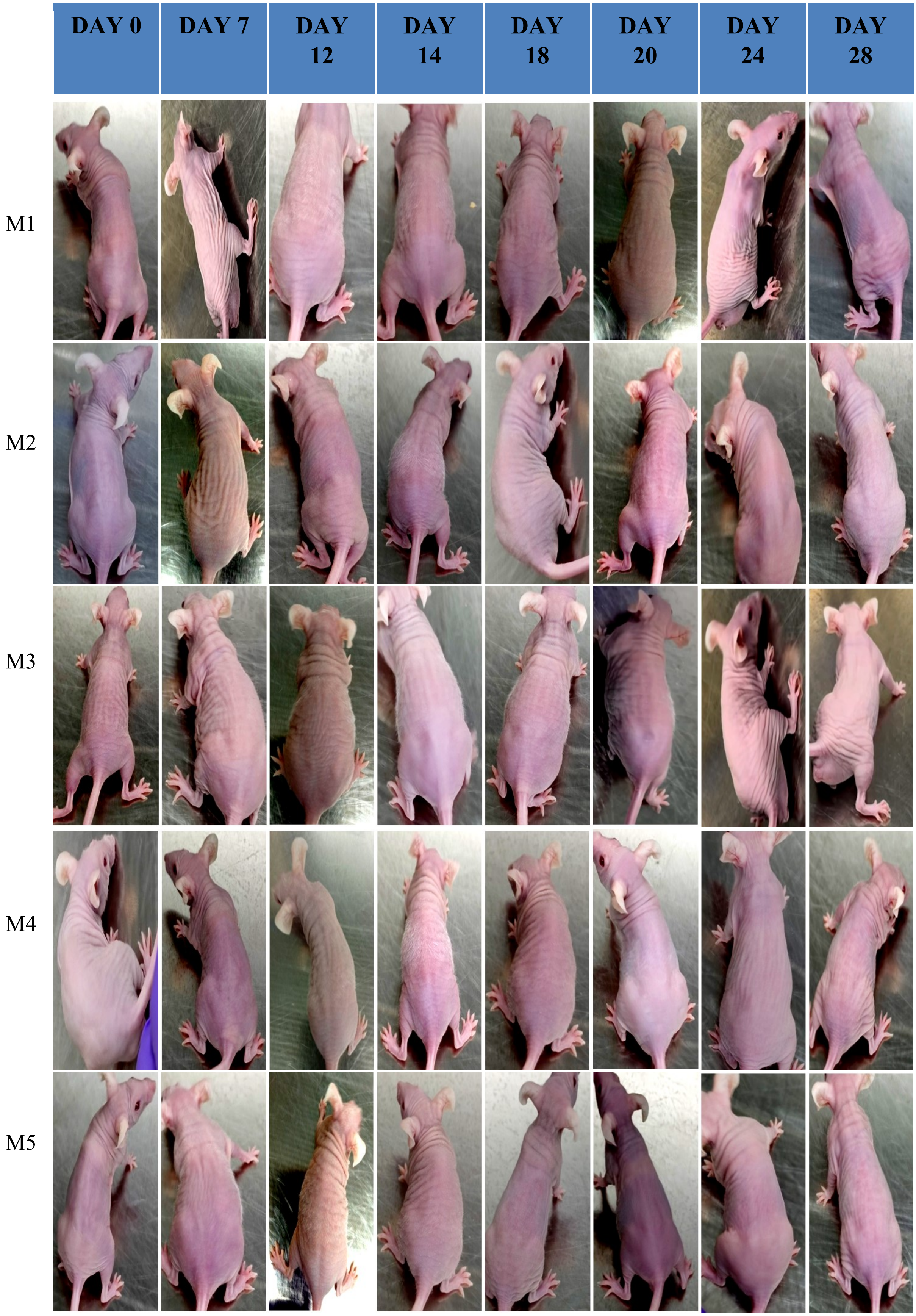

Fig 4 S1: MJ-04 induces early onset in anagen development and hair cycle progression in nude mice (NU/J Foxn1nu). (A) MJ04 induced telogen-anagen transition, as shown by the dorsal skin turning from hairless to white hair. Digital photographs were taken from the representative area using a Nikon digital camera (n = 5 mice). Fig S4(i)- Control group, Fig S4(ii)-Vehicle group, Fig S4(iii)- Tofacitinib group (0.8mg/Kg), Fig S4(iv)- Tofacitinib group (0.08 mg/kg), Fig S4(v)- MJ04 group (0.08 mg/kg), Fig S4(vi) MJ04 group (0.04 mg/kg), Fig S4(vii) MJ04 group (0.016 mg/kg), Fig S4(viii) Baricitinib group (0.1 mg/kg), Fig S4(ix) Baricitinib group (0.04 mg/kg), and Fig S4(x) Baricitinib group (0.02 mg/kg).

S4 Table 1: Weight of nude mice (NU/J Foxn1nu).

	DAY 1	DAY 7	DAY 14	DAY 21	DAY 28
**Control**	17.9	19.2	20.6	22.1	23.9
	18.3	19.5	21.1	22.6	24.8
	18.5	19.7	21.4	22.9	25.1
	18.6	20.1	21.4	23.4	25.4
	19.1	20.7	22.4	23.7	25.6
**Vechicle**	18.1	19.4	20.7	22.4	24.3
	18.5	20.1	21.5	22.9	25.1
	18.7	20.1	21.7	22.8	24.9
	18.8	20.3	22.2	23.4	25.3
	19.3	20.9	22.3	23.7	25.8
**Tofacitinib (0.8 mg/kg)**	18.1	19.3	21.1	23.1	24.5
	18.2	19.8	21.4	23.3	24.6
	18.4	20.1	21.8	23.7	25.3
	18.7	20.5	22.3	23.9	25.4
	19.3	20.5	22.4	24.1	26.1
**Tofacitinib (0.8 mg/kg)**	18.4	19.3	21.1	22.6	24.8
	18.5	19.5	21.4	22.9	25.1
	18.5	19.5	21.3	23.4	25.3
	18.9	20.6	21.8	23.4	25.4
	19.3	20.7	22.1	23.8	26.1
**MJ04 (0.08 mg/kg)**	17.9	19.7	21.4	22.8	24.9
	18.5	20.1	21.9	23.4	25.3
	18.7	20.3	22.1	23.4	25.4
	18.8	20.8	22.3	23.8	25.7
	19.4	21.1	22.7	24.3	26.1
**MJ04 (0.04 mg/kg)**	18.5	19.7	20.9	22.4	24.5
	18.6	19.8	21.1	22.8	24.7
	18.7	20.3	21.4	23.3	25.3
	18.8	20.5	21.6	23.4	25.7
	19.3	21.5	22.8	24.3	26.1
**MJ04 (0.016 mg/kg)**	18.4	19.8	21.4	22.7	24.6
	18.6	20.1	21.4	22.8	24.8
	18.8	20.3	21.5	23.1	25.3
	19.2	20.7	21.7	23.8	25.7
	19.3	20.8	22.7	24.1	26.1
	18.5	19.7	21.3	22.6	24.8
	18.6	19.8	21.3	23.2	25.3

**Baricitinib (0.1 mg/kg)**	18.7	20.1	21.8	23.6	25.4
	18.8	20.4	22.1	23.8	25.7
	19.3	21.1	22.9	24.3	25.8
**Baricitinib (0.04 mg/kg)**	17.9	19.2	20.8	22.4	24.6
	18.5	19.8	20.9	22.6	24.8
	18.6	20.1	21.4	23.1	25.3
	18.7	20.1	21.5	23.3	25.4
	19.1	20.5	21.8	23.5	25.7
**Baricitinib (0.02 mg/kg)**	17.9	19.4	21.2	22.6	24.3
	18.3	19.8	21.3	22.8	24.8
	18.5	20.2	21.7	23.2	24.9
	18.7	20.4	22.1	24.2	25.8
	19.2	20.7	22.5	24.4	26.2

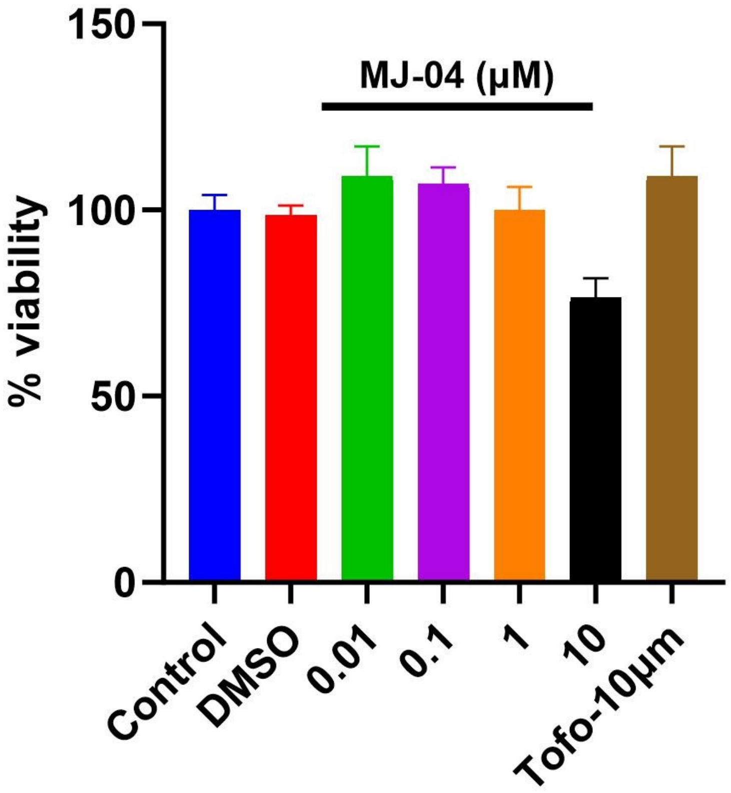

Fig S5: Evaluation of toxicity of MJ04 and tofacitinib in splenocytes by MTT assay.

S6A

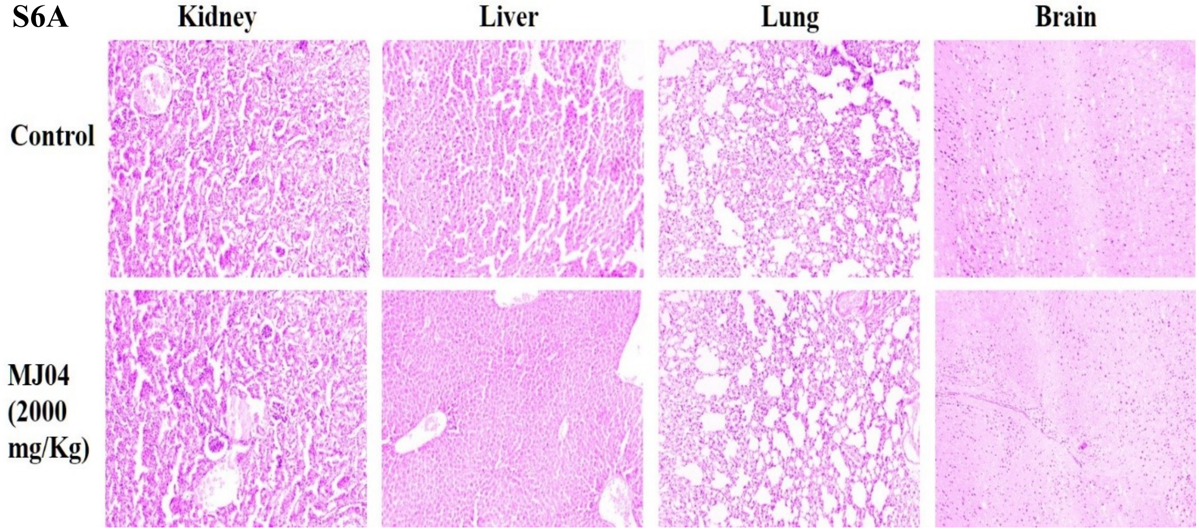

S6B

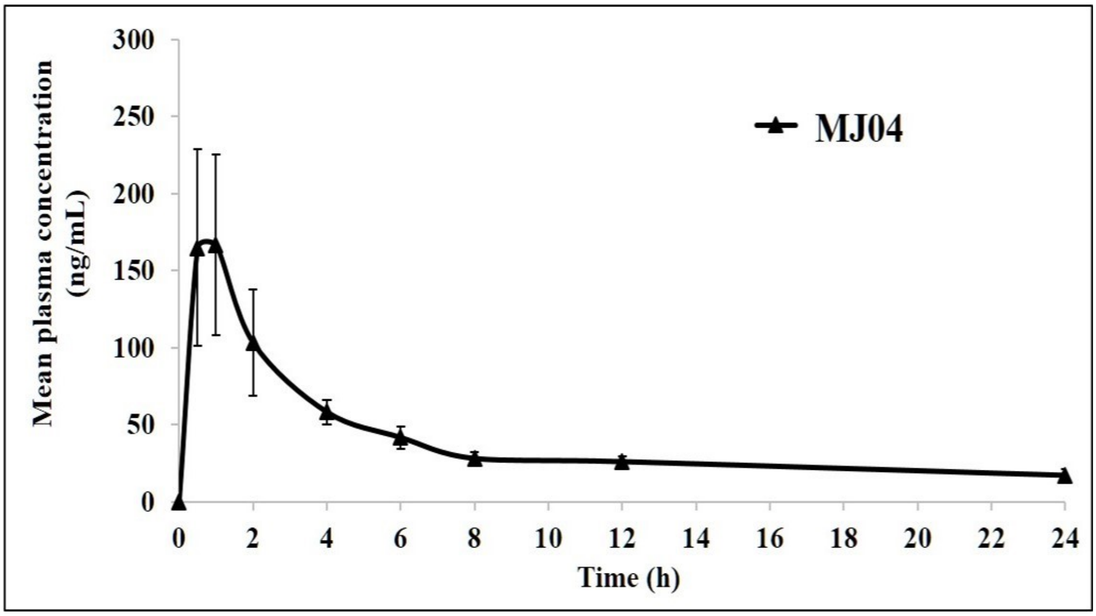

Fig. S6 (A) Histological images of organs from subacute toxicity experiment. Organs stained by H&E was obtained from the heart, liver, lung, and brain of Wistar rats after treatment with or without MJ04 (2000 mg/Kg). **(B)** Pharmacokinetic profile of MJ04, illustrating its dynamic behavior and disposition in C57BL/6 mice over a defined period of time.

ADMET profile of MJ04

ADMETlab 2.0

Fc1ccc(-c2cnc3[nH]cc(-c4cc[nH]c(=NC5CC5)n4)c3c2)cc1

1. Physicochemical Property

Property	Value	Comment
Molecular Weight	345.14	Contain hydrogen atoms. Optimal:100~600
Volume	346.381	Van der Waals volume
Density	0.996	Density = MW / Volume
nHA	5	Number of hydrogen bond acceptors. Optimal:0~12
nHD	2	Number of hydrogen bond donors. Optimal:0~7
nRot	3	Number of rotatable bonds. Optimal:0~11
nRing	5	Number of rings. Optimal:0~6
MaxRing	9	Number of atoms in the biggest ring. Optimal:0~18
nHet	6	Number of heteroatoms. Optimal:1~15
fChar	0	Formal charge. Optimal:-4 ~4
nRig	26	Number of rigid bonds. Optimal:0~30
Flexibility	0.115	Flexibility = nRot /nRig
Stereo Centers	0	Optimal: ≤ 2
TPSA	69.72	Topological Polar Surface Area. Optimal:0~140
logS	-3.522	Log of the aqueous solubility. Optimal: -4~0.5 log mol/L
logP	4.282	Log of the octanol/water partition coefficient. Optimal: 0~3
logD	3.709	logP at physiological pH 7.4. Optimal: 1~3

2. Medicinal Chemistry

Property	Value	Decision	Comment
QED	0.592	🔴	■ A measure of drug-likeness based on the concept of desirability;<br>■ Attractive: > 0.67; unattractive: 0.49~0.67; too complex: < 0.34
SAscore	3.012	🟢	■ Synthetic accessibility score is designed to estimate ease of synthesis of drug-like molecules.<br>■ SAscore ≥ 6, difficult to synthesize; SAscore <6, easy to synthesize
Fsp3	0.15	🔴	■ The number of sp3 hybridized carbons / total carbon count, correlating with melting point and solubility.<br>■ Fsp^3 ≥0.42 is considered a suitable value.
MCE-18	54.261	🟢	■ MCE-18 stands for medicinal chemistry evolution.<br>■ MCE-18≥45 is considered a suitable value.

ADMETlab 2.0

NPscore	-0.782	-	■ Natural product-likeness score.<br>■ This score is typically in the range from –5 to 5. The higher the score is, the higher the probability is that the molecule is a NP.
Lipinski Rule	Accepted	🟢	■ MW ≤ 500; logP ≤ 5; Hacc ≤ 10; Hdon ≤ 5<br>■ If two properties are out of range, a poor absorption or permeability is possible, one is acceptable.
Pfizer Rule	Rejected	🔴	logP > 3; TPSA < 75<br>Compounds with a high log P (>3) and low TPSA (<75) are likely to be toxic.
GSK Rule	Rejected	🔴	■ MW ≤ 400; logP ≤ 4<br>■ Compounds satisfying the GSK rule may have a more favorable ADMET profile
Golden Triangle	Accepted	🟢	■ 200 ≤ MW ≤ 50; -2 ≤ logD ≤ 5<br>■ Compounds satisfying the Golden Triangle rule may have a more favorable ADMET profile.
PAINS	0 alerts	-	Pan Assay Interference Compounds, frequent hitters, Alpha-screen artifacts and reactive compound.
ALARM NMR	0 alerts	-	Thiol reactive compounds.
BMS	0 alerts	-	Undesirable, reactive compounds.
Chelator Rule	0 alerts	-	Chelating compounds.

3. Absorption

Property	Value	Decision	Comment
Caco-2 Permeability	-4.555	🟢	Optimal: higher than -5.15 Log unit
MDCK Permeability	8e-06	🟢	■ low permeability: < 2 × 10^{-6} cm/s<br>■ medium permeability: 2–20 × 10^{-6} cm/s<br>■ high passive permeability: > 20 × 10^{-6} cm/s
Pgp-inhibitor	0.116	🟢	■ Category 1: Inhibitor; Category 0: Non-inhibitor;<br>■ The output value is the probability of being Pgp-inhibitor
Pgp-substrate	0.994	🔴	■ Category 1: substrate; Category 0: Non-substrate;<br>■ The output value is the probability of being Pgp-substrate
HIA	0.006	🟢	■ Human Intestinal Absorption<br>■ Category 1: HIA+(HIA < 30%); Category 0: HIA-(HIA < 30%); The output value is the probability of being HIA+
$F_{20\%}$	0.007	🟢	■ 20% Bioavailability<br>■ Category 1: $F_{20\%}$+ (bioavailability < 20%); Category 0: $F_{20\%}$- (bioavailability ≥ 20%); The output value is the probability of being $F_{20\%}$+

ADMETlab 2.0

$F_{30\%}$	0.234	●	■ 30% Bioavailability<br>■ Category 1: $F_{30\%}$+ (bioavailability < 30%); Category 0: $F_{30\%}$- (bioavailability ≥ 30%); The output value is the probability of being $F_{30\%}$+

4. Distribution

Property	Value	Decision	Comment
PPB	94.19%	●	■ Plasma Protein Binding<br>■ Optimal: < 90%. Drugs with high protein-bound may have a low therapeutic index.
VD	2.236	●	■ Volume Distribution<br>■ Optimal: 0.04-20L/kg
BBB Penetration	0.193	●	■ Blood-Brain Barrier Penetration<br>■ Category 1: BBB+; Category 0: BBB-; The output value is the probability of being BBB+
Fu	4.960%	●	■ The fraction unbound in plasms<br>■ Low: <5%; Middle: 5~20%; High: > 20%

5. Metabolism

Property	Value	Comment
CYP1A2 inhibitor	0.878	■ Category 1: Inhibitor; Category 0: Non-inhibitor;<br>■ The output value is the probability of being inhibitor.
CYP1A2 substrate	0.885	■ Category 1: Substrate; Category 0: Non-substrate;<br>■ The output value is the probability of being substrate.
CYP2C19 inhibitor	0.409	■ Category 1: Inhibitor; Category 0: Non-inhibitor;<br>■ The output value is the probability of being inhibitor.
CYP2C19 substrate	0.043	■ Category 1: Substrate; Category 0: Non-substrate;<br>■ The output value is the probability of being substrate.
CYP2C9 inhibitor	0.303	■ Category 1: Inhibitor; Category 0: Non-inhibitor;<br>■ The output value is the probability of being inhibitor.
CYP2C9 substrate	0.038	■ Category 1: Substrate; Category 0: Non-substrate;<br>■ The output value is the probability of being substrate.
CYP2D6 inhibitor	0.656	■ Category 1: Inhibitor; Category 0: Non-inhibitor;<br>■ The output value is the probability of being inhibitor.
CYP2D6 substrate	0.315	■ Category 1: Substrate; Category 0: Non-substrate;<br>■ The output value is the probability of being substrate.
CYP3A4 inhibitor	0.644	■ Category 1: Inhibitor; Category 0: Non-inhibitor;<br>■ The output value is the probability of being inhibitor.
CYP3A4 substrate	0.23	■ Category 1: Substrate; Category 0: Non-substrate;<br>■ The output value is the probability of being substrate.

ADMETlab 2.0

6. Excretion

Property	Value	Decision	Comment
CL	6.676	●	■ Clearance<br>■ High: >15 mL/min/kg; moderate: 5-15 mL/min/kg; low: <5 mL/min/kg
$T_{1/2}$	0.126	-	■ Category 1: long half-life ; Category 0: short half-life;<br>■ long half-life: >3h; short half-life: <3h<br>■ The output value is the probability of having long half-life.

7. Toxicity

Property	Value	Decision	Comment
hERG Blockers	0.977	●	■ Category 1: active; Category 0: inactive;<br>■ The output value is the probability of being active.
H-HT	0.989	●	■ Human Hepatotoxicity<br>■ Category 1: H-HT positive(+); Category 0: H-HT negative(-);<br>■ The output value is the probability of being toxic.
DILI	0.908	●	■ Drug Induced Liver Injury.<br>■ Category 1: drugs with a high risk of DILI; Category 0: drugs with no risk of DILI. The output value is the probability of being toxic.
AMES Toxicity	0.55	●	■ Category 1: Ames positive(+); Category 0: Ames negative(-);<br>■ The output value is the probability of being toxic.
Rat Oral Acute Toxicity	0.981	●	■ Category 0: low-toxicity; Category 1: high-toxicity;<br>■ The output value is the probability of being highly toxic.
FDAMDD	0.943	●	■ Maximum Recommended Daily Dose<br>■ Category 1: FDAMDD (+); Category 0: FDAMDD (-)<br>■ The output value is the probability of being positive.
Skin Sensitization	0.607	●	■ Category 1: Sensitizer; Category 0: Non-sensitizer;<br>■ The output value is the probability of being sensitizer.
Carcinogencity	0.067	●	■ Category 1: carcinogens; Category 0: non-carcinogens;<br>■ The output value is the probability of being toxic.
Eye Corrosion	0.003	●	■ Category 1: corrosives ; Category 0: noncorrosives<br>■ The output value is the probability of being corrosives.
Eye Irritation	0.011	●	■ Category 1: irritants ; Category 0: nonirritants<br>■ The output value is the probability of being irritants.

ADMETlab 2.0

Respiratory Toxicity	0.918	●	■ Category 1: respiratory toxicants; Category 0: respiratory nontoxicants<br>■ The output value is the probability of being toxic.

8. Environmental toxicity

Property	Value	Comment
Bioconcentration Factors	1.506	■ Bioconcentration factors are used for considering secondary poisoning potential and assessing risks to human health via the food chain.<br>■ The unit is –log10[(mg/L)/(1000*MW)]
IGC_{50}	4.426	■ Tetrahymena pyriformis 50 percent growth inhibition concentration<br>■ The unit is –log10[(mg/L)/(1000*MW)]
$LC_{50}FM$	5.966	■ 96-hour fathead minnow 50 percent lethal concentration<br>■ The unit is –log10[(mg/L)/(1000*MW)]
$LC_{50}DM$	6.6	■ 48-hour daphnia magna 50 percent lethal concentration<br>■ The unit is –log10[(mg/L)/(1000*MW)]

9. Tox21 pathway

Property	Value	Decision	Comment
NR-AR	0.015	●	■ Androgen receptor<br>■ Category 1: actives ; Category 0: inactives;<br>■ The output value is the probability of being active.
NR-AR-LBD	0.005	●	■ Androgen receptor ligand-binding domain<br>■ Category 1: actives ; Category 0: inactives;<br>■ The output value is the probability of being active.
NR-AhR	0.922	●	■ Aryl hydrocarbon receptor<br>■ Category 1: actives ; Category 0: inactives;<br>■ The output value is the probability of being active.
NR-Aromatase	0.969	●	■ Category 1: actives ; Category 0: inactives;<br>■ The output value is the probability of being active.
NR-ER	0.345	●	■ Estrogen receptor<br>■ Category 1: actives ; Category 0: inactives;<br>■ The output value is the probability of being active.
NR-ER-LBD	0.388	●	■ Estrogen receptor ligand-binding domain<br>■ Category 1: actives ; Category 0: inactives;<br>■ The output value is the probability of being active.
NR-PPAR-gamma	0.566	●	■ Peroxisome proliferator-activated receptor gamma<br>■ Category 1: actives ; Category 0: inactives;<br>■ The output value is the probability of being active.
SR-ARE	0.697	●	■ Antioxidant response element<br>■ Category 1: actives ; Category 0: inactives;<br>■ The output value is the probability of being active.
SR-ATAD5	0.551	●	■ ATPase family AAA domain-containing protein 5<br>■ Category 1: actives ; Category 0: inactives;<br>■ The output value is the probability of being active.

ADMETlab 2.0

SR-HSE	0.345		■ Heat shock factor response element<br>■ Category 1: actives ; Category 0: inactives;<br>■ The output value is the probability of being active.
SR-MMP	0.723		■ Mitochondrial membrane potential<br>■ Category 1: actives ; Category 0: inactives;<br>■ The output value is the probability of being active.
SR-p53	0.859		■ Category 1: actives ; Category 0: inactives;<br>■ The output value is the probability of being active.

10. Toxicophore Rules

Property	Value	Comment
Acute Toxicity Rule	0 alerts	■ 20 substructures<br>■ acute toxicity during oral administration
Genotoxic Carcinogenicity Rule	0 alerts	■ 117 substructures<br>■ carcinogenicity or mutagenicity
NonGenotoxic Carcinogenicity Rule	1 alerts	■ 23 substructures<br>■ carcinogenicity through nongenotoxic mechanisms
Skin Sensitization Rule	0 alerts	■ 155 substructures<br>■ skin irritation
Aquatic Toxicity Rule	1 alerts	■ 99 substructures<br>■ toxicity to liquid(water)
NonBiodegradable Rule	2 alerts	■ 19 substructures<br>■ non-biodegradable
SureChEMBL Rule	0 alerts	■ 164 substructures<br>■ MedChem unfriendly status

ADMETlab 2.0
